# Comparative mitochondrial genome and transcriptome analyses reveal strain-specific features of RNA editing in *Trypanosoma brucei*

**DOI:** 10.1093/nar/gkaf661

**Published:** 2025-07-16

**Authors:** Xiaojing Zhao, Yixin He, Fan Zhang, Inna Aphasizheva, Ruslan Aphasizhev, Liye Zhang

**Affiliations:** School of Life Science and Technology, ShanghaiTech University, Shanghai201210, China; School of Life Science and Technology, ShanghaiTech University, Shanghai201210, China; School of Life Science and Technology, ShanghaiTech University, Shanghai201210, China; Department of Molecular and Cell Biology, Boston University Medical Campus, Boston, MA 02118, United States; Department of Molecular and Cell Biology, Boston University Medical Campus, Boston, MA 02118, United States; School of Life Science and Technology, ShanghaiTech University, Shanghai201210, China; Shanghai Clinical Research and Trial Center, Shanghai 201210, China

## Abstract

*Trypanosoma brucei*, a kinetoplastid parasite, cycles between a tsetse fly vector and a mammalian host, undergoing profound changes in cell architecture and metabolism. Central to these transitions are modifications in mitochondrial structure, volume, and energy production. The parasite’s mitochondrial genome is highly complex, comprising a few maxicircles that encode proteins and rRNAs, and thousands of minicircles that encode guide RNAs (gRNAs). Most messenger RNAs (mRNAs) sustain gRNA-directed U-insertion/deletion editing to acquire functional protein-coding sequences. Although the minicircle repertoire varies among isolates and environmental conditions, the extent and biological significance of this variability in commonly used laboratory strains remain unclear. Here, we analyzed mitochondrial genomes and transcriptomes of the developmentally competent AnTat1.1 strain, the differentiation-incapable Lister 427 strain, and transgenic derivatives of Lister 427. While maxicircle sequences are broadly conserved and stable, minicircles differ markedly in sequence complexity, relative abundance, and gRNA gene content. These variations likely affect the efficiency and accuracy of mRNA editing. Compared with Lister 427, the AnTat1.1 strain retains greater minicircle diversity, longer predicted gRNA–mRNA duplexes, and higher editing fidelity. By examining cell lines with distinct developmental capacities and cultivation histories, our findings reveal how mitochondrial genomes evolve in response to changing environmental contexts.

## Graphical Abstract

**Graphical Abstract:**
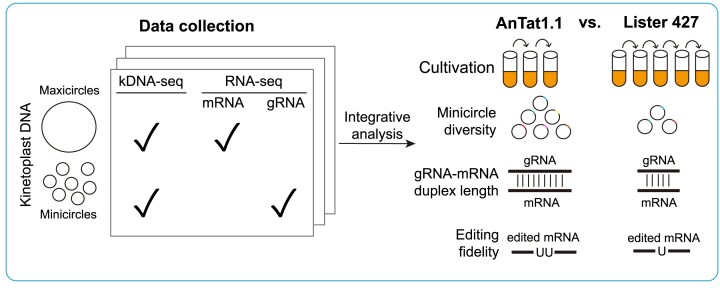

## Introduction

*Trypanosoma brucei*, a parasitic protist belonging to the class Kinetoplastea, causes African trypanosomiasis, commonly referred to as sleeping sickness in humans and nagana in animals. This disease threatens millions of individuals and imposes significant economic burdens across sub-Saharan Africa [1]. *Trypanosoma brucei* contains a distinctive mitochondrial nucleoprotein structure called the kinetoplast, housing kinetoplast DNA (kDNA), a compact network of catenated maxicircles and minicircles [2]. The kinetoplast genome typically comprises several ∼23-kb-long maxicircles and ∼6000 minicircles (∼1 kb each). Minicircles constitute roughly 90% of the kDNA mass and have uniform length, but display considerable diversity in their sequences and copy numbers among different strains. Maxicircles encode 2 rRNAs, 1 gRNA, 6 protein-coding genes, and 12 pseudogenes [3]. The protein-coding genes—NADH dehydrogenase (ND) subunits 1, 4, and 5, cytochrome oxidase (CO) subunit 1, ribosomal protein uS3m, and mitochondrial unidentified open reading frame 1 (MURF1)—produce “never-edited” mRNAs (messenger RNAs), meaning they are capable of programming protein synthesis directly (Table 1). Conversely, the ∼900 guide RNAs (gRNAs), mostly derived from minicircles [4–7], direct mRNA editing that transforms 12 pseudogene transcripts (“pre-edited” mRNAs) into functional, “fully edited” mRNAs [8]. The extent of editing varies, ranging from the insertion of four uridines correcting a frameshift to massive mRNA recoding involving hundreds of U-insertions and deletions per transcript, a phenomenon termed pan-editing [9]. Out of the ∼1500 nuclear-encoded proteins imported into the mitochondrion [10], nearly 25% participate in the production of 18 predicted kDNA-encoded proteins.

**Table 1.** Glossary of terms

Term	Definition
Pre-edited mRNA	A pseudogene transcript that requires editing to encode a protein
Fully edited mRNA	Editing product containing the longest predicted open reading frame [34]
Never-edited mRNA	Contains an open reading frame encoded in DNA
Editing site	Position between two non-Ts in the cDNA versus T-less maxicircle DNA sequence
T-less position	The coordinate in the non-T sites, with all Ts (Us) removed from the mRNA
U-stretch length	Number of Ts between non-Ts, matching editing sites in reads or reference
BSF	Bloodstream form
PCF	Procyclic (insect) form
BF/SM	Lister 427 bloodstream cell lines
29-13/PF	Lister 427 procyclic cell lines
AnTat1.1	AnTat1.1 strain in bloodstream form
Editing ratio/level	Number of reads with U-length distinct from the pre-edited mRNA divided by the read coverage at a given site
Canonical site	Site with a differing number of Us between pre-edited and fully edited mRNA
Noncanonical site	Site with same U-stretch length between pre-edited and fully edited mRNA
Consistency rate	The fraction of canonical sites with a number of Us matching the reference
sRNA	gRNA-sized small RNA that does not conform to the gRNA definition
kDNA	Kinetoplast DNA
gRNA family	Distinct gRNAs directing the same editing patterns in the same mRNA region
Duplex length	The length of a complementary gRNA/mRNA region, two mismatches allowed

*Trypanosoma brucei* adapts to varying environmental conditions, nutrient availability, and immune responses during its digenetic life cycle, alternating between the mammalian host and the tsetse fly vector. The efficiency of editing fluctuates not only among different transcripts but also for the same mRNA during developmental transitions or adaptation to axenic cultivation. However, the relationship between mRNA editing patterns and the functional necessity of the corresponding protein remains poorly understood. For instance, ATP synthase subunit 6 (A6) [11] and ribosomal protein uS12m [12] mRNAs undergo consistent pan-editing, and their protein products are predicted to be essential for Complex V and ribosome activities, respectively, in both bloodstream and insect (procyclic) forms. In contrast, transcripts encoding NADH dehydrogenase subunits ND3, ND7, ND8, and ND9 exhibit more extensive editing in the bloodstream form, despite canonical respiratory Complex I being present but dispensable for viability in both life stages [13, 14]. In the mammalian host, the parasite primarily generates ATP via glycolysis rather than oxidative phosphorylation and relies on Complex V to hydrolyze ATP for maintaining mitochondrial membrane potential [11].

The kinetoplast genomes of trypanosomatids exhibit a remarkable diversity in size, number, and sequence complexity of DNA circles, degree of catenation, and the abundance of gRNA genes [15]. Notably, the physical size of the network and the quantity of constituent minicircles are tightly regulated, whereas the diversity of minicircle sequence classes can decline when parasites are cultivated for an extended period of time [16]. The definition of a sequence class also varies. Simpson *et al.* defined a class as a group of homologous *Leishmania tarentolae* minicircles encoding gRNAs of the same editing capacity [17]. Whereas different gRNAs can direct identical editing events by pairing with the pre-edited and edited mRNAs through canonical G-C/A-U or wobble G-U interactions, such redundant gRNA sets are defined as gRNA families. Since *T. brucei* minicircles typically harbor one to four gRNAs, we adopted the definition of a sequence class as a group of minicircles sharing ≥95% sequence similarity, consistent with a previous study [5]. The most abundant minicircle within each class will be used as the representative for that sequence class.

As the most tractable model among kinetoplastid parasites, *T. brucei* is predominantly studied using laboratory-adapted, monomorphic (incapable of differentiation) procyclic or bloodstream forms of the Lister 427 strain and their respective transgenic derivatives, “29-13” and “single marker” (SM) cell lines. The Cross laboratory developed the 29-13 cell line by integrating neomycin and hygromycin resistance, T7 phage RNA polymerase, and tetracycline repressor (TetR) genes into the nuclear genome. The SM cell line also expresses T7 RNA polymerase and TetR, and carries neomycin resistance [18]. These transgenic lines have significantly advanced genetic and biochemical studies by enabling inducible RNA synthesis from DNA fragments under the control of the TetR-regulated promoter. For simplicity, we will refer to these cell lines as PF (Lister 427 procyclic form), BF (Lister 427 bloodstream form), 29-13 (derivative of Lister 427 PF), and SM (derivative of Lister 427 BF). Terms “PCF” and “BSF” will refer to the developmental stage regardless of genetic modification. Additionally, the pleomorphic (capable of differentiation in culture) AnTat1.1 (EATRO 1125) strain and its transgenic derivatives have been utilized in studies of antigenic variation and other aspects of trypanosome biology. The minicircle and gRNA repertoires were characterized in AnTat1.1, revealing ∼400 minicircle sequence classes and 1318 small RNA genes, of which about 931 encode gRNAs [5, 19].

The study of mRNA abundance and editing during progressive differentiation of AnTat1.1 from BSF to PCF in axenic culture established the overall upregulation of mitochondrial transcripts, concomitant with elevated oxidative phosphorylation and mitochondrial enlargement [20]. These findings suggest a significant role for post-transcriptional regulation, while the rapid timescale of differentiation renders substantial changes in maxicircles or minicircles unlikely. Conversely, cross-strain comparisons may shed light on mitochondrial genome plasticity during extended cultivation and help clarify whether the reduction in kDNA complexity contributes to the loss of differentiation capacity.

Here, we tested the hypothesis that long-term selective pressures favoring rapid cell proliferation in culture lead to streamlining of mitochondrial genomes and transcriptomes and investigated the efficiency and fidelity of RNA editing across the most common laboratory cell lines. By assembling and annotating both maxicircle and minicircle genomes and their corresponding transcriptomes, we found that AnTat1.1 displays a greater number of variants in protein-coding maxicircle sequences and maintains a significantly more diverse minicircle repertoire than Lister 427. However, these variations in kDNA do not significantly affect fully edited mRNA sequences or their predicted translation products. Furthermore, we demonstrate that the presence or absence of specific minicircles, their relative abundance, and the length of complementarity between gRNAs and edited mRNAs are associated with distinct stage- and cell line-specific editing patterns and editing fidelity.

## Materials and methods

### *Trypanosoma brucei* cultivation

The AnTat1.1 strain was maintained in long slender bloodstream form at densities below 1 × 10^6^ cells/ml by daily dilutions in modified HMI-9 medium supplemented with 15% heat-inactivated fetal bovine serum (FBS) and 1.1% methylcellulose, at 37°C and 5% CO_2_ in T-flasks, typically no longer than 2 weeks [21]. Bloodstream Lister 427 and its SM transgenic derivative were cultured under the same conditions without methylcellulose; SM cultures also contained 5 μg/ml G418. The procyclic form was maintained at a concentration of <10^7^ cells/ml at 27°C in SDM-79 medium supplemented with 10% heat-inactivated FBS in round flasks with mild agitation. For 29-13, 50 μg/ml G418 and 50 μg/ml hygromycin B were also included.

### Nucleic acid isolation and sequencing library preparation

To isolate kDNA networks, 2–5 × 10^8^ parasites were collected by centrifugation for 15 min at 3000 × *g* and washed twice in 1/10 of the original culture volume with PBS, supplemented with 10 mM ethylenediaminetetraacetic acid (EDTA). The cell pellet was resuspended in 10 ml of 100 mM Tris–HCl (pH 8.0) and 10 mM EDTA, and 0.1 mg/ml proteinase K. Lysis was induced by adding sodium dodecyl sulfate to 0.5%. After incubation at 55°C for 2 h with gentle rocking, the lysate was passed through a 12-gauge needle using a 10-ml syringe to shear nuclear DNA and then loaded into an SW41 tube (Beckman). kDNA was pelleted by centrifugation at 65 000 × *g* for 1.5 h at 18°C, resuspended in 0.2 ml of TE buffer containing 0.1 mg/ml RNase A, and incubated for 60 min with mild agitation. The sample was then loaded into an SW41 tube containing two equal-volume CsCl layers in TE buffer: a light layer (refractive index = 1.3705) and a heavy layer (refractive index = 1.404, supplemented with 0.14 mg/ml ethidium bromide). After centrifugation at 50 000 × *g* for 20 min, the kDNA band was visualized under UV light at the interface between the light and heavy layers and extracted by side puncture. The kDNA was diluted ∼10-fold with TE buffer to ∼11 ml and collected by centrifugation under the same conditions for 2 h. The kDNA pellet was resuspended in 1 ml of TE, extracted once with an equal volume of phenol/chloroform, and precipitated with ethanol. After washing with 80% ethanol, the purified kDNA was dissolved in TE at ∼0.1 mg/ml and fragmented using a Covaris M220 Focused Ultrasonicator at 75 W peak incident power and a 20% duty factor in 200-cycle mode. The fragmented DNA was processed using the TruSeq DNA PCR-Free Sample Preparation Kit (Illumina) according to the manufacturer’s instructions and sequenced on an Illumina MiSeq system in 300-bp paired-end mode.

For small RNA sequencing, mitochondria were enriched by hypotonic lysis, differential centrifugation, and flotation in Renocal 76 density gradient [22]. RNA was extracted from mitochondria and fractionated on a 10% polyacrylamide–8 M urea PAGE. RNAs migrating between a 40-nt synthetic marker and the tRNA fraction (∼75 nt) were excised and converted into RNA-seq libraries using the NEBNext Small RNA Library Prep Set for Illumina (NEB).

For global RNA sequencing, mitochondrial RNA was processed with NEBNext Ultra II RNA Library Prep Kit for Illumina, including on-bead fragmentation to obtain ∼200–400-nt fragments. Poly(A)^+^ selection and rRNA depletion were omitted. Random oligonucleotide-primed reverse transcription was performed at 40°C to enhance representation of U-rich mRNAs. Multiplexed libraries were sequenced on a NovaSeq 6000 system in 150-bp paired-end mode.

### kDNA-seq data preprocessing

To remove reads originating from the nuclear genome, the kDNA reads were first mapped to the *T. brucei* TREU927 nuclear genome from the TriTrypDB database release 48 [23] (https://tritrypdb.org/common/downloads/release-48/TbruceiTREU927/fasta/data/) using bwa-mem2 v2.0pre2 [24] with default parameters. The unmapped reads were extracted using samtools v1.4.1 [25] and converted to fastq files with *SamToFastq* from gatk v4.0.5.1 [26]. The fastq reads were trimmed using fastp v0.23.2 [27] with the parameter “-q 30 -u 10 -5 -3 -W 1 -M 30 --cut_right --cut_right_window_size 10 --cut_right_mean_quality 30 -l 100 -b 125”.

### Maxicircle assembly

KOMICS package v1.1.8 [28] with recommended maxicircle parameter “--kmin 29 –kmax 119 –kstep 20” was used to assemble preliminary contigs. The contigs with lengths longer than 10 kb were used as seeds for elongation with NOVOplasty v4.3.1 [29] against the maxicircle reference (GenBank: M94286.1). We compared the assembled maxicircles with reference from two vantage points. First, the kDNA reads were mapped to assembled maxicircles and the maxicircle reference. Then, MUMmer (version 3.23) [30] was used to show the similarity between the assembled maxicircle and the maxicircle reference. According to the results, we made some modifications to the assembled maxicircles. The region absent in the maxicircle reference for the strain BF could be aligned to the annotated minicircles and, therefore, deleted. The cell line-specific variants were called based on the assembled maxicircle sequences.

### Minicircle assembly

The KOMICS v1.1.8 [28] package was used to automatically assemble the minicircles with *assemble* parameter “--kmin 99 --kmax 119 --kstep 10”, default *circularize* parameter, and *polish* parameter “--minidentity 97”. To polish the output contig of the minicircle, customized scripts based on the partial CSB1 sequence (GGGCGT) were used to split contigs that may be assembled from multiple circular minicircles.

The trimmed reads were aligned to the first-round minicircle assembly with bwa-mem2, and the unmapped reads were extracted with *samtools*. These reads were then subjected to the same trimming, KOMICS, and contig splitting procedures. The final contigs were merged with the first assembly, and contigs smaller than 900 nt were removed. To remove redundancy, *cd-hit-est* [31] and *blastn* (https://blast.ncbi.nlm.nih.gov/Blast.cgi) with a 95% similarity threshold were used, resulting in the final minicircle assembly. To identify minicircles derived from the same ancestry across multiple cell lines, minicircle contigs from five cell lines were mapped to each other using bwa-mem2. Any two contigs sharing at least 400 bp have been combined into the same group with custom scripts.

After the minicircle reference was assembled, the kDNA-seq reads were aligned to the separated reference using novoalign v3.09.00 (http://www.novocraft.com/products/novoalign/). CPM (counts per million) was utilized to normalize the counts per minicircle for each cell line.

### Phylogenetic analyses of minicircles

The rooted phylogenetic tree was generated based on minicircle sequence alignment using DNAMAN software (version 6.0.3.99). The process involved (i) multiple sequence alignment using DNAMAN and (ii) phylogenetic tree generation employing the maximum likelihood method as the distance model, with a bootstrap value of 1000 to assess the robustness of the tree topology.

### Small RNA-seq data processing

Small RNA reads were mapped to the nuclear genome and then trimmed using fastp. To remove noise from other RNAs, forward reads ending with four Ts (Us in the RNA sense strand) were selected and then trimmed using fastp.

### gRNA prediction

The filtered forward reads were mapped to assembled minicircles (https://gitee.com/Zhanglab/minicircle_maxcircle_ strain_cmp/tree/master/data-deposit/minicircle) using novoalign v3.09.00. The aligned reads were extracted and sorted, followed by peak calling using Cufflinks v2.2.1 on the Watson and Crick strands separately [32]. Since most gRNAs are flanked by inverted repeats, peaks without a forward inverted repeat sequence (TAATA[GA]AT) within 150 nt upstream were discarded. The gRNAs were predicted by aligning the gRNA peak sequences to fully edited mRNAs (https://gitee.com/Zhanglab/minicircle_maxcircle_strain_cmp/tree/master/data-deposit/canonical%20mRNA%20sequences) using LCS [7], with the following parameters: a minimum match length of 24 bp, at least 70% similarity, and a maximum of two mismatches. The alignment score was calculated to determine the cognate mRNA for each gRNA. In cases where a gRNA aligned to multiple mRNAs, the mRNA with the longest alignment was retained. The read count matrix for each gRNA was generated using featureCounts (version 2.0.3). CPM normalization was applied to account for differences in sequencing depth.

### gRNA analysis

We performed a three-tier gRNA analysis: sequence classes (diversity), gene copy number, and expression (abundance). For sequence classes, we identified all predicted gRNA classes targeting each mRNA, counted the number of unique gRNAs per mRNA, and divided by the total number of gRNAs. For the gene copy number, we summed the CPM of minicircles encoding gRNAs targeting each mRNA and divided by 1 000 000. For gRNA expression, CPMs of all gRNAs targeting each mRNA were summed and divided by 1 000 000. If a gRNA targeted multiple mRNAs, it was assigned to the mRNA with the longest alignment. In cases of multiple mRNAs sharing the longest alignment, the CPM was divided equally. For strain comparisons, log_2_ ratios of the summed CPMs for each mRNA were calculated to represent relative gRNA abundance.

### mRNA-seq data processing

The mitochondrial mRNA sequencing data from AnTat1.1, BF, and PF (two replicates each) were processed to remove sequencing reads that mapped to the nuclear genome. The editing profiles of 12 edited mRNAs lacking all Us were generated using Tless-alignment-toolkit (https://github.com/bioliyezhang/RESC_paper/tree/v1.0.0) [33]. The editing ratios in editing sites were calculated as (read count that exhibited non-pre-edited mRNA U-length pattern)/(read count that exhibited non-pre-edited mRNA U-length pattern + read count that exhibited pre-edited mRNA U-length pattern) × 100%.

### Consistency of U-stretches analysis at canonical editing sites

To avoid the noise from low sequencing depth, editing sites covered by at least 200 sequencing reads were selected for U-length consistency analysis. Consistency was assessed by examining the length of the U-stretch at canonical sites between two non-U bases for each read; the number of reads supporting each observed U-length was summed. The most abundant U-length, excluding the maxicircle DNA-encoded Us, was identified. A site was classified as consistent if the most abundant U-length matched the fully edited reference U-length [34].

## Results

### The divergence of maxicircle genes between Lister 427 and AnTat1.1 has a minor impact on the protein-coding capacity of edited mRNAs

**Figure 1.**
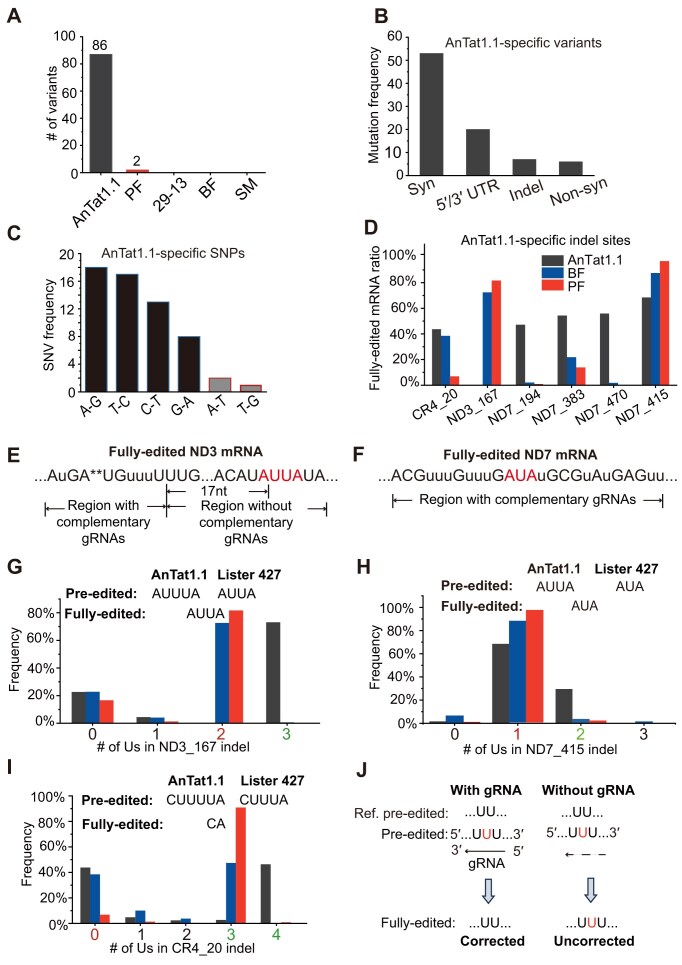
Sequence divergence of mitochondrial mRNAs and rRNAs between AnTat1.1 and Lister 427 cell lines. (**A**) Variations from the maxicircle reference sequence from EATRO 427, which is a derivative of the Lister 427 strain (GenBank M94286.1), across 18 mRNA and two rRNA genes. (**B**) Frequencies of variant categories specific to AnTat1.1. (**C**) Frequencies of single-nucleotide variants (SNVs) specific to AnTat1.1. (**D**) Ratios of fully edited mRNAs associated with AnTat1.1-specific insertions and deletions (indels). The *x*-axis denotes genes harboring indels and their T-less coordinates (positions after removal of all encoded Us and those inserted by RNA editing). (**E**) The T-less position 167 in the ND3 mRNA is not a canonical site. Fully edited reference ND3 mRNA sequences are shown, with inserted Us indicated by “u” and deleted Us by “*”. (**F**) The region surrounding the T-less position 415 in the ND7 mRNA, annotated as in panel (E). (**G**) A bar chart showing the number of Us in the ND3 T-less site at position 167. The *x*-axis shows the number of consecutive Us between two non-U nucleotides. Red numbers indicate numbers of Us in fully edited mRNAs; green numbers represent pre-edited mRNAs (AnTat1.1). When U-stretch counts in both transcript types (fully edited and pre-edited) are identical at a given position, the U-stretch number is also colored in red. For instance, both fully edited and pre-edited transcripts in Lister 427 encode two Us in this position; thus 2 on *x*-axis is colored red. Bar charts depicting the U-stretch variation for an indel in the ND7 gene (**H**) and the CR4 gene (**I**). (**J**) Schematic of the gRNA’s potential compensatory role in resolving indels in fully edited mRNAs.

We performed deep sequencing on fragmented kDNA from five cell lines (Table 2) and assembled their maxicircle and minicircle genomes using KOMICS [35]. In all cases, the longest contig corresponded to the maxicircle, with sizes ranging from 17.8 to 19.5 kb (Supplementary Table S1). The maxicircles from each cell line contained the conserved region, which included a cluster of 18 protein-coding genes and pseudogenes, as well as 9S and 12S rRNAs, and a gene encoding a gRNA that edits the mitochondrial unidentified open reading frame 2 (MURF2) mRNA. Pairwise alignments confirmed that our assemblies partially covered the divergent regions [36] (Supplementary Fig. S1). Given the high conservation of maxicircle coding regions within the Trypanosomatid family [37], we called variants in the five cell lines and annotated those that may affect the gene products. As shown in Fig. 1A and Table 3, the AnTat1.1 strain exhibited the largest number of variants (*n* = 86), including base substitutions and indels, relative to the Lister 427 reference generated by Sanger sequencing of cloned maxicircle DNA (GenBank: M94286.1; TRBKPGEN locus). Among the four Lister 427 cell lines, only the PF contained two variants in the protein-coding regions (Supplementary Fig. S2 and Supplementary Table S2). Most variants specific to AnTat1.1 (∼98%, 84 out of 86) were previously identified [5], with only two unique ones found in the untranslated regions (UTRs) (Supplementary Table S2).

**Table 2.** Summary of the sequencing data replicates

	Cell lines				
	AnTat1.1	Lister 427			
		BF	SM	PF	29-13
Developmental form	BSF	BSF	BSF	PCF	PCF
kDNA-seq	1	1	1	1	1
Small RNA-seq	1	1	1	1	1
mRNA-seq	2	2	N/A	2	N/A

**Table 3.** Number of SNPs/indels in maxicircle transcripts

SNPs/indels	AnTat1.1	PF	29-13	BF	SM	Gene-level total
CO3	0	0	0	0	0	0
uS12m	0	0	0	0	0	0
CO2	1	0	0	0	0	1
CR3	1	0	0	0	0	1
A6	2	0	0	0	0	2
ND8	1	1	0	0	0	2
CYB	3	0	0	0	0	3
MURF2	3	0	0	0	0	3
CR4	4	0	0	0	0	4
ND3	4	0	0	0	0	4
ND9	6	0	0	0	0	6
ND7	7	0	0	0	0	7
MURF1	5	0	0	0	0	5
ND4	6	0	0	0	0	6
uS3m	8	0	0	0	0	8
CO1	10	1	0	0	0	11
ND1	11	0	0	0	0	11
ND5	11	0	0	0	0	11
9S rRNA	1	0	0	0	0	1
12S rRNA	2	0	0	0	0	2
Total	86	2	0	0	0	88

Since the PF was the only monomorphic Lister 427 derivative displaying two variants, we analyzed these deviations in greater detail. A U-to-A single nucleotide polymorphism (SNP) was identified in the pseudogene whose transcript undergoes pan-editing to produce NADH dehydrogenase subunit 8 (ND8) mRNA (Supplementary Fig. S2). Since this uridine is deleted in fully edited transcripts [38], such transversion could introduce frameshift in the mature mRNA (Supplementary Fig. S3A). However, this variant may also persist given that Complex I is nonessential for PCF growth [13]. Consequently, because ND8 is not strictly required for the proliferation of monomorphic procyclic parasites [13], these two nonsynonymous mutations are unlikely to affect fitness. The second SNP was an A-to-U transversion that changed a codon from UGA (tryptophan, W) to UGU (cysteine, C) in the cytochrome oxidase I (CO1) gene. Although tryptophan is generally conserved at this position, other hydrophobic amino acids, such as leucine, have been observed in *Trypanoplasma borreli* (Supplementary Fig. S3B).

Next, we analyzed the AnTat1.1-specific variants, identifying 83 changes across 18 protein-coding genes and pseudogenes. These variants fall into four categories: synonymous mutations; SNPs and insertion/deletion (indel) events in the 5′- and 3′-UTRs; indels in coding regions; and nonsynonymous mutations. The majority were synonymous mutations (53 out of 83, ∼64%), followed by SNPs and indels in UTRs (19 out of 83, ∼23%) (Fig. 1B). In the mRNA-encoding strand, 51 of the 53 synonymous mutations were purine-to-purine or pyrimidine-to-pyrimidine transitions (Fig. 1C), which could be tolerated by G-U base pairing between the pre-mRNA and gRNA. Only five of the 83 variants were nonsynonymous A-to-G and C-to-U transitions (two A-to-G, two G-to-A, and a single C-to-U), with three occurring in the ND1 gene, one in CO2, and one in ND9. The latter gene encodes a pre-mRNA that requires pan-editing, but the mutation is unlikely to affect the gRNA binding site due to an A-to-G mutation potentially amendable by G-U pairing. The amino acids in the Lister 427 reference align with those of closely related species (Supplementary Fig. S3C–E). This strong bias toward synonymous over nonsynonymous mutations mirrors observations in *Leishmania*spp. [39], highlighting the evolutionary pressure to maintain RNA’s protein-coding integrity despite DNA sequence divergence.

Six of the 83 AnTat1.1-specific variants (∼7%) are frameshift indels involving a single U-insertion or deletion at four and two sites, respectively. At the gene level, four indels occur in ND7 and one each in CR4 and ND3. To assess their possible functional impacts, we conducted deep sequencing of mitochondrial transcriptomes from AnTat1.1, BF, and PF cell lines (Table 2), each in two biological replicates. For mRNA-seq, we have sequenced BF as a representative of BSF cell lines and PF for PCF cell lines. We calculated the percentage of fully edited transcripts adhering to canonical editing patterns, revealing that fully edited forms in AnTat1.1 decreased to nearly zero in ND3, but not in CR4 or ND7 mRNAs (Fig. 1D). Further inspection revealed that the indel in ND3 was located outside of the editing domain, ∼19 nt downstream of the last canonical site (Fig. 1E), and that the lengths of U-stretches remained unchanged in the majority (>70%) of transcripts (Fig. 1G). The U-insertions (three Us versus two in the reference) create a premature termination codon in ND3 mRNA, truncating five amino acids from the predicted C-terminus (Supplementary Fig. S3F). Similar to ND3, the ND7 indel site at T-less position 415 is not a canonical site, meaning that the U-stretches in pre-edited and fully edited mRNAs are identical (Fig. 1F and Table 1). Unlike in the case of the ND3 indel site, the complementary gRNAs are present and may edit the surrounding regions in the ND7 mRNA. Consequently, the one additional U in AnTat1.1 was corrected in over 60% of ND7 transcripts (Fig. 1H), but none in ND3 (Fig. 1G). Despite the presence of indels, AnTat1.1 also exhibited higher ratios of fully edited sequences in the other four canonical sites with complementary gRNAs present (Fig. 1I and Supplementary Fig. S3G–I). This suggests that U-stretch length variations in the maxicircle sequence can be corrected by complementary gRNAs if these are encoded in the minicircle genome (Fig. 1J).

In conclusion, we identified substantial sequence polymorphism in the 18 maxicircle-encoded protein genes and pseudogenes between AnTat1.1 and the monomorphic bloodstream and procyclic forms of Lister 427. However, most of these variants are either corrected by editing or predicted to have a minimal impact on mRNAs and their protein products. Given the limited variation in maxicircle sequences between AnTat1.1 and Lister 427, we next investigated whether the divergence of their minicircle populations is similarly constrained.

### Minicircle genome variations within and between strains

**Figure 2.**
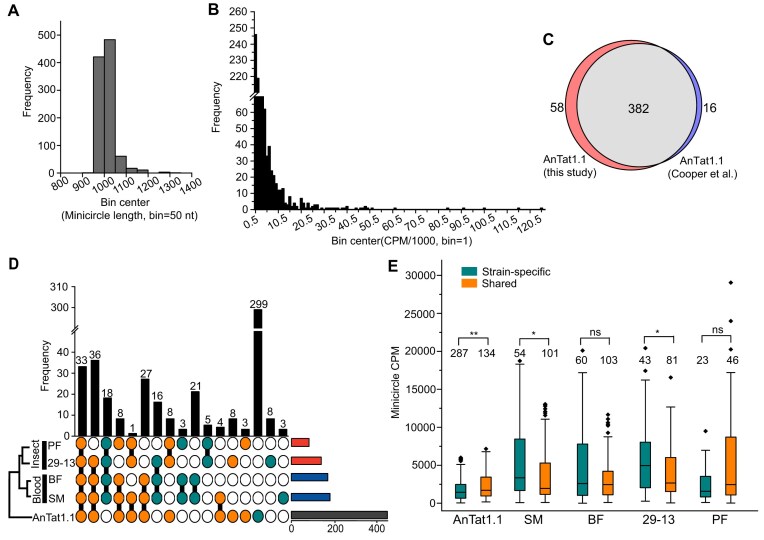
Characteristics of minicircle genomes across *T. brucei* cell lines. (**A**) Length frequency distribution of minicircles in five cell lines. (**B**) Distribution of minicircle copy numbers normalized by CPM in five cell lines. The *x*-axis values indicate bin centers; for instance, a minicircle with a CPM of 246 falls into the 0–1 bin [0 < CPM/1000 (246/1000) < 1]. (**C**) Overlap of AnTat1.1 minicircles assembled in this study with those by Cooper *et al.* [5] (**D**) An UpSet plot showing shared and unique minicircles among AnTat1.1 and four monomorphic cell lines. Below the *x*-axis, filled and hollow circles indicate the presence or absence of minicircles, respectively. Orange circles highlight minicircles shared between Lister 427 and AnTat1.1, while green circles represent those unique to respective strains. The y-axis shows the number of minicircles in each category. The horizontal bar indicates the number of minicircles across cell lines. AnTat1.1 has 299 unique minicircles compared to 26–61 of Lister 427-specific minicircles. (**E**) A boxplot comparing the relative abundances of strain-specific minicircles with those shared between AnTat1.1 and Lister 427. The numbers of shared and strain-specific minicircle classes are displayed above the boxplots. Statistical significance was evaluated using the Mann–Whitney–Wilcoxon test with Benjamini–Hochberg correction; **P < .01, *P  < .05. ns: not significant. Two-sided *P-*values were used, unless specified.

Quality evaluation of the minicircle assemblies showed close adherence to the expected size of ∼1 kb (Fig. 2A and Supplementary Table S3), facilitating subsequent analyses of their frequency distributions. To validate assembly quality, we compared the AnTat1.1 minicircles sequenced in this study with those reported by Cooper *et al.* [5] and observed over 90% overlap (Fig. 2C), with most minicircle classes occurring at low copy numbers per kDNA network (Fig. 2B and Supplementary Fig. S4A). Grouping the minicircles from all five cell lines based on sequence similarity showed that the two bloodstream and two procyclic Lister 427 cell lines clustered together (Fig. 2D). The number of Lister 427 minicircle sequence classes ranged from 79 to 177, which aligns with the previous estimate of fewer than 200 minicircle classes [40]. The BF and SM exhibited nearly identical minicircle compositions, with 162 shared versus 8 unique (Fig. 2D), as well as similar abundance patterns (Supplementary Fig. S4B). In contrast, the PF and 29-13 lines displayed greater divergence, with 65 shared genes versus 82 unique ones (Fig. 2D and Supplementary Fig. S4B). These observations suggest that axenic growth conditions impose distinct selective pressures on maxicircle and minicircle genomes, as evidenced by reduced sequence complexity and substantial heterogeneity of the minicircles among monomorphic cell lines.

More pronounced differences were observed between the AnTat1.1 and Lister 427 strains, with AnTat1.1 exhibiting a substantially higher number of strain-specific minicircles (Fig. 2D). Differences in sequencing depth are unlikely to explain this observation, as AnTat1.1 had the fewest number of reads among all strains (Supplementary Table S1). To investigate potential mechanisms underlying these differences, we hypothesized that minicircles shared between AnTat1.1 and Lister 427 may be selectively retained, and analyzed their representation. Minicircles were categorized as either strain-specific or shared, and their relative abundances were compared. Indeed, shared minicircles were overrepresented in AnTat1.1, while the opposite trend was observed in Lister 427 (Fig. 2E). These findings suggest that despite their lower number, Lister-specific minicircles may undergo stronger selective pressure within a streamlined genome. Conversely, the lower abundance of AnTat1.1-specific minicircles could reflect reduced selective constraints, potentially allowing a higher mutation rate that may compromise gRNA encoding capacity. To examine this possibility, we compared the number of gRNAs encoded by AnTat1.1-specific minicircles to those encoded by minicircles shared among strains and found no significant difference (Supplementary Fig. S4C and Supplementary Tables S3 and S4).

Another contributing factor to the decline of minicircle classes in laboratory-adapted Lister 427 cell lines may be their extended cultivation history, leading to the random loss of nonessential minicircles. Consistently, Cooper *et al.* [5] demonstrated a decrease in minicircle sequence classes after only 12 weeks in culture. We reasoned that if strain-specific minicircles could be found in other strains, such results would support the notion that ancestral strains contained a more complete set of minicircles. Thus, we retrieved minicircles from the EATRO 164 strain [40] and found that indeed several strain-specific minicircles from AnTat1.1 or Lister 427 are present in EATRO 164 (Supplementary Fig. S4D). In addition, two shared minicircles classes in our study were also present in EATRO 164. A further inspection of minicircle phylogenetic trees for these two shared classes among the three strains revealed that one previously an outgroup minicircle (AnTat1.1_contig321) clustered with EATRO 164, which is another example of selective cross-strain retention (Supplementary Fig. S4E).

### Editing profiles are consistent across monomorphic and pleomorphic strains

**Figure 3.**
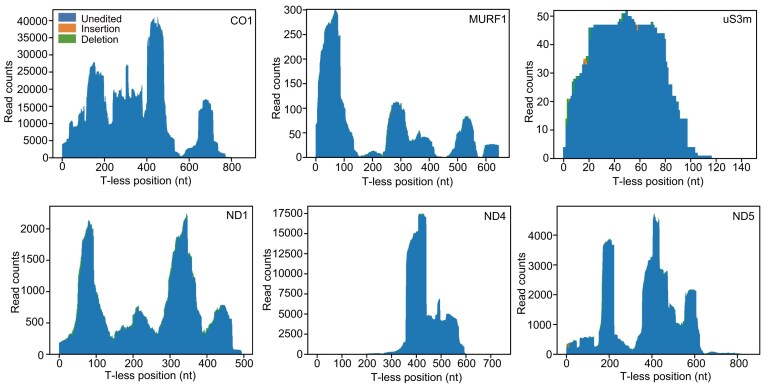
Analysis of potential RNA editing events in six never-edited transcripts in the AnTat1.1 strain. The editing frequency is shown for each T-less nucleotide position. Sites without editing are colored blue, U-insertion sites yellow, and U-deletion sites green.

To determine whether the broader gRNA repertoire associated with greater minicircle genome complexity in AnTat1.1 results in the editing of mRNAs previously described as never-edited in Lister 427 [34], we applied the TAligner pipeline [41] to six such transcripts. Given the extensive depth of mRNA-seq achieved in this study, the absence of U-insertion or deletion events in these six never-edited mRNAs across five cell lines highlights the remarkable specificity of gRNA targeting (Fig. 3 and Supplementary Fig. S5). Beyond the specificity provided by ∼11 bp gRNA anchor region’s complementarity to pre-edited mRNA, this process is likely facilitated by the RNA editing substrate binding complex [19, 33].

**Figure 4.**
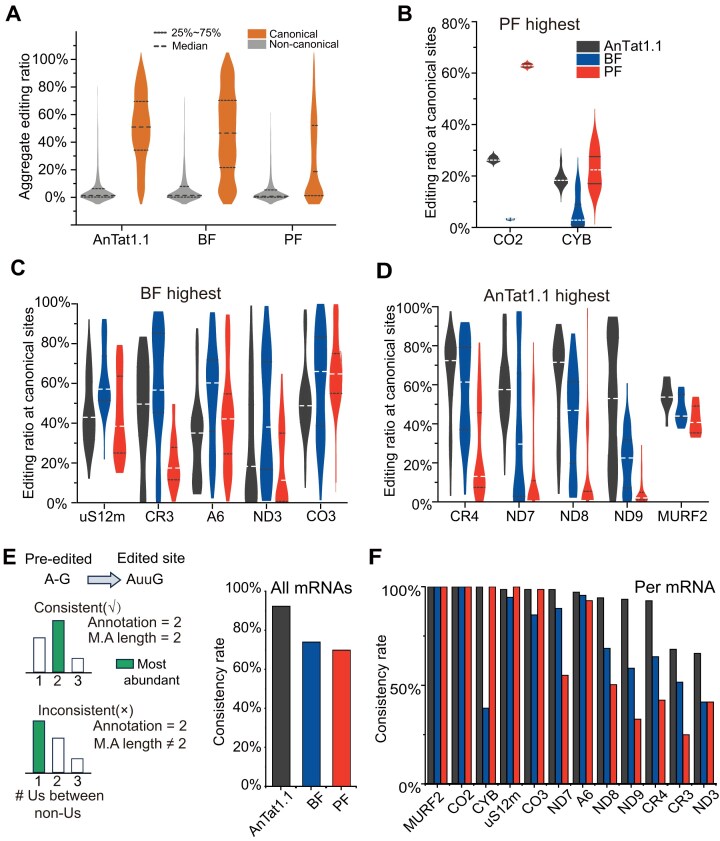
RNA editing profiles of AnTat1.1 and Lister 427 cell lines. (**A**) Violin plot of editing ratios at canonical (orange) and noncanonical (gray) sites across all edited mRNAs for each cell line. (**B**–**D**) Violin plots of canonical site editing ratios for each mRNA, grouped by the highest ratio: PF (red), BF (blue), and AnTat1.1 (black). (**E**) Left panel: definition of editing consistency at canonical sites. If the fully edited mRNA has two Us and the most abundant variant (M.A., in green) also has two Us, it is considered consistent (2 = 2, upper panel). If the most frequent variant has only one U, it is inconsistent (1 ≠ 2, lower panel). Unedited reads are excluded from determining the most abundant variant. Right panel: consistency rates in canonical sites across all edited mRNAs for each cell line, with cell lines color-coded as in panel (B). (**F**) Editing consistency rate for each edited mRNA in AnTat1.1, BF, and PF, colored as in panel (B).

Reduced minicircle genome complexity and a corresponding depletion of the gRNA population have previously been linked to partial editing loss in a long-cultured UC strain of *L. tarentolae* [16, 17, 42]. Therefore, we systematically assessed the influence of minicircle diversity on editing profiles across 12 pseudogene transcripts. Sites were classified as canonical or noncanonical based on documented editing events (Table 1). AnTat1.1 consistently exhibited editing patterns comparable to those observed in monomorphic Lister 427 cell lines, showing high editing levels at canonical sites and negligible editing at noncanonical sites (Fig. 4A and Supplementary Table S5). Upon closer analysis of canonical editing events, we observed cell line-specific variations in certain transcripts. Specifically, CO2 and CYB mRNAs showed higher editing levels in the PF cell line, while editing of the remaining 10 mRNAs was more pronounced in BSF and AnTat1.1 (Fig. 4B–D and Supplementary Fig. S6A and B).

We further validated editing fidelity by analyzing predominant U-stretch lengths at individual sites. AnTat1.1 demonstrated consistency with the Lister 427 reference, both globally and at the gene-specific level (Fig. 4E and F, Supplementary Fig. S6C, and Supplementary Table S6). Thus, despite harboring a more diverse gRNA pool than Lister 427, AnTat1.1 lacks detectable novel or alternatively edited sites.

### The presence of gRNA-encoding minicircles governs CYB mRNA editing

**Figure 5.**
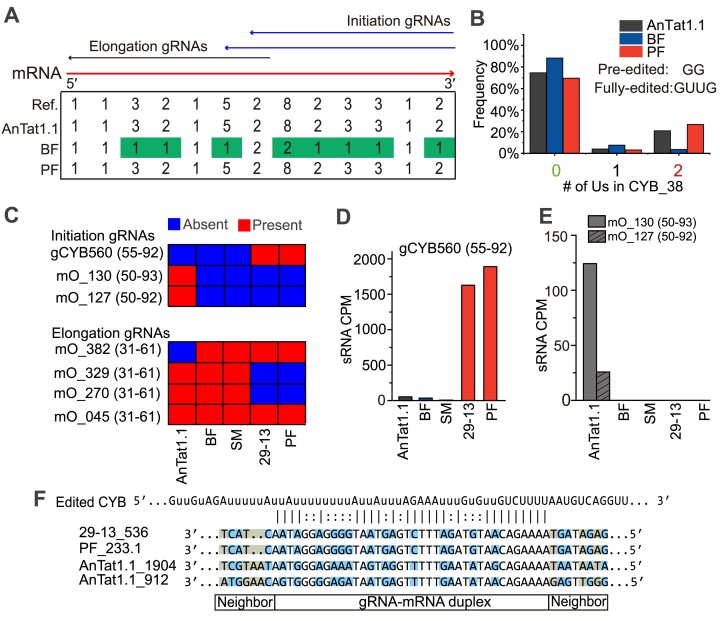
Loss of a minicircle encoding the initiation gRNA for CYB editing in Lister 427 BSF. (**A**) The most frequent length of U-insertions per site across 13 edited sites in CYB mRNA, with two blue and one black arrow indicating the complementary regions of initiation and elongation gRNAs, respectively. Sites where the most abundant U-stretch length differs from the reference [43] are highlighted in green. **(B)** A detailed view of the 3′-most editing sites in CYB on the mRNA. The average frequency from two biological replicates is shown. (**C**) Heatmap highlighting the presence (red) or absence (blue) of gRNAs targeting CYB mRNA. The top three are initiation gRNAs, and the bottom four are elongation gRNAs. (**D**) Bar plots of RNA-level abundances (CPM) for the PF-specific initiation gRNAs. (**E**) Bar plots of RNA-level abundances for the AnTat1.1-specific gRNAs. (**F**) Multiple sequence alignment of initiation gRNAs from PCF cell lines (29-13 and PF) and AnTat1.1. Complementary fully edited CYB mRNA is shown at the top. Identical nucleotides across initiation gRNAs are not colored. Variants tolerated by G-U wobble pairing are colored light blue, and the rest of the variants are colored gray.

The pleomorphic AnTat1.1, maintained as a long slender bloodstream form, exhibited a 100% consistency rate in CYB mRNA editing with the fully edited reference from Lister 427 [34]. In contrast, bloodstream Lister 427 showed only ∼30% consistency (Fig. 4F). Since the moderately edited CYB mRNA undergoes only insertion editing, we examined the number of correct U-stretches at 13 canonical sites in reference strain [43] versus AnTat1.1, PF, and BF cell lines (Fig. 5A and B, and Supplementary Fig. S7A). Notably, inconsistency in editing was predominantly localized to the 3′ end of the editing domain, in line with a previously reported inhibition of editing initiation in Lister 427 BSF [44]. Given that CYB gRNAs lack canonical inverted repeats [45, 46] and might be overlooked by our gRNA prediction pipeline, we compiled eight previously annotated CYB gRNA sequences [5–7, 19]. Analysis of CYB gRNA-encoding minicircles revealed a mosaic distribution among five cell lines, with AnTat1.1 retaining the majority of gRNAs (Fig. 5C). Seven out of eight gRNAs were identified in our minicircle assemblies, and the missing gCYB-558 gRNA [46] may have been lost along with the encoding minicircle. Based on positioning within the editing domain, seven gRNAs were further classified into “initiation” (top three) and “elongation” gRNAs (bottom four) (Fig. 5C). In this context, the lack of initiation gRNA-encoding minicircles in Lister 427 BSF accounts for the inconsistency in editing patterns at the 3′ end of the CYB edited domain (Fig. 5A and B).

To validate the functional roles of these gRNAs, we quantified their abundance in small RNA-seq datasets, confirming consistent expression profiles for the three initiation gRNAs (Fig. 5D and E). The two AnTat1.1-specific initiation gRNAs appear to be missing in four Lister 427 cell lines (Fig. 5E). Conversely, very low (∼1% relative to PCF) expression levels for the CYB-560 gRNA were detected in AnTat1.1 and Lister 427 BSF cell lines (Fig. 5D). Further inspection using the IGV genome browser suggested accurate mapping and a lack of multiple variants (Supplementary Fig. S7B). The nonzero detection of the CYB-560 gRNA could be due to the presence of the CYB-560-encoding minicircle in a subpopulation of cells, consistent with the rapid loss of nonessential minicircles. A close inspection of initiation minicircles revealed substantial sequence variations in the gRNA-coding regions. However, all variations within the predicted gRNA–mRNA duplex involved A-to-G or C-to-U transitions, which were tolerated by the editing machinery through G-U base pairing (Fig. 5F). We hypothesized that incorrect editing in the 3′ region of the CYB editing domain may be mediated by non-cognate gRNAs. Indeed, by using uncorrected CYB mRNA as a query, we identified two gRNAs that may mediate such spurious editing (Supplementary Fig. S7C). Elongation gRNAs also exhibited strain-dependent variations in expression levels (Supplementary Fig. S7D).

Despite overall minicircle genome streamlining in Lister 427, minicircles encoding initiation gRNAs are retained in the procyclic form, which depends on cytochrome oxidase for oxidative phosphorylation, but are lost in bloodstream cell lines that chiefly derive ATP from glycolysis. Consequently, the presence or absence of initiation gRNAs governs the differential editing and likely translation of cytochrome *b*, a critical subunit of respiratory Complex III [47]. Notably, AnTat1.1, maintained as a bloodstream form, has retained an alternative initiation gRNA, thus preserving faithful CYB editing.

### Diverse AnTat1.1 gRNAs promote contiguous editing of the 3′ domain in ND7 mRNA

Although bloodstream parasites rely on glycolysis rather than oxidative phosphorylation for ATP production [11], most Lister 427 mRNAs, except CYB and CO2, are edited more extensively in BSF than in PCF, with AnTat1.1 showing intermediate levels (Fig. 4B). To assess whether gRNA abundance and diversity influence editing efficiency, we compared Lister 427 BF and AnTat1.1 to reveal strain-specific editing characteristics.

**Figure 6.**
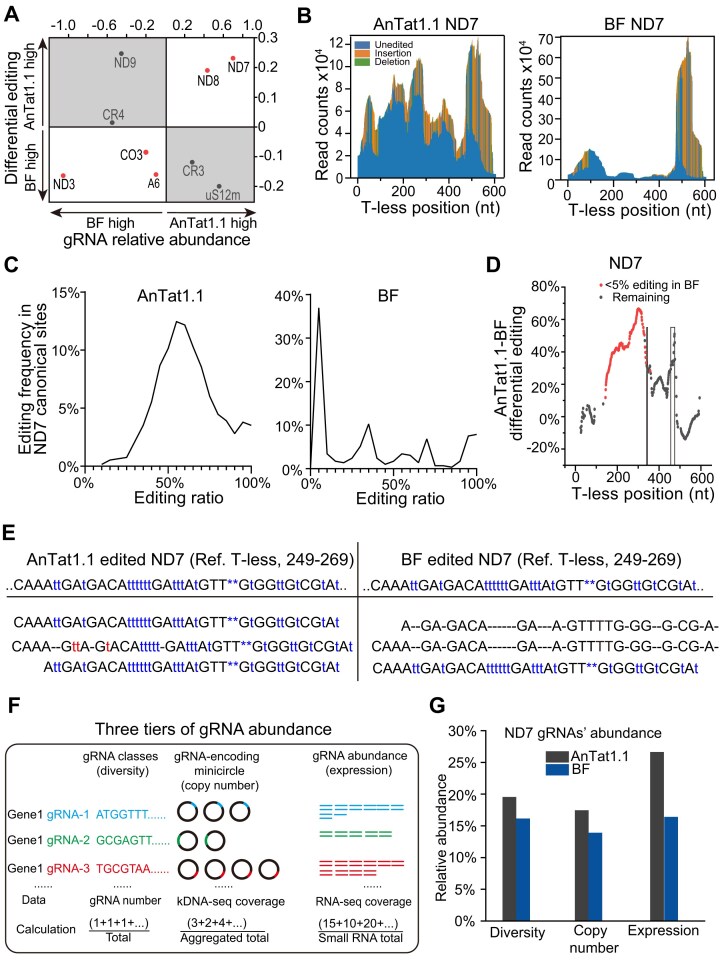
Association of gRNA diversity and relative abundance with mRNA editing. (**A**) Scatter plot showing the association between strain-specific differences in editing levels and the corresponding gRNA abundances. The *x*-axis represents the log_2_ CPM ratios of mRNA-specific gRNA abundances, while the *y*-axis shows the median mRNA editing level differences between the AnTat1.1 and BF strains. Red dots indicate a concordant increase in both mRNA and gRNA abundances. (**B**) Positional mapping of editing events in ND7 mRNA for AnTat1.1 and BF. The *x*-axis shows T-less mRNA coordinates. The *y*-axis indicates read depth for pre-edited (blue), U-insertion (orange), and U-deletion (green) states at each position. (**C**) Frequency distribution of editing levels for canonical editing sites in ND7 from AnTat1.1 and BF. (**D**) Per-site differential editing levels in ND7 mRNA between AnTat1.1 and BF. Sites with BF editing ratios lower than 5% are colored red. Regions (T-less positions 340 and 467–476) with incomplete gRNA coverage in BF are marked by rectangles. (**E**) Examples of ND7 transcripts from AnTat1.1 (left) and BF (right). With respect to reference fully edited transcripts, concordant insertions are denoted as blue “t” and deletions as blue asterisks, and mis-insertions are denoted as red “t”. (**F**) Diagram illustrating three tiers of gRNA abundance: (i) gRNA sequence diversity; (ii) copy number of encoding minicircles; and (iii) steady-state gRNA abundance. The relative abundances are normalized to the total in each category. (**G**) Comparison of ND7-specific gRNA diversity, copy number, and expression level between AnTat1.1 (black) and BF.

To evaluate global gRNA abundance per transcript, we summed all gRNAs targeting each mRNA. Since elevated editing of ND7, ND8, A6, ND3, and CO3 mRNAs paralleled their strain-specific gRNA levels (Fig. 6A), we focused on ND7 as the transcript showing the largest increase in both gRNA abundance and editing in AnTat1.1 compared to Lister 427 BF. Earlier work in Lister 427 documented ND7 pan-editing as two discrete domains, with the 3′ domain edited only in bloodstream cells [7, 48]. Overall, our mRNA-seq analysis of Lister 427 bloodstream cell line confirmed this observation although most editing sites in the 3′ domain (∼130–400 T-less positions) were either unedited or edited at low levels (Fig. 6B and Supplementary Fig. S8A), contributing to the sharp peak at 0% that is uniquely present in BF but not in AnTat1.1 (Fig. 6C). In addition, single-site resolution analyses (Fig. 6D) and representative mRNA-seq reads (Fig. 6E) confirmed higher editing levels in the central region within 3′ editing domain in AnTat1.1 compared with Lister 427 BF.

To further clarify the origin of the more complete editing patterns in the 3′ domain of ND7 in AnTat1.1, we derived a three-tier assessment of the gRNA pool. For each transcript in each cell line, we calculated (i) gRNA diversity as the percentage of specific mRNA-targeting gRNAs of the total gRNA repertoire; (ii) kDNA-seq-based abundance, representing the aggregated copy number of gRNA genes; and (iii) RNA-seq-based abundance, representing the aggregated steady-state gRNA levels (Fig. 6F). We distinguished copy numbers and expression levels because previous studies have shown that gRNA expression does not always correlate with minicircle abundance [49]. Nevertheless, we observed a statistically significant positive correlation between gene copy number and expression levels across all five cell lines (Supplementary Fig. S8B). The enrichment of ND7-targeting gRNAs in AnTat1.1 versus BF was evident at all three tiers (Fig. 6G). An inverse trend was observed for A6 mRNA-specific gRNAs, expressed at higher levels in BF than in AnTat1.1 (Supplementary Fig. S8C and D). These results suggest that both gRNA diversity and abundance contribute to differential mitochondrial mRNA editing in laboratory cell lines of *T. brucei*.

### Longer gRNA–mRNA hybrids contribute to more efficient editing

**Figure 7.**
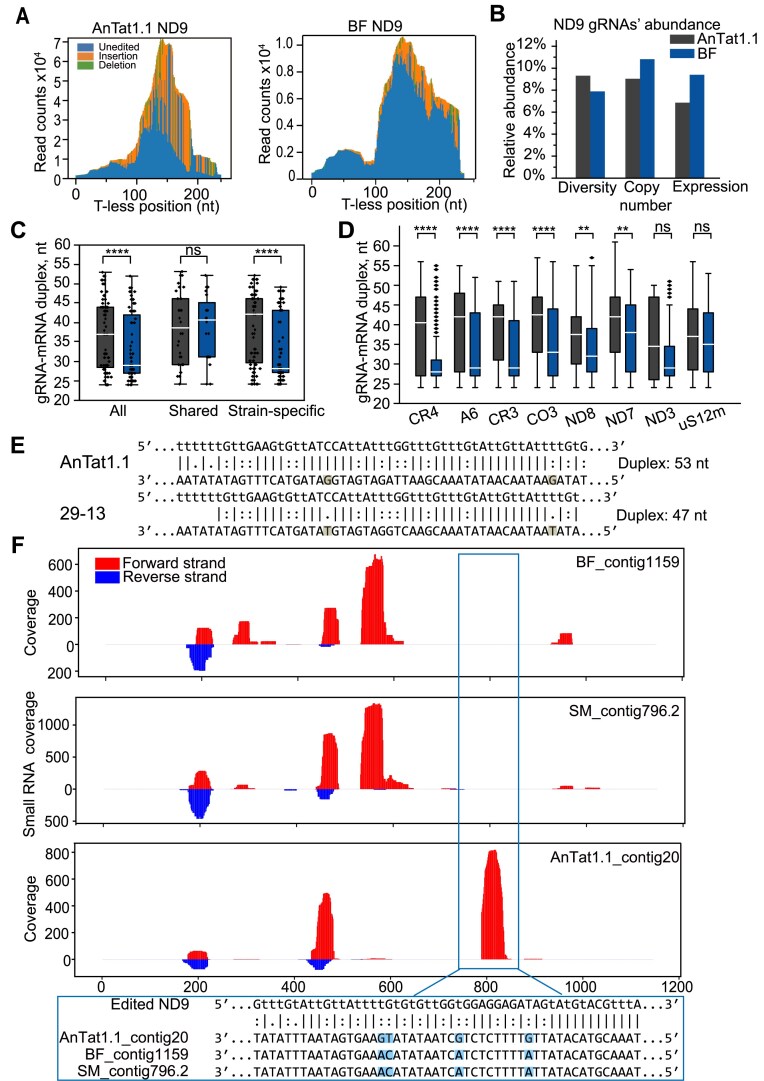
Variability in predicted RNA–mRNA duplex lengths across different strains. (**A**) Positional mapping of editing events in ND9 mRNA for AnTat1.1 and Lister 427 strains. The *x*-axis represents the 5′ to 3′ coordinates of the T-less mRNA. The *y*-axis shows the total read depth for pre-edited (blue), U-insertion (orange), and U-deletion (green) states of each position. (**B**) Three layers of gRNA abundance (diversity, copy number, and expression) for ND9 in AnTat1.1 (black) and BF (blue). (**C**) Boxplots of gRNA–mRNA duplex lengths for ND9 gRNAs shared between AnTat1.1 and Lister 427, and strain-specific gRNAs. Statistical significance was evaluated by the Mann–Whitney–Wilcoxon test. (**D**) Boxplots of duplex lengths for gRNAs targeting other mRNAs in AnTat1.1 and Lister 427 strains. Statistical significance was evaluated by the Mann–Whitney–Wilcoxon test. Multiple hypothesis testing adjustment was performed by using the Benjamini–Hochberg procedure, and adjusted *P*-values were reported. (**E**) A decreased ND9 gRNA–mRNA duplex length caused by non-A-to-G and non-C-to-T mutations in BF versus AnTat1.1. (**F**) Example of strain-specific differences in ND9-specific gRNA expression. Small RNA-seq reads from AnTat1.1 and two Lister 427 BSF cell lines (BF and SM) were mapped to minicircles. The ND9 gRNA region (blue rectangle) shows high expression (coverage) in AnTat1.1 and lack of coverage in BF and SM. The gRNA sequences are shown below. G-U wobble-tolerant variants are highlighted in light blue. **** represents *P*  < .0001; ** represents *P*  < .01; * represents *P* < .05; ns (not significant) represents *P* ≥ .05.

ND9 mRNA also exhibited the highest editing across the entire transcript in AnTat1.1 (Figs 6A and 7A, and Supplementary Fig. S9A), yet the steady-state levels of gRNAs targeting ND9 were similar between AnTat1.1 and Lister 427 (Fig. 7B). Therefore, we next evaluated the completeness of gRNA coverage for ND9 in BF compared with AnTat1.1 but observed only a slightly higher coverage in AnTat1.1 (Supplementary Fig. S9B and Supplementary Table S7). The incompletely covered insertions in BF were located at T-less positions 15–22, 43, and 236. However, editing levels at positions 15–22 and 43 were low in both AnTat1.1 and BF, and the single site at position 236 is unlikely to account for the overall increase in ND9 editing in AnTat1.1.

Since differences in gRNA abundance and coverage could not explain elevated ND9 editing, we explored whether gRNAs in AnTat1.1 are more efficient. Analysis of predicted gRNA–edited mRNA duplexes revealed significantly longer complementary regions in AnTat1.1 (∼37 bp) compared to monomorphic cell lines (∼29 bp) (Fig. 7C). Consistent with previous findings that artificially stabilized gRNA–mRNA hybrids enhance RNA editing activity *in vitro* [50–52], gRNAs capable of forming more extensive base-pairing with mRNA appear to exhibit greater efficiency *in vivo*.

To begin elucidating the cause of extended gRNA–mRNA hybrids in AnTat1.1, we examined the length of gRNA–mRNA complementary regions potentially formed by gRNAs common to and distinct between AnTat1.1 and BF. This analysis showed that gRNAs originating from minicircles shared by AnTat1.1 and BF generally form duplexes of similar length, whereas AnTat1.1-specific gRNAs align with their mRNA targets as significantly longer hybrids (Fig. 7C and Supplementary Fig. S9C and D). These findings suggest that gRNAs encoded in minicircles unique to AnTat1.1 generally form longer gRNA–mRNA hybrids, a correlation observed for six out of eight pan-edited mRNAs in AnTat1.1, in addition to ND9 (Fig. 7D).

To further investigate this pattern, we analyzed minicircles shared between AnTat1.1 and Lister 427 strains. Most of these minicircles encode gRNAs that form duplexes of equal length; however, two groups showed differences. Specifically, gRNA–mRNA duplexes were shorter in Lister 427 due to mutations in gRNA genes (Fig. 7E), or gRNAs were not expressed in Lister 427 BSF despite potentially being capable of forming duplexes of equal length (Fig. 7F and Supplementary Fig. S9E). Previous studies on procyclic and bloodstream forms of the EATRO 164 strain also showed that gRNAs from the former exhibited a longer duplex length than those from the latter [6]. We also identified the few cases where longer duplex-forming gRNAs conserved between procyclic and bloodstream forms of EATRO 164 are also present in minicircles from our Lister 427 and AnTat1.1 cell lines (Supplementary Fig. S9F). This finding suggests that the examples of longer duplexes retained by different strains reflect those of ancestral isolates.

Overall, the correlative gRNA/mRNA analysis suggests that longer gRNA–mRNA duplexes are associated with more efficient editing. It seems plausible that, over time, accumulated mutations in gRNA genes under relaxed selective pressure may have shortened these duplexes or silenced their expression.

## Discussion

We performed a comparative analysis of maxicircle and minicircle genomes and their transcriptomes from developmentally competent polymorphic AnTat1.1 and bloodstream and procyclic monomorphic Lister 427 cell lines of *T. brucei*. Studies by Cooper *et al.* comprehensively characterized the maxicircles, minicircles, and small RNA transcriptome in AnTat1.1 [5, 19]. Here, we expanded this approach by including mRNA data to further explore mitochondrial genome divergence and potential relationships among encoded gRNA repertoires, their abundance and editing efficiency, and the resultant mRNA editing patterns in the widely utilized laboratory cell lines derived from *T. brucei* Lister 427 strain.

Maxicircles were nearly identical among the four Lister 427 cell lines, whereas the AnTat1.1’s maxicircle harbored numerous silent and nonsynonymous substitutions (Table 3). In Lister 427, shortened maxicircle-encoded U-stretches eliminated the requirement for deletion RNA editing at two positions (167 in ND3 and 415 in ND7; Fig. 1E–H), a modification absent in AnTat1.1. Overall, maxicircles, which are present in a few copies per kDNA network, remain stable irrespective of cultivation conditions or developmental forms, likely because any losses may adversely affect the expression levels of mitochondrially encoded proteins.

We confirmed that AnTat1.1 minicircles cluster into ∼400 sequence classes and characterized a substantially reduced diversity of minicircles (∼200 sequence classes) in Lister 427 cell lines (Fig. 2D). We observed significant variations in the minicircle repertoire—and thus encoded gRNAs and resulting editing patterns—among cell lines derived from the same strain but maintained as either bloodstream or procyclic forms (Fig. 2D).

Cultivation history-dependent streamlining of minicircle genome is established for the related kinetoplastid parasite, *L. tarentolae* [16, 17, 42], as well as trypanosomatid *Vickermania* [53], and presumably results from prolonged exposure to nutrient-rich axenic conditions. Since minicircles are believed to be randomly distributed during kDNA replication and segregation [54], selective pressures likely retain only those encoding gRNAs required for editing of mRNAs essential under particular conditions. Consistently, the dispensability of Complex I in procyclic *T. brucei* cells [13] aligns with the loss of minicircles encoding gRNAs necessary for ND7 mRNA editing. It appears that during successive passages, selection favoring rapidly proliferating parasites and the relaxation of pressure to maintain minicircles, which are unnecessary only under certain conditions, gradually decreased minicircle diversity.

Our findings support a model of maxicircle–minicircle coevolution, whereby the loss of editing requirements—either through maxicircle mutations or reduced environmental selection—leads to the disappearance of corresponding gRNA-carrying minicircles (Fig. 2D). Consequently, minicircle sequence diversity decreased by half from AnTat1.1 to Lister 427. Additionally, our data indicate that maxicircle sequence diversification mainly occurred through G-to-U tolerant single-nucleotide substitutions (A-to-G and C-to-U; Fig. 1C and Supplementary Table S2) and variations in U-stretch lengths (Fig. 1D–I, Supplementary Fig. S3G–I, and Supplementary Table S2). Selection pressure favoring G-to-U tolerant mutations was also evident in the gRNA–mRNA duplex regions (Figs 5F and 7E). Similar mutations were reported in various strains of *Trypanosoma lewisi* [55]. These findings underscore the merit of potential long-term in-culture evolution experiments to quantify the rates of minicircle extinction.

Despite substantial minicircle genome divergence, sequences of fully edited mRNAs remained largely consistent across strains. Editing specificity is reinforced by two factors: noncanonical editing sites remain largely unedited (Fig. 4A), and at canonical sites, faithful U-insertions dominate (Fig. 4E–F). Although AnTat1.1 encodes diverse gRNAs, we detected no editing in never-edited mRNAs (Fig. 3). Given the abundance of partially edited mitochondrial transcripts [41], it appears that editing specificity is primarily dictated at the initial gRNA–mRNA recognition step [33], rather than the ensuing cascade of editing reactions [8]. Our findings do not exclude the possibility of alternative editing products, but suggest that their impact on the mitochondrial proteome is negligible due to their low abundance relative to canonical counterparts. Reliable proteomic analysis of unusually hydrophobic mitochondrial proteins remains challenging, complicating the identification of potential protein products of alternatively edited mRNAs.

Although sequences of fully edited mRNAs are nearly uniform, editing efficiencies differ significantly across transcripts and cell lines. Previous studies indicated that differential editing is unrelated to gRNA availability among monomorphic cell lines under various axenic conditions [6]. In contrast, our data suggest that strain-specific minicircle repertoires, particularly the presence or absence of initiation gRNAs, are crucial for CYB mRNA editing (Figs 4B and 5B). Therefore, both gRNA availability and steady-state levels likely regulate stage-specific efficiency of RNA editing. The detection of trace initiation gRNAs in Lister 427 BSF lines (Fig. 5D and Supplementary Fig. S7B) supports their loss under relaxed selective pressure during cultivation. This aligns with earlier findings identifying initiation gRNAs as key determinants in CYB editing progression [56].

High editing levels correlate with increased gRNA diversity and abundance, influenced by the minicircle genome composition. Consistent with previous findings [6], greater gRNA class numbers and higher gRNA abundances targeting A6, CO3, ND3, ND7, and ND8 mRNAs positively correlated with more efficient editing in AnTat1.1 and BSF strains (Fig. 6 and Supplementary Fig. S8). Furthermore, predicted gRNA–mRNA hybrids were longer in AnTat1.1 than in monomorphic cell lines (Fig. 7 and Supplementary Fig. S9). Shortened hybrid regions, likely due to accumulated gRNA gene mutations (Fig. 7E), have been documented for ND8 mRNA [6]. Similar hybrid length reduction occurred from dixenous *L. tarentolae* to monoxenous *Leptomonas pyrrhocoris* [57]. In this case, the monoxenous species likely evolved from a dixenous ancestor [58–60]. Our observations in *T. brucei* mirror these evolutionary patterns: monomorphic Lister 427 strains exhibit reduced gRNA–mRNA duplex lengths relative to AnTat1.1.

Minicircle presence and sequence dictate gRNA availability and potential gRNA–mRNA hybrid lengths, while steady-state gRNA levels depend on minicircle copy numbers and nature of inverted repeats flanking gRNA genes [5, 61]. However, these factors alone do not explain all examples of differential editing between strains. Despite higher relative gRNA abundance and gRNA–mRNA duplex lengths for CR3 and uS12m in AnTat1.1, editing efficiency remains lower compared to Lister 427 (Figs 6A and 7D, and Supplementary Table S7). Additional regulatory mechanisms influencing gRNA maturation, decay, stabilization, and utilization likely contribute to this complexity [33, 61–63], alongside interactions with non-cognate gRNAs mediated by KREH2 helicase [64, 65] and variability in RNA editing catalytic complexes [66, 67].

Differences between AnTat1.1 and Lister 427 likely arise from their distinct genetic backgrounds and cultivation histories, and quantitative assessment of these factors remains a future endeavor. Nonetheless, investigating the kinetoplast genome and transcriptome variations related to developmental potential and cultivation history provides valuable insights into mitochondrial genome streamlining and adaptations in the RNA editing machinery.

## Supplementary Material

gkaf661_Supplemental_Files

## Data Availability

Sequencing data were deposited to NCBI Sequence Read Archive (SRA) under the BioProject accession PRJNA1183446 (kDNA-seq), PRJNA1184820 (mRNA-seq), PRJNA1184957 (sRNA-seq replicate 1), and PRJNA1185305 (sRNA-seq replicate 2). Custom scripts related to this study can be found at https://gitee.com/Zhanglab/minicircle_maxcircle_strain_cmp/. Maxicircle and minicircle assemblies as well as canonical mRNA sequences were also deposited at the Gitee repository. The maxicircle assemblies can be found at https://gitee.com/Zhanglab/minicircle_maxcircle_strain_cmp/tree/master/data-deposit/maxcircle. The minicircle assemblies can be found at https://gitee.com/Zhanglab/minicircle_maxcircle_strain_cmp/tree/master/data-deposit/minicircle. The canonical mRNA sequence can be found at https://gitee.com/Zhanglab/minicircle_maxcircle_strain_cmp/tree/master/data-deposit/canonical%20mRNA%20sequences.

## Notes

Present address: Bioinformatics and Genomics PhD Program, Pennsylvania State University College of Medicine, Hershey, PA 16802, United States

## References

[1] Lukes J, Guilbride DL, Votypka J et al. Kinetoplast DNA network: evolution of an improbable structure. Eukaryot Cell. 2002; 1:495–502. 10.1128/EC.1.4.495-502.2002. 12455998 PMC117999

[2] Jensen RE, Englund PT Network news: the replication of kinetoplast DNA. Annu Rev Microbiol. 2012; 66:473–91. 10.1146/annurev-micro-092611-150057. 22994497

[3] Simpson L, Neckelmann N, de la Cruz V et al. Comparison of the maxicircle (mitochondrial) genomes of *Leishmania tarentolae* and *Trypanosoma brucei* at the level of nucleotide sequence. J Biol Chem. 1987; 262:6182–96. 10.1016/S0021-9258(18)45555-X. 3032958

[4] Blum B, Bakalara N, Simpson L A model for RNA editing in kinetoplastid mitochondria: “guide” RNA molecules transcribed from maxicircle DNA provide the edited information. Cell. 1990; 60:189–98. 10.1016/0092-8674(90)90735-W. 1688737

[5] Cooper S, Wadsworth ES, Ochsenreiter T et al. Assembly and annotation of the mitochondrial minicircle genome of a differentiation-competent strain of *Trypanosoma brucei*. Nucleic Acids Res. 2019; 47:11304–25. 10.1093/nar/gkz928. 31665448 PMC6868439

[6] Kirby LE, Sun Y, Judah D et al. Analysis of the *Trypanosoma brucei* EATRO 164 bloodstream guide RNA transcriptome. PLoS Negl Trop Dis. 2016; 10:e0004793 10.1371/journal.pntd.0004793. 27399202 PMC4939953

[7] Koslowsky D, Sun Y, Hindenach J et al. The insect-phase gRNA transcriptome in *Trypanosoma brucei*. Nucleic Acids Res. 2014; 42:1873–86. 10.1093/nar/gkt973. 24174546 PMC3919587

[8] Aphasizheva I, Alfonzo J, Carnes J et al. Lexis and grammar of mitochondrial RNA processing in trypanosomes. Trends Parasitol. 2020; 36:337–55. 10.1016/j.pt.2020.01.006. 32191849 PMC7083771

[9] Maslov DA, Avila HA, Lake JA et al. Evolution of RNA editing in kinetoplastid protozoa. Nature. 1994; 368:345–8. 10.1038/368345a0. 8127370

[10] Pyrih J, Hammond M, Alves A et al. Comprehensive sub-mitochondrial protein map of the parasitic protist *Trypanosoma brucei* defines critical features of organellar biology. Cell Rep. 2023; 42:113083 10.1016/j.celrep.2023.113083. 37669165

[11] Schnaufer A, Clark-Walker GD, Steinberg AG et al. The F_1_-ATP synthase complex in bloodstream stage trypanosomes has an unusual and essential function. EMBO J. 2005; 24:4029–40. 10.1038/sj.emboj.7600862. 16270030 PMC1356303

[12] Aphasizheva I, Maslov DA, Aphasizhev R Kinetoplast DNA-encoded ribosomal protein S12: a possible functional link between mitochondrial RNA editing and translation in *Trypanosoma brucei*. RNA Biol. 2013; 10:1679–88. 10.4161/rna.26733. 24270388 PMC3907478

[13] Verner Z, Cermakova P, Skodova I et al. Complex I (NADH:ubiquinone oxidoreductase) is active in but non-essential for procyclic *Trypanosoma brucei*. Mol Biochem Parasitol. 2011; 175:196–200. 10.1016/j.molbiopara.2010.11.003. 21074578

[14] Surve S, Heestand M, Panicucci B et al. Enigmatic presence of mitochondrial complex I in *Trypanosoma brucei* bloodstream forms. Eukaryot Cell. 2012; 11:183–93. 10.1128/EC.05282-11. 22158713 PMC3272898

[15] Roy J, Faktorova D, Lukes J et al. Unusual mitochondrial genome structures throughout the Euglenozoa. Protist. 2007; 158:385–96. 10.1016/j.protis.2007.03.002. 17499547

[16] Thiemann OH, Maslov DA, Simpson L Disruption of RNA editing in *Leishmania tarentolae* by the loss of minicircle-encoded guide RNA genes. EMBO J. 1994; 13:5689–700. 10.1002/j.1460-2075.1994.tb06907.x. 7988566 PMC395534

[17] Simpson L, Douglass SM, Lake JA et al. Comparison of the mitochondrial genomes and steady state transcriptomes of two strains of the trypanosomatid parasite,*Leishmania tarentolae*. PLoS Negl Trop Dis. 2015; 9:e0003841 10.1371/journal.pntd.0003841. 26204118 PMC4512693

[18] Wirtz E, Leal S, Ochatt C et al. A tightly regulated inducible expression system for conditional gene knock-outs and dominant-negative genetics in *Trypanosoma brucei*. Mol Biochem Parasitol. 1999; 99:89–101. 10.1016/S0166-6851(99)00002-X. 10215027

[19] Cooper S, Wadsworth ES, Schnaufer A et al. Organization of minicircle cassettes and guide RNA genes in *Trypanosoma brucei*. RNA. 2022; 28:972–92. 35414587 10.1261/rna.079022.121 PMC9202587

[20] Smith JT Jr, Tylec B, Naguleswaran A et al. Developmental dynamics of mitochondrial mRNA abundance and editing reveal roles for temperature and the differentiation-repressive kinase RDK1 in cytochrome oxidase subunit II mRNA editing. mBio. 2023; 14:e0185423 10.1128/mbio.01854-23. 37795988 PMC10653865

[21] Sbicego S, Vassella E, Kurath U et al. The use of transgenic *Trypanosoma brucei* to identify compounds inducing the differentiation of bloodstream forms to procyclic forms. Mol Biochem Parasitol. 1999; 104:311–22. 10.1016/S0166-6851(99)00157-7. 10593184

[22] Aphasizheva I, Zhang L, Aphasizhev R Investigating RNA editing factors from trypanosome mitochondria. Methods. 2016; 107:23–33. 10.1016/j.ymeth.2016.03.020. 27020893 PMC5094665

[23] Aslett M, Aurrecoechea C, Berriman M et al. TriTrypDB: a functional genomic resource for the Trypanosomatidae. Nucleic Acids Res. 2010; 38:D457–62. 10.1093/nar/gkp851. 19843604 PMC2808979

[24] Md V, Misra S, Li H et al. IEEE International Parallel and Distributed Processing Symposium (IPDPS). 2019; Rio de Janeiro, Brazil.

[25] Danecek P, Bonfield JK, Liddle J et al. Twelve years of SAMtools and BCFtools. GigaScience. 2021; 10:giab008. 33590861 10.1093/gigascience/giab008 PMC7931819

[26] McKenna A, Hanna M, Banks E et al. The Genome Analysis Toolkit: a MapReduce framework for analyzing next-generation DNA sequencing data. Genome Res. 2010; 20:1297–303. 10.1101/gr.107524.110. 20644199 PMC2928508

[27] Chen S, Zhou Y, Chen Y et al. fastp: an ultra-fast all-in-one FASTQ preprocessor. Bioinformatics. 2018; 34:i884–90. 10.1093/bioinformatics/bty560. 30423086 PMC6129281

[28] Van Den Broeck F, Savill NJ, Imamura H et al. Ecological divergence and hybridization of Neotropical *Leishmania* parasites. Proc Natl Acad Sci USA. 2020; 117:25159–68. 10.1073/pnas.1920136117. 32958676 PMC7547230

[29] Dierckxsens N, Mardulyn P, Smits G NOVOPlasty: *de novo* assembly of organelle genomes from whole genome data. Nucleic Acids Res. 2017; 45:e18. 28204566 10.1093/nar/gkw955 PMC5389512

[30] Kurtz S, Phillippy A, Delcher AL et al. Versatile and open software for comparing large genomes. Genome Biol. 2004; 5:R12 10.1186/gb-2004-5-2-r12. 14759262 PMC395750

[31] Li W, Godzik A Cd-hit: a fast program for clustering and comparing large sets of protein or nucleotide sequences. Bioinformatics. 2006; 22:1658–9. 10.1093/bioinformatics/btl158. 16731699

[32] Trapnell C, Williams BA, Pertea G et al. Transcript assembly and quantification by RNA-Seq reveals unannotated transcripts and isoform switching during cell differentiation. Nat Biotechnol. 2010; 28:511–5. 10.1038/nbt.1621. 20436464 PMC3146043

[33] Liu S, Wang H, Li X et al. Structural basis of gRNA stabilization and mRNA recognition in trypanosomal RNA editing. Science. 2023; 381:eadg4725 10.1126/science.adg4725. 37410820 PMC10704856

[34] Ochsenreiter T, Cipriano M, Hajduk SL KISS: the kinetoplastid RNA editing sequence search tool. RNA. 2007; 13:1–4. 10.1261/rna.232907. 17123956 PMC1705751

[35] Geerts M, Schnaufer A, Van den Broeck F rKOMICS: an R package for processing mitochondrial minicircle assemblies in population-scale genome projects. BMC Bioinformatics. 2021; 22:468 10.1186/s12859-021-04384-1. 34583651 PMC8479924

[36] Gerasimov ES, Zamyatnina KA, Matveeva NS et al. Common structural patterns in the maxicircle divergent region of Trypanosomatidae. Pathogens. 2020; 9:100 10.3390/pathogens9020100. 32033466 PMC7169413

[37] Kaufer A, Stark D, Ellis J A review of the systematics, species identification and diagnostics of the Trypanosomatidae using the maxicircle kinetoplast DNA: from past to present. Int J Parasitol. 2020; 50:449–60. 10.1016/j.ijpara.2020.03.003. 32333942

[38] Souza AE, Myler PJ, Stuart K Maxicircle CR1 transcripts of *Trypanosoma brucei* are edited, developmentally regulated, and encode a putative iron–sulfur protein homologous to an NADH dehydrogenase subunit. Mol Cell Biol. 1992; 12:2100–7. 1373807 10.1128/mcb.12.5.2100-2107.1992 PMC364381

[39] Gerasimov ES, Novozhilova TS, Zimmer SL et al. Kinetoplast genome of *Leishmania* spp. is under strong purifying selection. Trop Med Infect Dis. 2023; 8:384. 37624322 10.3390/tropicalmed8080384 PMC10458658

[40] Hong M, Simpson L Genomic organization of *Trypanosoma brucei* kinetoplast DNA minicircles. Protist. 2003; 154:265–79. 10.1078/143446103322166554. 13677453

[41] Gerasimov ES, Gasparyan AA, Kaurov I et al. Trypanosomatid mitochondrial RNA editing: dramatically complex transcript repertoires revealed with a dedicated mapping tool. Nucleic Acids Res. 2018; 46:765–81. 10.1093/nar/gkx1202. 29220521 PMC5778460

[42] Gao GH, Kapushoc ST, Simpson AM et al. Guide RNAs of the recently isolated LEM125 strain of *Leishmania tarentolae*: an unexpected complexity. RNA. 2001; 7:1335–47. 10.1017/S1355838201018076. 11565754 PMC1370176

[43] Feagin J, Jasmer D, Stuart K Developmentally regulated addition of nucleotides within apocytochrome *b* transcripts in *Trypanosoma brucei*. Cell. 1987; 49:337–45. 10.1016/0092-8674(87)90286-8. 3568129

[44] Smith JT Jr, Doleželová E, Tylec B et al. Developmental regulation of edited CYb and COIII mitochondrial mRNAs is achieved by distinct mechanisms in *Trypanosoma brucei*. Nucleic Acids Res. 2020; 48:8704–23. 10.1093/nar/gkaa641. 32738044 PMC7470970

[45] Feagin J, Jasmer D, Stuart K Apocytochrome *b* and other mitochondrial DNA sequences are differentially expressed during the life cycle of *Trypanosoma brucei*. Nucleic Acids Res. 1985; 13:4577–96. 10.1093/nar/13.12.4577. 2409537 PMC321807

[46] Riley GR, Corell RA, Stuart K Multiple guide RNAs for identical editing of *Trypanosoma brucei* apocytochrome *b* mRNA have an unusual minicircle location and are developmentally regulated. J Biol Chem. 1994; 269:6101–8. 10.1016/S0021-9258(17)37575-0. 7509798

[47] Gnipova A, Panicucci B, Paris Z et al. Disparate phenotypic effects from the knockdown of various *Trypanosoma brucei* cytochrome *c* oxidase subunits. Mol Biochem Parasitol. 2012; 184:90–8. 10.1016/j.molbiopara.2012.04.013. 22569586

[48] Koslowsky DJ, Bhat GJ, Perrollaz AL et al. The MURF3 gene of *T. brucei* contains multiple domains of extensive editing and is homologous to a subunit of NADH dehydrogenase. Cell. 1990; 62:901–11. 10.1016/0092-8674(90)90265-G. 2393904

[49] Maslov DA, Simpson L The polarity of editing within a multiple gRNA-mediated domain is due to formation of anchors for upstream gRNAs by downstream editing. Cell. 1992; 70:459–67. 10.1016/0092-8674(92)90170-H. 1379519

[50] Igo RP, Lawson SD, Stuart K RNA sequence and base pairing effects on insertion editing in *Trypanosoma brucei*. Mol Cell Biol. 2002; 22:1567–76. 10.1128/MCB.22.5.1567-1576.2002. 11839822 PMC134691

[51] Igo RP, Palazzo SS, Burgess ML et al. Uridylate addition and RNA ligation contribute to the specificity of kinetoplastid insertion RNA editing. Mol Cell Biol. 2000; 20:8447–57. 10.1128/MCB.20.22.8447-8457.2000. 11046141 PMC102151

[52] Cruz-Reyes J, Zhelonkina A, Rusche L et al. Trypanosome RNA editing: simple guide RNA features enhance U deletion 100-fold. Mol Cell Biol. 2001; 21:884–92. 10.1128/MCB.21.3.884-892.2001. 11154275 PMC86679

[53] Gerasimov ES, Afonin DA, Škodová-Sveráková I et al. Evolutionary divergent kinetoplast genome structure and RNA editing patterns in the trypanosomatid *Vickermania*. Proc Natl Acad Sci USA. 2025; 122:e2426887122 10.1073/pnas.2426887122. 40203041 PMC12012515

[54] Amodeo S, Bregy I, Ochsenreiter T Mitochondrial genome maintenance—the kinetoplast story. FEMS Microbiol Rev. 2023; 47:fuac047 10.1093/femsre/fuac047. 36449697 PMC10719067

[55] Li SJ, Zhang X, Lukeš J et al. Novel organization of mitochondrial minicircles and guide RNAs in the zoonotic pathogen *Trypanosoma lewisi*. Nucleic Acids Res. 2020; 48:9747–61. 10.1093/nar/gkaa700. 32853372 PMC7515712

[56] Tylec BL, Simpson RM, Kirby LE et al. Intrinsic and regulated properties of minimally edited trypanosome mRNAs. Nucleic Acids Res. 2019; 47:3640–57. 10.1093/nar/gkz012. 30698753 PMC6468165

[57] Gerasimov ES, Gasparyan AA, Afonin DA et al. Complete minicircle genome of *Leptomonas pyrrhocoris* reveals sources of its non-canonical mitochondrial RNA editing events. Nucleic Acids Res. 2021; 49:3354–70. 10.1093/nar/gkab114. 33660779 PMC8034629

[58] Kaufer A, Ellis J, Stark D et al. The evolution of trypanosomatid taxonomy. Parasit Vectors. 2017; 10:287 10.1186/s13071-017-2204-7. 28595622 PMC5463341

[59] Votýpka J, d’Avila-Levy CM, Grellier P et al. New approaches to systematics of Trypanosomatidae: criteria for taxonomic (re)description. Trends Parasitol. 2015; 31:460–9. 10.1016/j.pt.2015.06.015. 26433249

[60] Oldrieve G, Verney M, Jaron KS et al. Monomorphic *Trypanozoon*: towards reconciling phylogeny and pathologies. Microb Genom. 2021; 7:000632. 34397347 10.1099/mgen.0.000632 PMC8549356

[61] Suematsu T, Zhang L, Aphasizheva I et al. Antisense transcripts delimit exonucleolytic activity of the mitochondrial 3′ processome to generate guide RNAs. Mol Cell. 2016; 61:364–78. 10.1016/j.molcel.2016.01.004. 26833087 PMC4744118

[62] Aphasizheva I, Zhang L, Wang X et al. RNA binding and core complexes constitute the U-insertion/deletion editosome. Mol Cell Biol. 2014; 34:4329–42. 10.1128/MCB.01075-14. 25225332 PMC4248751

[63] Weng J, Aphasizheva I, Etheridge RD et al. Guide RNA-binding complex from mitochondria of trypanosomatids. Mol Cell. 2008; 32:198–209. 10.1016/j.molcel.2008.08.023. 18951088 PMC2645705

[64] Meehan J, Ivens A, Grote S et al. KREH2 helicase represses ND7 mRNA editing in procyclic-stage *Trypanosoma brucei* by opposite modulation of canonical and ‘moonlighting’ gRNA utilization creating a proposed mRNA structure. Nucleic Acids Res. 2024; 52:11940–59. 10.1093/nar/gkae699. 39149912 PMC11514453

[65] Meehan J, McDermott SM, Ivens A et al. Trypanosome RNA helicase KREH2 differentially controls non-canonical editing and putative repressive structure via a novel proposed ‘bifunctional’ gRNA in mRNA A6. Nucleic Acids Res. 2023; 51:6944–65. 10.1093/nar/gkad453. 37246647 PMC10359474

[66] McDermott SM, Carnes J, Stuart K Identification by random mutagenesis of functional domains in KREPB5 that differentially affect RNA editing between life cycle stages of *Trypanosoma brucei*. Mol Cell Biol. 2015; 35:3945–61. 10.1128/MCB.00790-15. 26370513 PMC4628071

[67] McDermott SM, Guo X, Carnes J et al. Differential editosome protein function between life cycle stages of *Trypanosoma brucei*. J Biol Chem. 2015; 290:24914–31. 10.1074/jbc.M115.669432. 26304125 PMC4599000

