# Supplementary material for: Comparative mitochondrial genome and transcriptome analyses reveal strain-specific features of RNA editing in Trypanosoma brucei
Source: Nucleic Acids Res. 2025 Jul 16;53(13):gkaf661. doi: 10.1093/nar/gkaf661 (PMC12266143; doi:10.1093/nar/gkaf661)
Supplement: gkaf661_Supplemental_Files [file gkaf661_supplemental_files.zip › Zhao-Sup-R2.pdf]

Supplementary materials

Comparative mitochondrial genome and transcriptome analyses reveal strain-specific features of RNA editing in *Trypanosoma brucei*

Xiaojing Zhao[1†], Yixin He[1†], Fan Zhang[1†#], Inna Aphasizheva[2], Ruslan Aphasizhev[2,*] and Liye Zhang[1,3,*]

[1] School of Life Science and Technology, ShanghaiTech University, Shanghai, 201210, China

[2] Department of Molecular and Cell Biology, Boston University Medical Campus, Boston, MA 02118, USA

[3] Shanghai Clinical Research and Trial Center, Shanghai, 201210, China

* To whom correspondence should be addressed:

Liye Zhang (zhangly@shanghaitech.edu.cn; Tel:+86-021-20685435)

or

Ruslan Aphasizhev (ruslana@bu.edu; Tel: +1 857 337 5076)

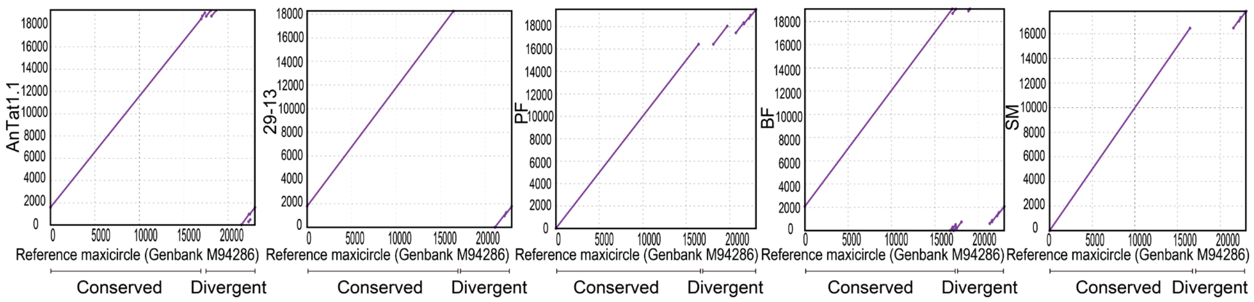

Figure S1. Pairwise alignment between the assembled maxicircle from five cell lines in this study and the reference maxicircle (GenBank: M94286.1; TRBKPGEN locus).

12S rRNA

```
29-13_12S          1 ATTTTACCAATTAAGAAGAATATTATAATAATGGGTGTCTTATATTTTAAATAAATATTTAAATTCCGTGTAGTAAATTTATTATTTGTATTATTTATAT 100
AnTat1.1_12S       1 ATTTTACCAATTAAGAAGAATATTATAATAATGGGTGTCTTATATTTTAAATAAATATTTAAATTCCGTGTAGTAAATTTATTATTTGTATTATTTATAT 100
BF_12S             1 ATTTTACCAATTAAGAAGAATATTATAATAATGGGTGTCTTATATTTTAAATAAATATTTAAATTCCGTGTAGTAAATTTATTATTTGTATTATTTATAT 100
SM_12S             1 ATTTTACCAATTAAGAAGAATATTATAATAATGGGTGTCTTATATTTTAAATAAATATTTAAATTCCGTGTAGTAAATTTATTATTTGTATTATTTATAT 100
PF_12S             1 ATTTTACCAATTAAGAAGAATATTATAATAATGGGTGTCTTATATTTTAAATAAATATTTAAATTCCGTGTAGTAAATTTATTATTTGTATTATTTATAT 100
ref_12S            1 ATTTTACCAATTAAGAAGAATATTATAATAATGGGTGTCTTATATTTTAAATAAATATTTAAATTCCGTGTAGTAAATTTATTATTTGTATTATTTATAT 100

                                                                       *
29-13_12S        101 AATAGGTGTATTATATTTAAATTTTAAATTTGTTGTTTTATATTTAGATACATATTTATAGATTAATATATTTAAATAATATTTTAAAATTTATTGAACT 200
AnTat1.1_12S     101 AATAGGTGTATTATATTTAAATTTTAAATTTGTTGTTTTATATTTAGATATATATTTATAGATTAATATATTTAAATAATATTTTAAAATTTATTGAACT 200
BF_12S           101 AATAGGTGTATTATATTTAAATTTTAAATTTGTTGTTTTATATTTAGATACATATTTATAGATTAATATATTTAAATAATATTTTAAAATTTATTGAACT 200
SM_12S           101 AATAGGTGTATTATATTTAAATTTTAAATTTGTTGTTTTATATTTAGATACATATTTATAGATTAATATATTTAAATAATATTTTAAAATTTATTGAACT 200
PF_12S           101 AATAGGTGTATTATATTTAAATTTTAAATTTGTTGTTTTATATTTAGATACATATTTATAGATTAATATATTTAAATAATATTTTAAAATTTATTGAACT 200
ref_12S          101 AATAGGTGTATTATATTTAAATTTTAAATTTGTTGTTTTATATTTAGATACATATTTATAGATTAATATATTTAAATAATATTTTAAAATTTATTGAACT 200

29-13_12S        201 GTAATTATTAGTTTAATATTTTTAGTTTGATGTTGAAATATTTAATTAAAGATGTTACAGTTGTTCTATATGTACCAAATAAATATAGTAAGATTATTTT 300
AnTat1.1_12S     201 GTAATTATTAGTTTAATATTTTTAGTTTGATGTTGAAATATTTAATTAAAGATGTTACAGTTGTTCTATATGTACCAAATAAATATAGTAAGATTATTTT 300
BF_12S           201 GTAATTATTAGTTTAATATTTTTAGTTTGATGTTGAAATATTTAATTAAAGATGTTACAGTTGTTCTATATGTACCAAATAAATATAGTAAGATTATTTT 300
SM_12S           201 GTAATTATTAGTTTAATATTTTTAGTTTGATGTTGAAATATTTAATTAAAGATGTTACAGTTGTTCTATATGTACCAAATAAATATAGTAAGATTATTTT 300
PF_12S           201 GTAATTATTAGTTTAATATTTTTAGTTTGATGTTGAAATATTTAATTAAAGATGTTACAGTTGTTCTATATGTACCAAATAAATATAGTAAGATTATTTT 300
ref_12S          201 GTAATTATTAGTTTAATATTTTTAGTTTGATGTTGAAATATTTAATTAAAGATGTTACAGTTGTTCTATATGTACCAAATAAATATAGTAAGATTATTTT 300

29-13_12S        301 AGTTGAATTAATAAATAAATATTTATTTTTCTTTGTAAATATTATGAACAATTTAAAAATTAATCTGTTTAACTAAAATGTTATATATAATAATCTAAGT 400
AnTat1.1_12S     301 AGTTGAATTAATAAATAAATATTTATTTTTCTTTGTAAATATTATGAACAATTTAAAAATTAATCTGTTTAACTAAAATGTTATATATAATAATCTAAGT 400
BF_12S           301 AGTTGAATTAATAAATAAATATTTATTTTTCTTTGTAAATATTATGAACAATTTAAAAATTAATCTGTTTAACTAAAATGTTATATATAATAATCTAAGT 400
SM_12S           301 AGTTGAATTAATAAATAAATATTTATTTTTCTTTGTAAATATTATGAACAATTTAAAAATTAATCTGTTTAACTAAAATGTTATATATAATAATCTAAGT 400
PF_12S           301 AGTTGAATTAATAAATAAATATTTATTTTTCTTTGTAAATATTATGAACAATTTAAAAATTAATCTGTTTAACTAAAATGTTATATATAATAATCTAAGT 400
ref_12S          301 AGTTGAATTAATAAATAAATATTTATTTTTCTTTGTAAATATTATGAACAATTTAAAAATTAATCTGTTTAACTAAAATGTTATATATAATAATCTAAGT 400

                                                                                                              *
29-13_12S        401 TAATTTGAATATTAAAAGTACAAGTATAATTTGTAATTCTAAAGTATTTTAATGGTATATTTTTAGTAGGTAAATGAAAAGTATAAATGGATATAACTTA 500
AnTat1.1_12S     401 TAATTTGAATATTAAAAGTACAAGTATAATTTGTAATTCTAAAGTATTTTAATGGTATATTTTTAGTAGGTAAATGAAAAGTATAAATGAATATAACTTA 500
BF_12S           401 TAATTTGAATATTAAAAGTACAAGTATAATTTGTAATTCTAAAGTATTTTAATGGTATATTTTTAGTAGGTAAATGAAAAGTATAAATGGATATAACTTA 500
SM_12S           401 TAATTTGAATATTAAAAGTACAAGTATAATTTGTAATTCTAAAGTATTTTAATGGTATATTTTTAGTAGGTAAATGAAAAGTATAAATGGATATAACTTA 500
PF_12S           401 TAATTTGAATATTAAAAGTACAAGTATAATTTGTAATTCTAAAGTATTTTAATGGTATATTTTTAGTAGGTAAATGAAAAGTATAAATGGATATAACTTA 500
ref_12S          401 TAATTTGAATATTAAAAGTACAAGTATAATTTGTAATTCTAAAGTATTTTAATGGTATATTTTTAGTAGGTAAATGAAAAGTATAAATGGATATAACTTA 500

29-13_12S        501 ATATTTAATATTTGTTTAATGAAAAGTATTTTATTATTATATTGTATAGTATTATTATAGTGTATAGTTTTTTAAAAATATAAAAATATTGTTAATAAAA 600
AnTat1.1_12S     501 ATATTTAATATTTGTTTAATGAAAAGTATTTTATTATTATATTGTATAGTATTATTATAGTGTATAGTTTTTTAAAAATATAAAAATATTGTTAATAAAA 600
BF_12S           501 ATATTTAATATTTGTTTAATGAAAAGTATTTTATTATTATATTGTATAGTATTATTATAGTGTATAGTTTTTTAAAAATATAAAAATATTGTTAATAAAA 600
SM_12S           501 ATATTTAATATTTGTTTAATGAAAAGTATTTTATTATTATATTGTATAGTATTATTATAGTGTATAGTTTTTTAAAAATATAAAAATATTGTTAATAAAA 600
PF_12S           501 ATATTTAATATTTGTTTAATGAAAAGTATTTTATTATTATATTGTATAGTATTATTATAGTGTATAGTTTTTTAAAAATATAAAAATATTGTTAATAAAA 600
ref_12S          501 ATATTTAATATTTGTTTAATGAAAAGTATTTTATTATTATATTGTATAGTATTATTATAGTGTATAGTTTTTTAAAAATATAAAAATATTGTTAATAAAA 600

29-13_12S        601 TTATCGTATTTTAAGTGCGTTTATTAAATGCGTTTGTCTAAGATAATTATTTAAGATTATTCTTGTAAATATATTTAAATATTAATAATTCTTAAAATAA 700
AnTat1.1_12S     601 TTATCGTATTTTAAGTGCGTTTATTAAATGCGTTTGTCTAAGATAATTATTTAAGATTATTCTTGTAAATATATTTAAATATTAATAATTCTTAAAATAA 700
BF_12S           601 TTATCGTATTTTAAGTGCGTTTATTAAATGCGTTTGTCTAAGATAATTATTTAAGATTATTCTTGTAAATATATTTAAATATTAATAATTCTTAAAATAA 700
SM_12S           601 TTATCGTATTTTAAGTGCGTTTATTAAATGCGTTTGTCTAAGATAATTATTTAAGATTATTCTTGTAAATATATTTAAATATTAATAATTCTTAAAATAA 700
PF_12S           601 TTATCGTATTTTAAGTGCGTTTATTAAATGCGTTTGTCTAAGATAATTATTTAAGATTATTCTTGTAAATATATTTAAATATTAATAATTCTTAAAATAA 700
ref_12S          601 TTATCGTATTTTAAGTGCGTTTATTAAATGCGTTTGTCTAAGATAATTATTTAAGATTATTCTTGTAAATATATTTAAATATTAATAATTCTTAAAATAA 700

29-13_12S        701 AAAAATATCCTCAATTGCAATATTATTGTAGCATAGTAATTTGTTAACTAAATATTAAAGTGTTCCATAGAAAATTTTTAAATTACAACAAATAAAATAA 800
AnTat1.1_12S     701 AAAAATATCCTCAATTGCAATATTATTGTAGCATAGTAATTTGTTAACTAAATATTAAAGTGTTCCATAGAAAATTTTTAAATTACAACAAATAAAATAA 800
BF_12S           701 AAAAATATCCTCAATTGCAATATTATTGTAGCATAGTAATTTGTTAACTAAATATTAAAGTGTTCCATAGAAAATTTTTAAATTACAACAAATAAAATAA 800
SM_12S           701 AAAAATATCCTCAATTGCAATATTATTGTAGCATAGTAATTTGTTAACTAAATATTAAAGTGTTCCATAGAAAATTTTTAAATTACAACAAATAAAATAA 800
PF_12S           701 AAAAATATCCTCAATTGCAATATTATTGTAGCATAGTAATTTGTTAACTAAATATTAAAGTGTTCCATAGAAAATTTTTAAATTACAACAAATAAAATAA 800
ref_12S          701 AAAAATATCCTCAATTGCAATATTATTGTAGCATAGTAATTTGTTAACTAAATATTAAAGTGTTCCATAGAAAATTTTTAAATTACAACAAATAAAATAA 800

29-13_12S        801 AGTATGAATTAATATCAAAATTTTAATAAAAATTAAAAAATTAAAATAGGGCAAGTCCTACTCTCCTTTACAAAGAGAACATTATGATATGTAATTGTAT 900
AnTat1.1_12S     801 AGTATGAATTAATATCAAAATTTTAATAAAAATTAAAAAATTAAAATAGGGCAAGTCCTACTCTCCTTTACAAAGAGAACATTATGATATGTAATTGTAT 900
BF_12S           801 AGTATGAATTAATATCAAAATTTTAATAAAAATTAAAAAATTAAAATAGGGCAAGTCCTACTCTCCTTTACAAAGAGAACATTATGATATGTAATTGTAT 900
SM_12S           801 AGTATGAATTAATATCAAAATTTTAATAAAAATTAAAAAATTAAAATAGGGCAAGTCCTACTCTCCTTTACAAAGAGAACATTATGATATGTAATTGTAT 900
PF_12S           801 AGTATGAATTAATATCAAAATTTTAATAAAAATTAAAAAATTAAAATAGGGCAAGTCCTACTCTCCTTTACAAAGAGAACATTATGATATGTAATTGTAT 900
ref_12S          801 AGTATGAATTAATATCAAAATTTTAATAAAAATTAAAAAATTAAAATAGGGCAAGTCCTACTCTCCTTTACAAAGAGAACATTATGATATGTAATTGTAT 900

29-13_12S        901 GTTTGATTGGGGCAATACTATATTTATTTATATAGCATAAGAACTATATTCTTTGAAATTATAAAAGGTTCGAGCAGGTTAACAAGCATTAAAAATAAAT 1000
AnTat1.1_12S     901 GTTTGATTGGGGCAATACTATATTTATTTATATAGCATAAGAACTATATTCTTTGAAATTATAAAAGGTTCGAGCAGGTTAACAAGCATTAAAAATAAAT 1000
BF_12S           901 GTTTGATTGGGGCAATACTATATTTATTTATATAGCATAAGAACTATATTCTTTGAAATTATAAAAGGTTCGAGCAGGTTAACAAGCATTAAAAATAAAT 1000
SM_12S           901 GTTTGATTGGGGCAATACTATATTTATTTATATAGCATAAGAACTATATTCTTTGAAATTATAAAAGGTTCGAGCAGGTTAACAAGCATTAAAAATAAAT 1000
PF_12S           901 GTTTGATTGGGGCAATACTATATTTATTTATATAGCATAAGAACTATATTCTTTGAAATTATAAAAGGTTCGAGCAGGTTAACAAGCATTAAAAATAAAT 1000
ref_12S          901 GTTTGATTGGGGCAATACTATATTTATTTATATAGCATAAGAACTATATTCTTTGAAATTATAAAAGGTTCGAGCAGGTTAACAAGCATTAAAAATAAAT 1000

29-13_12S       1001 GTGTTTCATCGTCTACTTATTACCATGATTGATTGTTCATCAAAATAGTAATTCGTTAGTTGGGTTAAAATCGTTGTAAAGCAGATTTGTTTATATATTT 1100
AnTat1.1_12S    1001 GTGTTTCATCGTCTACTTATTACCATGATTGATTGTTCATCAAAATAGTAATTCGTTAGTTGGGTTAAAATCGTTGTAAAGCAGATTTGTTTATATATTT 1100
BF_12S          1001 GTGTTTCATCGTCTACTTATTACCATGATTGATTGTTCATCAAAATAGTAATTCGTTAGTTGGGTTAAAATCGTTGTAAAGCAGATTTGTTTATATATTT 1100
SM_12S          1001 GTGTTTCATCGTCTACTTATTACCATGATTGATTGTTCATCAAAATAGTAATTCGTTAGTTGGGTTAAAATCGTTGTAAAGCAGATTTGTTTATATATTT 1100
PF_12S          1001 GTGTTTCATCGTCTACTTATTACCATGATTGATTGTTCATCAAAATAGTAATTCGTTAGTTGGGTTAAAATCGTTGTAAAGCAGATTTGTTTATATATTT 1100
ref_12S         1001 GTGTTTCATCGTCTACTTATTACCATGATTGATTGTTCATCAAAATAGTAATTCGTTAGTTGGGTTAAAATCGTTGTAAAGCAGATTTGTTTATATATTT 1100

29-13_12S       1101 AATTTTTATAATTAATAATAATTAATATAAGTACGCAAGGATTGATTAT 1149
AnTat1.1_12S    1101 AATTTTTATAATTAATAATAATTAATATAAGTACGCAAGGATTGATTAT 1149
BF_12S          1101 AATTTTTATAATTAATAATAATTAATATAAGTACGCAAGGATTGATTAT 1149
SM_12S          1101 AATTTTTATAATTAATAATAATTAATATAAGTACGCAAGGATTGATTAT 1149
PF_12S          1101 AATTTTTATAATTAATAATAATTAATATAAGTACGCAAGGATTGATTAT 1149
ref_12S         1101 AATTTTTATAATTAATAATAATTAATATAAGTACGCAAGGATTGATTAT 1149
```

9S rRNA

```
                                                                                      *
29-13_9S      1 TAAATTATGGTCAATTGTTAGTATTCATATTAATTTTTTTAAATGTTTTATCATTTTATAAAGGTTTATTTTTGAAAGATTTTTTGTATAAAATTTTAGG 100
AnTat1.1_9S   1 TAAATTATGGTCAATTGTTAGTATTCATATTAATTTTTTTAAATGTTTTATCATTTTATAAAGGTTTATTTTTGAAAGATTTTTTGTATAAAATTTTAGG 100
BF_9S         1 TAAATTATGGTCAATTGTTAGTATTCATATTAATTTTTTTAAATGTTTTATCATTTTATAAAGGTTTATTTTTGAAAGATTTTTTGTATAAAATTTTAGG 100
SM_9S         1 TAAATTATGGTCAATTGTTAGTATTCATATTAATTTTTTTAAATGTTTTATCATTTTATAAAGGTTTATTTTTGAAAGATTTTTTGTATAAAATTTTAGG 100
PF_9S         1 TAAATTATGGTCAATTGTTAGTATTCATATTAATTTTTTTAAATGTTTTATCATTTTATAAAGGTTTATTTTTGAAAGATTTTTTGTATAAAATTTTAGG 100
ref_9S        1 TAAATTATGGTCAATTGTTAGTATTCATATTAATTTTTTTAAATGTTTTATCATTTTATAAAGGTTTATTTTTGAAAGATTTTTTGTATAAAATTTTAGG 100

                                                                                      *
29-13_9S    101 AATAGTTAATAATAATTTATAATTTTGATTAGATTGTTTTGTTAATGCTATTAGATGGGTGTGGAAAAAT-AAAAAAAATAATTAATATATATCAATAAT 199
AnTat1.1_9S 101 AATAGTTAATAATAATTTATAATTTTGATTAGATTGTTTTGTTAATGCTATTAGATGGGTGTGGAAAAATAAAAAAAAATAATTAATATATATCAATAAT 200
BF_9S       101 AATAGTTAATAATAATTTATAATTTTGATTAGATTGTTTTGTTAATGCTATTAGATGGGTGTGGAAAAAT-AAAAAAAATAATTAATATATATCAATAAT 199
SM_9S       101 AATAGTTAATAATAATTTATAATTTTGATTAGATTGTTTTGTTAATGCTATTAGATGGGTGTGGAAAAAT-AAAAAAAATAATTAATATATATCAATAAT 199
PF_9S       101 AATAGTTAATAATAATTTATAATTTTGATTAGATTGTTTTGTTAATGCTATTAGATGGGTGTGGAAAAAT-AAAAAAAATAATTAATATATATCAATAAT 199
ref_9S      101 AATAGTTAATAATAATTTATAATTTTGATTAGATTGTTTTGTTAATGCTATTAGATGGGTGTGGAAAAAT-AAAAAAAATAATTAATATATATCAATAAT 199

29-13_9S    200 AAATTAAATTAATCTATTAGTCAGAAATGGATGCCAGCCGTTGCGGTAATTTCTATGCTTTTAAATATTATACAATTATCATATTAAATTGTTAAGTGCT 299
AnTat1.1_9S 201 AAATTAAATTAATCTATTAGTCAGAAATGGATGCCAGCCGTTGCGGTAATTTCTATGCTTTTAAATATTATACAATTATCATATTAAATTGTTAAGTGCT 300
BF_9S       200 AAATTAAATTAATCTATTAGTCAGAAATGGATGCCAGCCGTTGCGGTAATTTCTATGCTTTTAAATATTATACAATTATCATATTAAATTGTTAAGTGCT 299
SM_9S       200 AAATTAAATTAATCTATTAGTCAGAAATGGATGCCAGCCGTTGCGGTAATTTCTATGCTTTTAAATATTATACAATTATCATATTAAATTGTTAAGTGCT 299
PF_9S       200 AAATTAAATTAATCTATTAGTCAGAAATGGATGCCAGCCGTTGCGGTAATTTCTATGCTTTTAAATATTATACAATTATCATATTAAATTGTTAAGTGCT 299
ref_9S      200 AAATTAAATTAATCTATTAGTCAGAAATGGATGCCAGCCGTTGCGGTAATTTCTATGCTTTTAAATATTATACAATTATCATATTAAATTGTTAAGTGCT 299

29-13_9S    300 GATTTAACCAATAAAAATATAAATAATTTTTATTTGTTTTTAAACACCATTAGGTATATGCAAATATAAAATTATAGTAATTATAAATTATATTATATTA 399
AnTat1.1_9S 301 GATTTAACCAATAAAAATATAAATAATTTTTATTTGTTTTTAAACACCATTAGGTATATGCAAATATAAAATTATAGTAATTATAAATTATATTATATTA 400
BF_9S       300 GATTTAACCAATAAAAATATAAATAATTTTTATTTGTTTTTAAACACCATTAGGTATATGCAAATATAAAATTATAGTAATTATAAATTATATTATATTA 399
SM_9S       300 GATTTAACCAATAAAAATATAAATAATTTTTATTTGTTTTTAAACACCATTAGGTATATGCAAATATAAAATTATAGTAATTATAAATTATATTATATTA 399
PF_9S       300 GATTTAACCAATAAAAATATAAATAATTTTTATTTGTTTTTAAACACCATTAGGTATATGCAAATATAAAATTATAGTAATTATAAATTATATTATATTA 399
ref_9S      300 GATTTAACCAATAAAAATATAAATAATTTTTATTTGTTTTTAAACACCATTAGGTATATGCAAATATAAAATTATAGTAATTATAAATTATATTATATTA 399

29-13_9S    400 TATTTATTCATATAATTAATAGGATAATATTTGTAGTTTTGATACCATGATAAGGATTATAAATTGAAAGTGTTAATATCATAATCAAAATTTATTATT 499
AnTat1.1_9S 401 TATTTATTCATATAATTAATAGGATAATATTTGTAGTTTTGATACCATGATAAGGATTATAAATTGAAAGTGTTAATATCATAATCAAAATTTATTATT 500
BF_9S       400 TATTTATTCATATAATTAATAGGATAATATTTGTAGTTTTGATACCATGATAAGGATTATAAATTGAAAGTGTTAATATCATAATCAAAATTTATTATT 499
SM_9S       400 TATTTATTCATATAATTAATAGGATAATATTTGTAGTTTTGATACCATGATAAGGATTATAAATTGAAAGTGTTAATATCATAATCAAAATTTATTATT 499
PF_9S       400 TATTTATTCATATAATTAATAGGATAATATTTGTAGTTTTGATACCATGATAAGGATTATAAATTGAAAGTGTTAATATCATAATCAAAATTTATTATT 499
ref_9S      400 TATTTATTCATATAATTAATAGGATAATATTTGTAGTTTTGATACCATGATAAGGATTATAAATTGAAAGTGTTAATATCATAATCAAAATTTATTATT 499

29-13_9S    500 TATATTAAATATGTATGTGTAGATAAAATAAGAAATTAAAAAGGTATTGTTGCCCACCAATTTTTATAATAAAAATAACGTGCAGTAATTAATATATTTA 599
AnTat1.1_9S 501 TATATTAAATATGTATGTGTAGATAAAATAAGAAATTAAAAAGGTATTGTTGCCCACCAATTTTTATAATAAAAATAACGTGCAGTAATTAATATATTTA 600
BF_9S       500 TATATTAAATATGTATGTGTAGATAAAATAAGAAATTAAAAAGGTATTGTTGCCCACCAATTTTTATAATAAAAATAACGTGCAGTAATTAATATATTTA 599
SM_9S       500 TATATTAAATATGTATGTGTAGATAAAATAAGAAATTAAAAAGGTATTGTTGCCCACCAATTTTTATAATAAAAATAACGTGCAGTAATTAATATATTTA 599
PF_9S       500 TATATTAAATATGTATGTGTAGATAAAATAAGAAATTAAAAAGGTATTGTTGCCCACCAATTTTTATAATAAAAATAACGTGCAGTAATTAATATATTTA 599
ref_9S      500 TATATTAAATATGTATGTGTAGATAAAATAAGAAATTAAAAAGGTATTGTTGCCCACCAATTTTTATAATAAAAATAACGTGCAGTAATTAATATATTTA 599

29-13_9S    600 TAAAAATATATT 611
AnTat1.1_9S 601 TAAAAATATATT 612
BF_9S       600 TAAAAATATATT 611
SM_9S       600 TAAAAATATATT 611
PF_9S       600 TAAAAATATATT 611
ref_9S      600 TAAAAATATATT 611
```

A6

```
                              **
29-13_A6      1 AAAAATAAGTATTT--TGATATTATTAAAGTAAAAGAGGAATTTTGGGCGGAAGAGAAGGAGACAGGAGAGGAAATGAAGGAGAAAGGTTTTGAGAGGGG 98
AnTat1.1_A6   1 AAAAATAAGTATTTTGTGATATTATTAAAGTAAAAGAGGAATTTTGGGCGGAAGAGAAGGAGACAGGAGAGGAAATGAAGGAGAAAGGTTTTGAGAGGGG 100
BF_A6         1 AAAAATAAGTATTT--TGATATTATTAAAGTAAAAGAGGAATTTTGGGCGGAAGAGAAGGAGACAGGAGAGGAAATGAAGGAGAAAGGTTTTGAGAGGGG 98
SM_A6         1 AAAAATAAGTATTT--TGATATTATTAAAGTAAAAGAGGAATTTTGGGCGGAAGAGAAGGAGACAGGAGAGGAAATGAAGGAGAAAGGTTTTGAGAGGGG 98
PF_A6         1 AAAAATAAGTATTT--TGATATTATTAAAGTAAAAGAGGAATTTTGGGCGGAAGAGAAGGAGACAGGAGAGGAAATGAAGGAGAAAGGTTTTGAGAGGGG 98
ref_A6        1 AAAAATAAGTATTT--TGATATTATTAAAGTAAAAGAGGAATTTTGGGCGGAAGAGAAGGAGACAGGAGAGGAAATGAAGGAGAAAGGTTTTGAGAGGGG 98

                                       *
29-13_A6     99 GGTTTTTTGAGGGGAGGAAAAAGAATTTTGAATTTGAACTATTTGTTTAAGTTATGGGAGAGAAGCAAGGAGGAGAAAAGTAGGGGAATTTTGAGGAGAT 198
AnTat1.1_A6 101 GGTTTTTTGAGGGGAGGAAAAAGGATTTTGAATTTGAACTATTTGTTTAAGTTATGGGAGAGAAGCAAGGAGGAGAAAAGTAGGGGAATTTTGAGGAGAT 200
BF_A6        99 GGTTTTTTGAGGGGAGGAAAAAGAATTTTGAATTTGAACTATTTGTTTAAGTTATGGGAGAGAAGCAAGGAGGAGAAAAGTAGGGGAATTTTGAGGAGAT 198
SM_A6        99 GGTTTTTTGAGGGGAGGAAAAAGAATTTTGAATTTGAACTATTTGTTTAAGTTATGGGAGAGAAGCAAGGAGGAGAAAAGTAGGGGAATTTTGAGGAGAT 198
PF_A6        99 GGTTTTTTGAGGGGAGGAAAAAGAATTTTGAATTTGAACTATTTGTTTAAGTTATGGGAGAGAAGCAAGGAGGAGAAAAGTAGGGGAATTTTGAGGAGAT 198
ref_A6       99 GGTTTTTTGAGGGGAGGAAAAAGAATTTTGAATTTGAACTATTTGTTTAAGTTATGGGAGAGAAGCAAGGAGGAGAAAAGTAGGGGAATTTTGAGGAGAT 198

29-13_A6    199 TCTTGGGGAGAGGCGGGCGGGCGACGGCGGTTTTGAAAACACCCATTTTTAGGAGGATAAGAGGGGAGAAAAGGGGAAATGGAATTGGGAATTGCCTTTG 298
AnTat1.1_A6 201 TCTTGGGGAGAGGCGGGCGGGCGACGGCGGTTTTGAAAACACCCATTTTTAGGAGGATAAGAGGGGAGAAAAGGGGAAATGGAATTGGGAATTGCCTTTG 300
BF_A6       199 TCTTGGGGAGAGGCGGGCGGGCGACGGCGGTTTTGAAAACACCCATTTTTAGGAGGATAAGAGGGGAGAAAAGGGGAAATGGAATTGGGAATTGCCTTTG 298
SM_A6       199 TCTTGGGGAGAGGCGGGCGGGCGACGGCGGTTTTGAAAACACCCATTTTTAGGAGGATAAGAGGGGAGAAAAGGGGAAATGGAATTGGGAATTGCCTTTG 298
PF_A6       199 TCTTGGGGAGAGGCGGGCGGGCGACGGCGGTTTTGAAAACACCCATTTTTAGGAGGATAAGAGGGGAGAAAAGGGGAAATGGAATTGGGAATTGCCTTTG 298
ref_A6      199 TCTTGGGGAGAGGCGGGCGGGCGACGGCGGTTTTGAAAACACCCATTTTTAGGAGGATAAGAGGGGAGAAAAGGGGAAATGGAATTGGGAATTGCCTTTG 298

29-13_A6    299 CCAAACTTTTAGAAGAAAGAGCAGGAAAGGTTAGGGGGAGGAGAGAAGAAAGGGAAAGTTGTGATTTTGGAGTTATAGAATAAGATCAAATAAGTTAATA 398
AnTat1.1_A6 301 CCAAACTTTTAGAAGAAAGAGCAGGAAAGGTTAGGGGGAGGAGAGAAGAAAGGGAAAGTTGTGATTTTGGAGTTATAGAATAAGATCAAATAAGTTAATA 400
BF_A6       299 CCAAACTTTTAGAAGAAAGAGCAGGAAAGGTTAGGGGGAGGAGAGAAGAAAGGGAAAGTTGTGATTTTGGAGTTATAGAATAAGATCAAATAAGTTAATA 398
SM_A6       299 CCAAACTTTTAGAAGAAAGAGCAGGAAAGGTTAGGGGGAGGAGAGAAGAAAGGGAAAGTTGTGATTTTGGAGTTATAGAATAAGATCAAATAAGTTAATA 398
PF_A6       299 CCAAACTTTTAGAAGAAAGAGCAGGAAAGGTTAGGGGGAGGAGAGAAGAAAGGGAAAGTTGTGATTTTGGAGTTATAGAATAAGATCAAATAAGTTAATA 398
ref_A6      299 CCAAACTTTTAGAAGAAAGAGCAGGAAAGGTTAGGGGGAGGAGAGAAGAAAGGGAAAGTTGTGATTTTGGAGTTATAGAATAAGATCAAATAAGTTAATA 398

29-13_A6    399 ATA 401
AnTat1.1_A6 401 ATA 403
BF_A6       399 ATA 401
SM_A6       399 ATA 401
PF_A6       399 ATA 401
ref_A6      399 ATA 401
```

CO1

```
29-13_CO1         1 ATAGTTTTGTTTTTATTTTGGTTTTATAAAATGTTTTTTCTATGTCTTGTGTGCTTAAGTGTTTCTCATAAAATGATTGGAATTTGTTATCTTTTAGTTG 100
AnTat1.1_CO1      1 ATAGTTTTGTTTTTATTTTGGTTTTATAAAATGTTTTTTCTATGTCTTGTGTGCTTAAGTGTTTCTCATAAAATGATTGGAATTTGTTATCTTTTAGTTG 100
BF_CO1            1 ATAGTTTTGTTTTTATTTTGGTTTTATAAAATGTTTTTTCTATGTCTTGTGTGCTTAAGTGTTTCTCATAAAATGATTGGAATTTGTTATCTTTTAGTTG 100
SM_CO1            1 ATAGTTTTGTTTTTATTTTGGTTTTATAAAATGTTTTTTCTATGTCTTGTGTGCTTAAGTGTTTCTCATAAAATGATTGGAATTTGTTATCTTTTAGTTG 100
PF_CO1            1 ATAGTTTTGTTTTTATTTTGGTTTTATAAAATGTTTTTTCTATGTCTTGTGTGCTTAAGTGTTTCTCATAAAATGATTGGAATTTGTTATCTTTTAGTTG 100
ref__CO1          1 ATAGTTTTGTTTTTATTTTGGTTTTATAAAATGTTTTTTCTATGTCTTGTGTGCTTAAGTGTTTCTCATAAAATGATTGGAATTTGTTATCTTTTAGTTG 100

29-13_CO1       101 CTATTTTATGTGGATTTATCGGTTATATTTACTCCTTATTTATTAGATTAGAACTTTCTCTAATTGGTTGTGGAGTTTTATTTGGTGATTATCAGTTTTA 200
AnTat1.1_CO1    101 CTATTTTATGTGGATTTATCGGTTATATTTACTCCTTATTTATTAGATTAGAACTTTCTCTAATTGGTTGTGGAGTTTTATTTGGTGATTATCAGTTTTA 200
BF_CO1          101 CTATTTTATGTGGATTTATCGGTTATATTTACTCCTTATTTATTAGATTAGAACTTTCTCTAATTGGTTGTGGAGTTTTATTTGGTGATTATCAGTTTTA 200
SM_CO1          101 CTATTTTATGTGGATTTATCGGTTATATTTACTCCTTATTTATTAGATTAGAACTTTCTCTAATTGGTTGTGGAGTTTTATTTGGTGATTATCAGTTTTA 200
PF_CO1          101 CTATTTTATGTGGATTTATCGGTTATATTTACTCCTTATTTATTAGATTAGAACTTTCTCTAATTGGTTGTGGAGTTTTATTTGGTGATTATCAGTTTTA 200
ref__CO1        101 CTATTTTATGTGGATTTATCGGTTATATTTACTCCTTATTTATTAGATTAGAACTTTCTCTAATTGGTTGTGGAGTTTTATTTGGTGATTATCAGTTTTA 200

29-13_CO1       201 CAACGTACTCATTACTTCACATGGGTTGATTATGGTTTTTGCTTTTATTATGCCTATAACTATGGGTGGGTTTACAAACTATTTTGCGCCCGTCATGGTT 300
AnTat1.1_CO1    201 CAACGTACTCATTACTTCACATGGGTTGATTATGGTTTTTGCTTTTATTATGCCTATAACTATGGGTGGGTTTACAAACTATTTTGCGCCCGTCATGGTT 300
BF_CO1          201 CAACGTACTCATTACTTCACATGGGTTGATTATGGTTTTTGCTTTTATTATGCCTATAACTATGGGTGGGTTTACAAACTATTTTGCGCCCGTCATGGTT 300
SM_CO1          201 CAACGTACTCATTACTTCACATGGGTTGATTATGGTTTTTGCTTTTATTATGCCTATAACTATGGGTGGGTTTACAAACTATTTTGCGCCCGTCATGGTT 300
PF_CO1          201 CAACGTACTCATTACTTCACATGGGTTGATTATGGTTTTTGCTTTTATTATGCCTATAACTATGGGTGGGTTTACAAACTATTTTGCGCCCGTCATGGTT 300
ref__CO1        201 CAACGTACTCATTACTTCACATGGGTTGATTATGGTTTTTGCTTTTATTATGCCTATAACTATGGGTGGGTTTACAAACTATTTTGCGCCCGTCATGGTT 300

                                                       *
29-13_CO1       301 GGGTTTCCCGATATGGTATTTCCTCGTATAAATAATATGAGCTTTTGAATGTTTATTGGAGGATTTGGTTGCTTAGTAAGTGGGTTTTTGACTGAAGAGG 400
AnTat1.1_CO1    301 GGGTTTCCCGATATGGTATTTCCTCGTATAAATAACATGAGCTTTTGAATGTTTATTGGAGGATTTGGTTGCTTAGTAAGTGGGTTTTTGACTGAAGAGG 400
BF_CO1          301 GGGTTTCCCGATATGGTATTTCCTCGTATAAATAATATGAGCTTTTGAATGTTTATTGGAGGATTTGGTTGCTTAGTAAGTGGGTTTTTGACTGAAGAGG 400
SM_CO1          301 GGGTTTCCCGATATGGTATTTCCTCGTATAAATAATATGAGCTTTTGAATGTTTATTGGAGGATTTGGTTGCTTAGTAAGTGGGTTTTTGACTGAAGAGG 400
PF_CO1          301 GGGTTTCCCGATATGGTATTTCCTCGTATAAATAATATGAGCTTTTGAATGTTTATTGGAGGATTTGGTTGCTTAGTAAGTGGGTTTTTGACTGAAGAGG 400
ref__CO1        301 GGGTTTCCCGATATGGTATTTCCTCGTATAAATAATATGAGCTTTTGAATGTTTATTGGAGGATTTGGTTGCTTAGTAAGTGGGTTTTTGACTGAAGAGG 400

                           *
29-13_CO1       401 GTATGGGGGTTGGTTGGACCTTGTATCCTACGTTGATTTGTATTGATTTTCATTCTAGCTTAGCATGTGACTTTATCATTTTCTCTGTGCATTTTCTTGG 500
AnTat1.1_CO1    401 GTATGGGAGTTGGTTGGACCTTGTATCCTACGTTGATTTGTATTGATTTTCATTCTAGCTTAGCATGTGACTTTATCATTTTCTCTGTGCATTTTCTTGG 500
BF_CO1          401 GTATGGGGGTTGGTTGGACCTTGTATCCTACGTTGATTTGTATTGATTTTCATTCTAGCTTAGCATGTGACTTTATCATTTTCTCTGTGCATTTTCTTGG 500
SM_CO1          401 GTATGGGGGTTGGTTGGACCTTGTATCCTACGTTGATTTGTATTGATTTTCATTCTAGCTTAGCATGTGACTTTATCATTTTCTCTGTGCATTTTCTTGG 500
PF_CO1          401 GTATGGGGGTTGGTTGGACCTTGTATCCTACGTTGATTTGTATTGATTTTCATTCTAGCTTAGCATGTGACTTTATCATTTTCTCTGTGCATTTTCTTGG 500
ref__CO1        401 GTATGGGGGTTGGTTGGACCTTGTATCCTACGTTGATTTGTATTGATTTTCATTCTAGCTTAGCATGTGACTTTATCATTTTCTCTGTGCATTTTCTTGG 500

                                                                    *
29-13_CO1       501 TATATCTAGTATATTAAATTCCATTAATGTTGTAGGTACAATTTTTTGCTGTAGACGAAAATATTTTAGCTTTCTTATTTGAACACTTTTTATTTGAGGT 600
AnTat1.1_CO1    501 TATATCTAGTATATTAAATTCCATTAATGTTGTAGGTACAATTTTTTGTTGTAGACGAAAATATTTTAGCTTTCTTATTTGAACACTTTTTATTTGAGGT 600
BF_CO1          501 TATATCTAGTATATTAAATTCCATTAATGTTGTAGGTACAATTTTTTGCTGTAGACGAAAATATTTTAGCTTTCTTATTTGAACACTTTTTATTTGAGGT 600
SM_CO1          501 TATATCTAGTATATTAAATTCCATTAATGTTGTAGGTACAATTTTTTGCTGTAGACGAAAATATTTTAGCTTTCTTATTTGAACACTTTTTATTTGAGGT 600
PF_CO1          501 TATATCTAGTATATTAAATTCCATTAATGTTGTAGGTACAATTTTTTGCTGTAGACGAAAATATTTTAGCTTTCTTATTTGAACACTTTTTATTTGAGGT 600
ref__CO1        501 TATATCTAGTATATTAAATTCCATTAATGTTGTAGGTACAATTTTTTGCTGTAGACGAAAATATTTTAGCTTTCTTATTTGAACACTTTTTATTTGAGGT 600

                                                                                     *
29-13_CO1       601 GCCTTGTTGACATCCATACTTTTGATTATTACACTTCCCGTTTTGGCTGGTGGAGTTACATTATTATTATGCGATAGAAATTTTAACACATCATTTTATG 700
AnTat1.1_CO1    601 GCCTTGTTGACATCCATACTTTTGATTATTACACTTCCCGTTTTGGCTGGTGGAGTTACATTATTGTTATGCGATAGAAATTTTAACACATCATTTTATG 700
BF_CO1          601 GCCTTGTTGACATCCATACTTTTGATTATTACACTTCCCGTTTTGGCTGGTGGAGTTACATTATTATTATGCGATAGAAATTTTAACACATCATTTTATG 700
SM_CO1          601 GCCTTGTTGACATCCATACTTTTGATTATTACACTTCCCGTTTTGGCTGGTGGAGTTACATTATTATTATGCGATAGAAATTTTAACACATCATTTTATG 700
PF_CO1          601 GCCTTGTTGACATCCATACTTTTGATTATTACACTTCCCGTTTTGGCTGGTGGAGTTACATTATTATTATGCGATAGAAATTTTAACACATCATTTTATG 700
ref__CO1        601 GCCTTGTTGACATCCATACTTTTGATTATTACACTTCCCGTTTTGGCTGGTGGAGTTACATTATTATTATGCGATAGAAATTTTAACACATCATTTTATG 700

                              *     *
29-13_CO1       701 ATGTTGTTGGAGGTGGAGATCTAGTTTTATTTCAACACTTATTTTGGTTCTTTGGACATCCAGAGGTTTATATCATAATTCTACCTGTATTTGGATTAGT 800
AnTat1.1_CO1    701 ATGTTGTTGGGGGTGGTGATCTAGTTTTATTTCAACACTTATTTTGGTTCTTTGGACATCCAGAGGTTTATATCATAATTCTACCTGTATTTGGATTAGT 800
BF_CO1          701 ATGTTGTTGGAGGTGGAGATCTAGTTTTATTTCAACACTTATTTTGGTTCTTTGGACATCCAGAGGTTTATATCATAATTCTACCTGTATTTGGATTAGT 800
SM_CO1          701 ATGTTGTTGGAGGTGGAGATCTAGTTTTATTTCAACACTTATTTTGGTTCTTTGGACATCCAGAGGTTTATATCATAATTCTACCTGTATTTGGATTAGT 800
PF_CO1          701 ATGTTGTTGGAGGTGGAGATCTAGTTTTATTTCAACACTTATTTTGGTTCTTTGGACATCCAGAGGTTTATATCATAATTCTACCTGTATTTGGATTAGT 800
ref__CO1        701 ATGTTGTTGGAGGTGGAGATCTAGTTTTATTTCAACACTTATTTTGGTTCTTTGGACATCCAGAGGTTTATATCATAATTCTACCTGTATTTGGATTAGT 800

                                                                                                                       *
29-13_CO1       801 TTCCACTATTATTGAAGTTACAAGTTTTAGATGCGTTTTTAGTTCCGTCGCTATGATTTATTCTATGCTACTGATATCTGTATTAGGTATGTTTGTTTGA 900
AnTat1.1_CO1    801 TTCCACTATTATTGAAGTTACAAGTTTTAGATGCGTTTTTAGTTCCGTCGCTATGATTTATTCTATGCTACTGATATCTGTATTAGGTATGTTTGTTTGG 900
BF_CO1          801 TTCCACTATTATTGAAGTTACAAGTTTTAGATGCGTTTTTAGTTCCGTCGCTATGATTTATTCTATGCTACTGATATCTGTATTAGGTATGTTTGTTTGA 900
SM_CO1          801 TTCCACTATTATTGAAGTTACAAGTTTTAGATGCGTTTTTAGTTCCGTCGCTATGATTTATTCTATGCTACTGATATCTGTATTAGGTATGTTTGTTTGA 900
PF_CO1          801 TTCCACTATTATTGAAGTTACAAGTTTTAGATGCGTTTTTAGTTCCGTCGCTATGATTTATTCTATGCTACTGATATCTGTATTAGGTATGTTTGTTTGA 900
ref__CO1        801 TTCCACTATTATTGAAGTTACAAGTTTTAGATGCGTTTTTAGTTCCGTCGCTATGATTTATTCTATGCTACTGATATCTGTATTAGGTATGTTTGTTTGA 900

29-13_CO1       901 GCGCATCACATGTTTGTTGTTGGTATGGATGTTGATTCTAGAGCTTATTTTGGAAGTATTACTGTACTAATAGGCTTACCTACATGCATTAAATTATTTA 1000
AnTat1.1_CO1    901 GCGCATCACATGTTTGTTGTTGGTATGGATGTTGATTCTAGAGCTTATTTTGGAAGTATTACTGTACTAATAGGCTTACCTACATGCATTAAATTATTTA 1000
BF_CO1          901 GCGCATCACATGTTTGTTGTTGGTATGGATGTTGATTCTAGAGCTTATTTTGGAAGTATTACTGTACTAATAGGCTTACCTACATGCATTAAATTATTTA 1000
SM_CO1          901 GCGCATCACATGTTTGTTGTTGGTATGGATGTTGATTCTAGAGCTTATTTTGGAAGTATTACTGTACTAATAGGCTTACCTACATGCATTAAATTATTTA 1000
PF_CO1          901 GCGCATCACATGTTTGTTGTTGGTATGGATGTTGATTCTAGAGCTTATTTTGGAAGTATTACTGTACTAATAGGCTTACCTACATGCATTAAATTATTTA 1000
ref__CO1        901 GCGCATCACATGTTTGTTGTTGGTATGGATGTTGATTCTAGAGCTTATTTTGGAAGTATTACTGTACTAATAGGCTTACCTACATGCATTAAATTATTTA 1000

29-13_CO1      1001 ATTGAATCTATAGCTTCTTATTCACAGATATGTGTATTTGTTTTGAAATTTATTTTATATATATGTTTATACTTATGTTTCTTGCTGGTGGATTAACTGG 1100
AnTat1.1_CO1   1001 ATTGAATCTATAGCTTCTTATTCACAGATATGTGTATTTGTTTTGAAATTTATTTTATATATATGTTTATACTTATGTTTCTTGCTGGTGGATTAACTGG 1100
BF_CO1         1001 ATTGAATCTATAGCTTCTTATTCACAGATATGTGTATTTGTTTTGAAATTTATTTTATATATATGTTTATACTTATGTTTCTTGCTGGTGGATTAACTGG 1100
SM_CO1         1001 ATTGAATCTATAGCTTCTTATTCACAGATATGTGTATTTGTTTTGAAATTTATTTTATATATATGTTTATACTTATGTTTCTTGCTGGTGGATTAACTGG 1100
PF_CO1         1001 ATTGAATCTATAGCTTCTTATTCACAGATATGTGTATTTGTTTTGAAATTTATTTTATATATATGTTTATACTTATGTTTCTTGCTGGTGGATTAACTGG 1100
ref__CO1       1001 ATTGAATCTATAGCTTCTTATTCACAGATATGTGTATTTGTTTTGAAATTTATTTTATATATATGTTTATACTTATGTTTCTTGCTGGTGGATTAACTGG 1100

                                                                                    *
29-13_CO1      1101 CTTATTTCTATCAAATGTTGGTATTGATATTTTGATGCATGATACTTATTTTGTTGTTGCACATTTCCACTATGTTTTATCACTTGGTGCTGTTGTAGGG 1200
AnTat1.1_CO1   1101 CTTATTTCTATCAAATGTTGGTATTGATATTTTGATGCATGATACTTATTTTGTTGTTGCGCATTTCCACTATGTTTTATCACTTGGTGCTGTTGTAGGG 1200
BF_CO1         1101 CTTATTTCTATCAAATGTTGGTATTGATATTTTGATGCATGATACTTATTTTGTTGTTGCACATTTCCACTATGTTTTATCACTTGGTGCTGTTGTAGGG 1200
SM_CO1         1101 CTTATTTCTATCAAATGTTGGTATTGATATTTTGATGCATGATACTTATTTTGTTGTTGCACATTTCCACTATGTTTTATCACTTGGTGCTGTTGTAGGG 1200
PF_CO1         1101 CTTATTTCTATCAAATGTTGGTATTGATATTTTGATGCATGATACTTATTTTGTTGTTGCACATTTCCACTATGTTTTATCACTTGGTGCTGTTGTAGGG 1200
ref__CO1       1101 CTTATTTCTATCAAATGTTGGTATTGATATTTTGATGCATGATACTTATTTTGTTGTTGCACATTTCCACTATGTTTTATCACTTGGTGCTGTTGTAGGG 1200

                                                                                      *
29-13_CO1      1201 GTTTTTGGTGGGTTTTTTCATTTTCTCATGAAATGAATTCCTATTGAACTTCACACATTTTGATTATTTTTCTTCATTTCCACATTATGATTTGGTTCTA 1300
AnTat1.1_CO1   1201 GTTTTTGGTGGGTTTTTTCATTTTCTCATGAAATGAATTCCTATTGAACTTCACACATTTTGATTATTTTTCTTCATTTCCACATTATGATTTGGTTCTA 1300
BF_CO1         1201 GTTTTTGGTGGGTTTTTTCATTTTCTCATGAAATGAATTCCTATTGAACTTCACACATTTTGATTATTTTTCTTCATTTCCACATTATGATTTGGTTCTA 1300
SM_CO1         1201 GTTTTTGGTGGGTTTTTTCATTTTCTCATGAAATGAATTCCTATTGAACTTCACACATTTTGATTATTTTTCTTCATTTCCACATTATGATTTGGTTCTA 1300
PF_CO1         1201 GTTTTTGGTGGGTTTTTTCATTTTCTCATGAAATGAATTCCTATTGAACTTCACACATTTTGTTTATTTTTCTTCATTTCCACATTATGATTTGGTTCTA 1300
ref__CO1       1201 GTTTTTGGTGGGTTTTTTCATTTTCTCATGAAATGAATTCCTATTGAACTTCACACATTTTGATTATTTTTCTTCATTTCCACATTATGATTTGGTTCTA 1300

                                                                  *
29-13_CO1      1301 ATATGGTTTTTTTTCCTTTACATAGCTTAGGAATGTTTGCTTTTCCAAGAAGAATTTCTGATTATCCTATCAGCTTTTTATTTTGAAGTGCGTTTACATT 1400
AnTat1.1_CO1   1301 ATATGGTTTTTTTTCCTTTACATAGCTTAGGAATGTTTGCTTTTCCGAGAAGAATTTCTGATTATCCTATCAGCTTTTTATTTTGAAGTGCGTTTACATT 1400
BF_CO1         1301 ATATGGTTTTTTTTCCTTTACATAGCTTAGGAATGTTTGCTTTTCCAAGAAGAATTTCTGATTATCCTATCAGCTTTTTATTTTGAAGTGCGTTTACATT 1400
SM_CO1         1301 ATATGGTTTTTTTTCCTTTACATAGCTTAGGAATGTTTGCTTTTCCAAGAAGAATTTCTGATTATCCTATCAGCTTTTTATTTTGAAGTGCGTTTACATT 1400
PF_CO1         1301 ATATGGTTTTTTTTCCTTTACATAGCTTAGGAATGTTTGCTTTTCCAAGAAGAATTTCTGATTATCCTATCAGCTTTTTATTTTGAAGTGCGTTTACATT 1400
ref__CO1       1301 ATATGGTTTTTTTTCCTTTACATAGCTTAGGAATGTTTGCTTTTCCAAGAAGAATTTCTGATTATCCTATCAGCTTTTTATTTTGAAGTGCGTTTACATT 1400

29-13_CO1      1401 GTATGGTATGCTTTTGTTGACTTTTTTAGTTATATTTTGCTGTTGTTTGTTTAATGTTATATTATTCTGAGATTACTGTTTATTTTTTTATTAATTTGTTT 1500
AnTat1.1_CO1   1401 GTATGGTATGCTTTTGTTGACTTTTTTAGTTATATTTTGCTGTTGTTTGTTTAATGTTATATTATTCTGAGATTACTGTTTATTTTTTTATTAATTTGTTT 1500
BF_CO1         1401 GTATGGTATGCTTTTGTTGACTTTTTTAGTTATATTTTGCTGTTGTTTGTTTAATGTTATATTATTCTGAGATTACTGTTTATTTTTTTATTAATTTGTTT 1500
SM_CO1         1401 GTATGGTATGCTTTTGTTGACTTTTTTAGTTATATTTTGCTGTTGTTTGTTTAATGTTATATTATTCTGAGATTACTGTTTATTTTTTTATTAATTTGTTT 1500
PF_CO1         1401 GTATGGTATGCTTTTGTTGACTTTTTTAGTTATATTTTGCTGTTGTTTGTTTAATGTTATATTATTCTGAGATTACTGTTTATTTTTTTATTAATTTGTTT 1500
ref__CO1       1401 GTATGGTATGCTTTTGTTGACTTTTTTAGTTATATTTTGCTGTTGTTTGTTTAATGTTATATTATTCTGAGATTACTGTTTATTTTTTTATTAATTTGTTT 1500

                                                                                                       *
29-13_CO1      1501 ACATATAGCTTATCTATATTCTTCTATTTTTATACATGAGTACCAGTTTGTATGGCTATTTATTTACTTGTTATTGATTTTGCGCATATAATACTTGACT 1600
AnTat1.1_CO1   1501 ACATATAGCTTATCTATATTCTTCTATTTTTATACATGAGTACCAGTTTGTATGGCTATTTATTTACTTGTTATTGATTTTGCACATATAATACTTGACT 1600
BF_CO1         1501 ACATATAGCTTATCTATATTCTTCTATTTTTATACATGAGTACCAGTTTGTATGGCTATTTATTTACTTGTTATTGATTTTGCGCATATAATACTTGACT 1600
SM_CO1         1501 ACATATAGCTTATCTATATTCTTCTATTTTTATACATGAGTACCAGTTTGTATGGCTATTTATTTACTTGTTATTGATTTTGCGCATATAATACTTGACT 1600
PF_CO1         1501 ACATATAGCTTATCTATATTCTTCTATTTTTATACATGAGTACCAGTTTGTATGGCTATTTATTTACTTGTTATTGATTTTGCGCATATAATACTTGACT 1600
ref__CO1       1501 ACATATAGCTTATCTATATTCTTCTATTTTTATACATGAGTACCAGTTTGTATGGCTATTTATTTACTTGTTATTGATTTTGCGCATATAATACTTGACT 1600

29-13_CO1      1601 ATTTATTAATAATACTTTGCTTTTGCTTCGTATTTTATATTTTTTTTTGACAAGCCTTCCTATTATTCTTTTATATATAA                     1680
AnTat1.1_CO1   1601 ATTTATTAATAATACTTTGCTTTTGCTTCGTATTTTATATTTTTTTTTGACAAGCCTTCCTATTATTCTTTTATATATAA                     1680
BF_CO1         1601 ATTTATTAATAATACTTTGCTTTTGCTTCGTATTTTATATTTTTTTTTGACAAGCCTTCCTATTATTCTTTTATATATAA                     1680
SM_CO1         1601 ATTTATTAATAATACTTTGCTTTTGCTTCGTATTTTATATTTTTTTTTGACAAGCCTTCCTATTATTCTTTTATATATAA                     1680
PF_CO1         1601 ATTTATTAATAATACTTTGCTTTTGCTTCGTATTTTATATTTTTTTTTGACAAGCCTTCCTATTATTCTTTTATATATAA                     1680
ref__CO1       1601 ATTTATTAATAATACTTTGCTTTTGCTTCGTATTTTATATTTTTTTTTGACAAGCCTTCCTATTATTCTTTTATATATAA                     1680
```

CO2

```
                                                                                                                       *
29-13_CO2        1 TAACAAATGAGTTTTATATTAACTTTTTGAATGATATTTTTAATGGATTCAATAATTGTATTAATATCTTTTTCAATATTTCTATCTGTATGAATATGTG 100
AnTat1.1_CO2     1 TAACAAATGAGTTTTATATTAACTTTTTGAATGATATTTTTAATGGATTCAATAATTGTATTAATATCTTTTTCAATATTTCTATCTGTATGAATATGTA 100
BF_CO2           1 TAACAAATGAGTTTTATATTAACTTTTTGAATGATATTTTTAATGGATTCAATAATTGTATTAATATCTTTTTCAATATTTCTATCTGTATGAATATGTG 100
SM_CO2           1 TAACAAATGAGTTTTATATTAACTTTTTGAATGATATTTTTAATGGATTCAATAATTGTATTAATATCTTTTTCAATATTTCTATCTGTATGAATATGTG 100
PF_CO2           1 TAACAAATGAGTTTTATATTAACTTTTTGAATGATATTTTTAATGGATTCAATAATTGTATTAATATCTTTTTCAATATTTCTATCTGTATGAATATGTG 100
ref_CO2          1 TAACAAATGAGTTTTATATTAACTTTTTGAATGATATTTTTAATGGATTCAATAATTGTATTAATATCTTTTTCAATATTTCTATCTGTATGAATATGTG 100

29-13_CO2      101 CATTGATTATAGCAACAGTATTAACTGTAACAAAAATAAATAATATATATTGTACATGAGATTTTATATCATCAAAATTTATAGATACATATTGGTTTGT 200
AnTat1.1_CO2   101 CATTGATTATAGCAACAGTATTAACTGTAACAAAAATAAATAATATATATTGTACATGAGATTTTATATCATCAAAATTTATAGATACATATTGGTTTGT 200
BF_CO2         101 CATTGATTATAGCAACAGTATTAACTGTAACAAAAATAAATAATATATATTGTACATGAGATTTTATATCATCAAAATTTATAGATACATATTGGTTTGT 200
SM_CO2         101 CATTGATTATAGCAACAGTATTAACTGTAACAAAAATAAATAATATATATTGTACATGAGATTTTATATCATCAAAATTTATAGATACATATTGGTTTGT 200
PF_CO2         101 CATTGATTATAGCAACAGTATTAACTGTAACAAAAATAAATAATATATATTGTACATGAGATTTTATATCATCAAAATTTATAGATACATATTGGTTTGT 200
ref_CO2        101 CATTGATTATAGCAACAGTATTAACTGTAACAAAAATAAATAATATATATTGTACATGAGATTTTATATCATCAAAATTTATAGATACATATTGGTTTGT 200

29-13_CO2      201 ACTTGGAATGATGTTTATATTGTGTTTATTGTTAAGGTTGTGTTTGTTGTTGTATTTTAGTTGTATAAATTTTGTGAGTTTTGATTTGTGTAAAGTAATA 300
AnTat1.1_CO2   201 ACTTGGAATGATGTTTATATTGTGTTTATTGTTAAGGTTGTGTTTGTTGTTGTATTTTAGTTGTATAAATTTTGTGAGTTTTGATTTGTGTAAAGTAATA 300
BF_CO2         201 ACTTGGAATGATGTTTATATTGTGTTTATTGTTAAGGTTGTGTTTGTTGTTGTATTTTAGTTGTATAAATTTTGTGAGTTTTGATTTGTGTAAAGTAATA 300
SM_CO2         201 ACTTGGAATGATGTTTATATTGTGTTTATTGTTAAGGTTGTGTTTGTTGTTGTATTTTAGTTGTATAAATTTTGTGAGTTTTGATTTGTGTAAAGTAATA 300
PF_CO2         201 ACTTGGAATGATGTTTATATTGTGTTTATTGTTAAGGTTGTGTTTGTTGTTGTATTTTAGTTGTATAAATTTTGTGAGTTTTGATTTGTGTAAAGTAATA 300
ref_CO2        201 ACTTGGAATGATGTTTATATTGTGTTTATTGTTAAGGTTGTGTTTGTTGTTGTATTTTAGTTGTATAAATTTTGTGAGTTTTGATTTGTGTAAAGTAATA 300

29-13_CO2      301 GGTTTTCAGTGATATTGGGTATATTTTTTTATTTGGAGAAACCACGATATTTAGTAATTTAATATTAGAAAGTGATTATTTAATAGGAGATTTAAGAATAT 400
AnTat1.1_CO2   301 GGTTTTCAGTGATATTGGGTATATTTTTTTATTTGGAGAAACCACGATATTTAGTAATTTAATATTAGAAAGTGATTATTTAATAGGAGATTTAAGAATAT 400
BF_CO2         301 GGTTTTCAGTGATATTGGGTATATTTTTTTATTTGGAGAAACCACGATATTTAGTAATTTAATATTAGAAAGTGATTATTTAATAGGAGATTTAAGAATAT 400
SM_CO2         301 GGTTTTCAGTGATATTGGGTATATTTTTTTATTTGGAGAAACCACGATATTTAGTAATTTAATATTAGAAAGTGATTATTTAATAGGAGATTTAAGAATAT 400
PF_CO2         301 GGTTTTCAGTGATATTGGGTATATTTTTTTATTTGGAGAAACCACGATATTTAGTAATTTAATATTAGAAAGTGATTATTTAATAGGAGATTTAAGAATAT 400
ref_CO2        301 GGTTTTCAGTGATATTGGGTATATTTTTTTATTTGGAGAAACCACGATATTTAGTAATTTAATATTAGAAAGTGATTATTTAATAGGAGATTTAAGAATAT 400

29-13_CO2      401 TACAGTGTAACCATGTATTGACATTGTTAAGTTTGGTTATTTATAAATTATGAGTATCTGCAGTAGATGTAATACACTCATTTACAATATCAAGTTTAGG 500
AnTat1.1_CO2   401 TACAGTGTAACCATGTATTGACATTGTTAAGTTTGGTTATTTATAAATTATGAGTATCTGCAGTAGATGTAATACACTCATTTACAATATCAAGTTTAGG 500
BF_CO2         401 TACAGTGTAACCATGTATTGACATTGTTAAGTTTGGTTATTTATAAATTATGAGTATCTGCAGTAGATGTAATACACTCATTTACAATATCAAGTTTAGG 500
SM_CO2         401 TACAGTGTAACCATGTATTGACATTGTTAAGTTTGGTTATTTATAAATTATGAGTATCTGCAGTAGATGTAATACACTCATTTACAATATCAAGTTTAGG 500
PF_CO2         401 TACAGTGTAACCATGTATTGACATTGTTAAGTTTGGTTATTTATAAATTATGAGTATCTGCAGTAGATGTAATACACTCATTTACAATATCAAGTTTAGG 500
ref_CO2        401 TACAGTGTAACCATGTATTGACATTGTTAAGTTTGGTTATTTATAAATTATGAGTATCTGCAGTAGATGTAATACACTCATTTACAATATCAAGTTTAGG 500

29-13_CO2      501 TATAAAAGTAGAGAACCTGGTAGGTGTAATGAAATAATTTTGTTTGCTACAAATAACGCAACTCTTTACGGACAATGTAGTGAATTGTGTGGTGTATTAC 600
AnTat1.1_CO2   501 TATAAAAGTAGAGAACCTGGTAGGTGTAATGAAATAATTTTGTTTGCTACAAATAACGCAACTCTTTACGGACAATGTAGTGAATTGTGTGGTGTATTAC 600
BF_CO2         501 TATAAAAGTAGAGAACCTGGTAGGTGTAATGAAATAATTTTGTTTGCTACAAATAACGCAACTCTTTACGGACAATGTAGTGAATTGTGTGGTGTATTAC 600
SM_CO2         501 TATAAAAGTAGAGAACCTGGTAGGTGTAATGAAATAATTTTGTTTGCTACAAATAACGCAACTCTTTACGGACAATGTAGTGAATTGTGTGGTGTATTAC 600
PF_CO2         501 TATAAAAGTAGAGAACCTGGTAGGTGTAATGAAATAATTTTGTTTGCTACAAATAACGCAACTCTTTACGGACAATGTAGTGAATTGTGTGGTGTATTAC 600
ref_CO2        501 TATAAAAGTAGAGAACCTGGTAGGTGTAATGAAATAATTTTGTTTGCTACAAATAACGCAACTCTTTACGGACAATGTAGTGAATTGTGTGGTGTATTAC 600

29-13_CO2      601 ACGGTTTTATGCCTATTGTAATAAATTTTATA 632
AnTat1.1_CO2   601 ACGGTTTTATGCCTATTGTAATAAATTTTATA 632
BF_CO2         601 ACGGTTTTATGCCTATTGTAATAAATTTTATA 632
SM_CO2         601 ACGGTTTTATGCCTATTGTAATAAATTTTATA 632
PF_CO2         601 ACGGTTTTATGCCTATTGTAATAAATTTTATA 632
ref_CO2        601 ACGGTTTTATGCCTATTGTAATAAATTTTATA 632
```

CO3

```
29-13_CO3        1 TTATTGAGGATTGTTTAAAATTGAATAAAAAGGCTTTTTGGAAGGGGATTTTTGGGGGACACCGCCAGAGGAGGAGGGTTTTGGAAGAGTTTGTTTTGAG 100
AnTat1.1_CO3     1 TTATTGAGGATTGTTTAAAATTGAATAAAAAGGCTTTTTGGAAGGGGATTTTTGGGGGACACCGCCAGAGGAGGAGGGTTTTGGAAGAGTTTGTTTTGAG 100
BF_CO3           1 TTATTGAGGATTGTTTAAAATTGAATAAAAAGGCTTTTTGGAAGGGGATTTTTGGGGGACACCGCCAGAGGAGGAGGGTTTTGGAAGAGTTTGTTTTGAG 100
SM_CO3           1 TTATTGAGGATTGTTTAAAATTGAATAAAAAGGCTTTTTGGAAGGGGATTTTTGGGGGACACCGCCAGAGGAGGAGGGTTTTGGAAGAGTTTGTTTTGAG 100
PF_CO3           1 TTATTGAGGATTGTTTAAAATTGAATAAAAAGGCTTTTTGGAAGGGGATTTTTGGGGGACACCGCCAGAGGAGGAGGGTTTTGGAAGAGTTTGTTTTGAG 100
ref_CO3          1 TTATTGAGGATTGTTTAAAATTGAATAAAAAGGCTTTTTGGAAGGGGATTTTTGGGGGACACCGCCAGAGGAGGAGGGTTTTGGAAGAGTTTGTTTTGAG 100

29-13_CO3      101 AGGAGGTTTTGAGGGGAGGGGAGAGAGGGAACGGGAGAGGAACGGACCAGAGAGGAGAGTTGAGGAAGGCGGTTTTGAAGGAGAGGGGAGGCTTTCGGAC 200
AnTat1.1_CO3   101 AGGAGGTTTTGAGGGGAGGGGAGAGAGGGAACGGGAGAGGAACGGACCAGAGAGGAGAGTTGAGGAAGGCGGTTTTGAAGGAGAGGGGAGGCTTTCGGAC 200
BF_CO3         101 AGGAGGTTTTGAGGGGAGGGGAGAGAGGGAACGGGAGAGGAACGGACCAGAGAGGAGAGTTGAGGAAGGCGGTTTTGAAGGAGAGGGGAGGCTTTCGGAC 200
SM_CO3         101 AGGAGGTTTTGAGGGGAGGGGAGAGAGGGAACGGGAGAGGAACGGACCAGAGAGGAGAGTTGAGGAAGGCGGTTTTGAAGGAGAGGGGAGGCTTTCGGAC 200
PF_CO3         101 AGGAGGTTTTGAGGGGAGGGGAGAGAGGGAACGGGAGAGGAACGGACCAGAGAGGAGAGTTGAGGAAGGCGGTTTTGAAGGAGAGGGGAGGCTTTCGGAC 200
ref_CO3        101 AGGAGGTTTTGAGGGGAGGGGAGAGAGGGAACGGGAGAGGAACGGACCAGAGAGGAGAGTTGAGGAAGGCGGTTTTGAAGGAGAGGGGAGGCTTTCGGAC 200

29-13_CO3      201 CAAGGGAAGGAAGGGAGGTTAAGAAAAGGAAAAACAATTTGTGAGGGAGAAGGGTTTTTGGAGGGGTTTTGGGAAGAGAGGGGTTTTGGGGAAACCAGAT 300
AnTat1.1_CO3   201 CAAGGGAAGGAAGGGAGGTTAAGAAAAGGAAAAACAATTTGTGAGGGAGAAGGGTTTTTGGAGGGGTTTTGGGAAGAGAGGGGTTTTGGGGAAACCAGAT 300
BF_CO3         201 CAAGGGAAGGAAGGGAGGTTAAGAAAAGGAAAAACAATTTGTGAGGGAGAAGGGTTTTTGGAGGGGTTTTGGGAAGAGAGGGGTTTTGGGGAAACCAGAT 300
SM_CO3         201 CAAGGGAAGGAAGGGAGGTTAAGAAAAGGAAAAACAATTTGTGAGGGAGAAGGGTTTTTGGAGGGGTTTTGGGAAGAGAGGGGTTTTGGGGAAACCAGAT 300
PF_CO3         201 CAAGGGAAGGAAGGGAGGTTAAGAAAAGGAAAAACAATTTGTGAGGGAGAAGGGTTTTTGGAGGGGTTTTGGGAAGAGAGGGGTTTTGGGGAAACCAGAT 300
ref_CO3        201 CAAGGGAAGGAAGGGAGGTTAAGAAAAGGAAAAACAATTTGTGAGGGAGAAGGGTTTTTGGAGGGGTTTTGGGAAGAGAGGGGTTTTGGGGAAACCAGAT 300

29-13_CO3      301 GAGATTGTTTGCAGAAACAAAGGGGTTTTTGGGCAAAGGAATACAATTTGCAGAGGGGGGAGAGCGGAAGGAGGAACACGGGAGGGAAGACAGGATTTAG 400
AnTat1.1_CO3   301 GAGATTGTTTGCAGAAACAAAGGGGTTTTTGGGCAAAGGAATACAATTTGCAGAGGGGGGAGAGCGGAAGGAGGAACACGGGAGGGAAGACAGGATTTAG 400
BF_CO3         301 GAGATTGTTTGCAGAAACAAAGGGGTTTTTGGGCAAAGGAATACAATTTGCAGAGGGGGGAGAGCGGAAGGAGGAACACGGGAGGGAAGACAGGATTTAG 400
SM_CO3         301 GAGATTGTTTGCAGAAACAAAGGGGTTTTTGGGCAAAGGAATACAATTTGCAGAGGGGGGAGAGCGGAAGGAGGAACACGGGAGGGAAGACAGGATTTAG 400
PF_CO3         301 GAGATTGTTTGCAGAAACAAAGGGGTTTTTGGGCAAAGGAATACAATTTGCAGAGGGGGGAGAGCGGAAGGAGGAACACGGGAGGGAAGACAGGATTTAG 400
ref_CO3        301 GAGATTGTTTGCAGAAACAAAGGGGTTTTTGGGCAAAGGAATACAATTTGCAGAGGGGGGAGAGCGGAAGGAGGAACACGGGAGGGAAGACAGGATTTAG 400

29-13_CO3      401 GAAGCGAGAGAGAGGAGAGGGGAAAGGGTTTAGTTGGAATGAAGAGGTAGTTTGTAGGAAGTT 463
AnTat1.1_CO3   401 GAAGCGAGAGAGAGGAGAGGGGAAAGGGTTTAGTTGGAATGAAGAGGTAGTTTGTAGGAAGTT 463
BF_CO3         401 GAAGCGAGAGAGAGGAGAGGGGAAAGGGTTTAGTTGGAATGAAGAGGTAGTTTGTAGGAAGTT 463
SM_CO3         401 GAAGCGAGAGAGAGGAGAGGGGAAAGGGTTTAGTTGGAATGAAGAGGTAGTTTGTAGGAAGTT 463
PF_CO3         401 GAAGCGAGAGAGAGGAGAGGGGAAAGGGTTTAGTTGGAATGAAGAGGTAGTTTGTAGGAAGTT 463
ref_CO3        401 GAAGCGAGAGAGAGGAGAGGGGAAAGGGTTTAGTTGGAATGAAGAGGTAGTTTGTAGGAAGTT 463
```

CR3

```
                         *
29-13_CR3     1 AGAAATATAAATATGTGTATGATATATAAAAACAAGGATTTTTTGGGGGTTTAGGGACAGAGGGTTTATTTTTGAGGATTTTAGGAGGAGAAAAGGGATG 100
AnTat1.1_CR3  1 AGAAATATGAATATGTGTATGATATATAAAAACAAGGATTTTTTGGGGGTTTAGGGACAGAGGGTTTATTTTTGAGGATTTTAGGAGGAGAAAAGGGATG 100
BF_CR3        1 AGAAATATAAATATGTGTATGATATATAAAAACAAGGATTTTTTGGGGGTTTAGGGACAGAGGGTTTATTTTTGAGGATTTTAGGAGGAGAAAAGGGATG 100
SM_CR3        1 AGAAATATAAATATGTGTATGATATATAAAAACAAGGATTTTTTGGGGGTTTAGGGACAGAGGGTTTATTTTTGAGGATTTTAGGAGGAGAAAAGGGATG 100
PF_CR3        1 AGAAATATAAATATGTGTATGATATATAAAAACAAGGATTTTTTGGGGGTTTAGGGACAGAGGGTTTATTTTTGAGGATTTTAGGAGGAGAAAAGGGATG 100
ref_CR3       1 AGAAATATAAATATGTGTATGATATATAAAAACAAGGATTTTTTGGGGGTTTAGGGACAGAGGGTTTATTTTTGAGGATTTTAGGAGGAGAAAAGGGATG 100

29-13_CR3   101 GGAAACAGAAGGACATAAGAAAAGTTTCGTTATTAGATTAAAAAAGTATGCAAATAATTTTTGT                                     164
AnTat1.1_CR3 101 GGAAACAGAAGGACATAAGAAAAGTTTCGTTATTAGATTAAAAAAGTATGCAAATAATTTTTGT                                    164
BF_CR3      101 GGAAACAGAAGGACATAAGAAAAGTTTCGTTATTAGATTAAAAAAGTATGCAAATAATTTTTGT                                     164
SM_CR3      101 GGAAACAGAAGGACATAAGAAAAGTTTCGTTATTAGATTAAAAAAGTATGCAAATAATTTTTGT                                     164
PF_CR3      101 GGAAACAGAAGGACATAAGAAAAGTTTCGTTATTAGATTAAAAAAGTATGCAAATAATTTTTGT                                     164
ref_CR3     101 GGAAACAGAAGGACATAAGAAAAGTTTCGTTATTAGATTAAAAAAGTATGCAAATAATTTTTGT                                     164
```

CR4

```
                                                          *
29-13_CR4     1 TAATTTATTGTTATCTTTGTGTATTTATTAAAAAGGGGC-TTTAAGTTTGGTTGGGATTGATCTATGAGGAAAGGGGGGTTTGTGGGGGAGAGGAAGGGG  99
AnTat1.1_CR4  1 TAATTTATTGTTATCTTTGTGTATTTATTAAAAAGGGGCTTTTAAGTTTGGTTGGGATTGATCTATGAGGAAAGGGGGGTTTGTGGGGGAGAGGAAGGGG 100
BF_CR4        1 TAATTTATTGTTATCTTTGTGTATTTATTAAAAAGGGGC-TTTAAGTTTGGTTGGGATTGATCTATGAGGAAAGGGGGGTTTGTGGGGGAGAGGAAGGGG  99
SM_CR4        1 TAATTTATTGTTATCTTTGTGTATTTATTAAAAAGGGGC-TTTAAGTTTGGTTGGGATTGATCTATGAGGAAAGGGGGGTTTGTGGGGGAGAGGAAGGGG  99
PF_CR4        1 TAATTTATTGTTATCTTTGTGTATTTATTAAAAAGGGGC-TTTAAGTTTGGTTGGGATTGATCTATGAGGAAAGGGGGGTTTGTGGGGGAGAGGAAGGGG  99
ref_CR4       1 TAATTTATTGTTATCTTTGTGTATTTATTAAAAAGGGGC-TTTAAGTTTGGTTGGGATTGATCTATGAGGAAAGGGGGGTTTGTGGGGGAGAGGAAGGGG  99

                 *                                      *
29-13_CR4   100 GATTTGGGGAAGTTGGGTTTAGAAGAGGACGAAATTGAAGGGGAGTTTGATTGGGGGGTTTTAGTTGGGGAGAAAGTGTGGGGTTTTGTGGGGAGGAGGG 199
AnTat1.1_CR4 101 GGTTTGGGGAAGTTGGGTTTAGAAGAGGACGAAATTGAAGGGGGGTTTGATTGGGGGGTTTTAGTTGGGGAGAAAGTGTGGGGTTTTGTGGGGAGGAGGG 200
BF_CR4      100 GATTTGGGGAAGTTGGGTTTAGAAGAGGACGAAATTGAAGGGGAGTTTGATTGGGGGGTTTTAGTTGGGGAGAAAGTGTGGGGTTTTGTGGGGAGGAGGG 199
SM_CR4      100 GATTTGGGGAAGTTGGGTTTAGAAGAGGACGAAATTGAAGGGGAGTTTGATTGGGGGGTTTTAGTTGGGGAGAAAGTGTGGGGTTTTGTGGGGAGGAGGG 199
PF_CR4      100 GATTTGGGGAAGTTGGGTTTAGAAGAGGACGAAATTGAAGGGGAGTTTGATTGGGGGGTTTTAGTTGGGGAGAAAGTGTGGGGTTTTGTGGGGAGGAGGG 199
ref_CR4     100 GATTTGGGGAAGTTGGGTTTAGAAGAGGACGAAATTGAAGGGGAGTTTGATTGGGGGGTTTTAGTTGGGGAGAAAGTGTGGGGTTTTGTGGGGAGGAGGG 199

                                   *
29-13_CR4   200 GGAGAGGGGGTTGATGGAA-TTTTGGTTTATAATTGCGAAGAAATAGTTTTGTTTTTATTTTGGTTTTATAAAATGTTTTTTCT                 282
AnTat1.1_CR4 201 GGAGAGGGGGTTGATGGAATTTTTGGTTTATAATTGCGAAGAAATAGTTTTGTTTTTATTTTGGTTTTATAAAATGTTTTTTCT                284
BF_CR4      200 GGAGAGGGGGTTGATGGAA-TTTTGGTTTATAATTGCGAAGAAATAGTTTTGTTTTTATTTTGGTTTTATAAAATGTTTTTTCT                 282
SM_CR4      200 GGAGAGGGGGTTGATGGAA-TTTTGGTTTATAATTGCGAAGAAATAGTTTTGTTTTTATTTTGGTTTTATAAAATGTTTTTTCT                 282
PF_CR4      200 GGAGAGGGGGTTGATGGAA-TTTTGGTTTATAATTGCGAAGAAATAGTTTTGTTTTTATTTTGGTTTTATAAAATGTTTTTTCT                 282
ref_CR4     200 GGAGAGGGGGTTGATGGAA-TTTTGGTTTATAATTGCGAAGAAATAGTTTTGTTTTTATTTTGGTTTTATAAAATGTTTTTTCT                 282
```

CYB

```
                                                                                                     *
29-13_CYB        1 GTTAAGAATAATGGTTATAAATTTTATATAAAAGCGGAGAAAAAAGAAAGGGTCTTTTAATGTCAGGTTGTTTATATAGAATATATGGGGTAGGTTTTAG  100
AnTat1.1_CYB     1 GTTAAGAATAATGGTTATAAATTTTATATAAAAGCGGAGAAAAAAGAAAGGGTCTTTTAATGTCAGGTTGTTTATACAGAATATATGGGGTAGGTTTTAG  100
BF_CYB           1 GTTAAGAATAATGGTTATAAATTTTATATAAAAGCGGAGAAAAAAGAAAGGGTCTTTTAATGTCAGGTTGTTTATATAGAATATATGGGGTAGGTTTTAG  100
SM_CYB           1 GTTAAGAATAATGGTTATAAATTTTATATAAAAGCGGAGAAAAAAGAAAGGGTCTTTTAATGTCAGGTTGTTTATATAGAATATATGGGGTAGGTTTTAG  100
PF_CYB           1 GTTAAGAATAATGGTTATAAATTTTATATAAAAGCGGAGAAAAAAGAAAGGGTCTTTTAATGTCAGGTTGTTTATATAGAATATATGGGGTAGGTTTTAG  100
ref_CYB          1 GTTAAGAATAATGGTTATAAATTTTATATAAAAGCGGAGAAAAAAGAAAGGGTCTTTTAATGTCAGGTTGTTTATATAGAATATATGGGGTAGGTTTTAG  100

29-13_CYB      101 TTTAGGATTTTTTATAGCATTGCAAATAATTTGTGGAGTGTGTTTAGCTTGATTATTTTTTAGTTGTTTTATTTGTTCAAATTGATATTTTGTATTATTT  200
AnTat1.1_CYB   101 TTTAGGATTTTTTATAGCATTGCAAATAATTTGTGGAGTGTGTTTAGCTTGATTATTTTTTAGTTGTTTTATTTGTTCAAATTGATATTTTGTATTATTT  200
BF_CYB         101 TTTAGGATTTTTTATAGCATTGCAAATAATTTGTGGAGTGTGTTTAGCTTGATTATTTTTTAGTTGTTTTATTTGTTCAAATTGATATTTTGTATTATTT  200
SM_CYB         101 TTTAGGATTTTTTATAGCATTGCAAATAATTTGTGGAGTGTGTTTAGCTTGATTATTTTTTAGTTGTTTTATTTGTTCAAATTGATATTTTGTATTATTT  200
PF_CYB         101 TTTAGGATTTTTTATAGCATTGCAAATAATTTGTGGAGTGTGTTTAGCTTGATTATTTTTTAGTTGTTTTATTTGTTCAAATTGATATTTTGTATTATTT  200
ref_CYB        101 TTTAGGATTTTTTATAGCATTGCAAATAATTTGTGGAGTGTGTTTAGCTTGATTATTTTTTAGTTGTTTTATTTGTTCAAATTGATATTTTGTATTATTT  200

29-13_CYB      201 TTATGAGATTTTGATTTGGGTTTTGTGATAAGAAGTGTACATATATGTTTTACATCTTTATTATATTTACTATTATATATCCATATATTTAAGTCAATAA  300
AnTat1.1_CYB   201 TTATGAGATTTTGATTTGGGTTTTGTGATAAGAAGTGTACATATATGTTTTACATCTTTATTATATTTACTATTATATATCCATATATTTAAGTCAATAA  300
BF_CYB         201 TTATGAGATTTTGATTTGGGTTTTGTGATAAGAAGTGTACATATATGTTTTACATCTTTATTATATTTACTATTATATATCCATATATTTAAGTCAATAA  300
SM_CYB         201 TTATGAGATTTTGATTTGGGTTTTGTGATAAGAAGTGTACATATATGTTTTACATCTTTATTATATTTACTATTATATATCCATATATTTAAGTCAATAA  300
PF_CYB         201 TTATGAGATTTTGATTTGGGTTTTGTGATAAGAAGTGTACATATATGTTTTACATCTTTATTATATTTACTATTATATATCCATATATTTAAGTCAATAA  300
ref_CYB        201 TTATGAGATTTTGATTTGGGTTTTGTGATAAGAAGTGTACATATATGTTTTACATCTTTATTATATTTACTATTATATATCCATATATTTAAGTCAATAA  300

                                                                         *
29-13_CYB      301 CGTTAATAATATTGTTTGACACACATATATTAGTATGATTTATAGGTTTTATATTGTTTGTATTTATAATAATAATAGCTTTTATAGGATATGTACTGCC  400
AnTat1.1_CYB   301 CGTTAATAATATTGTTTGACACACATATATTAGTATGATTTATAGGTTTTATATTGTTTGTATTCATAATAATAATAGCTTTTATAGGATATGTACTGCC  400
BF_CYB         301 CGTTAATAATATTGTTTGACACACATATATTAGTATGATTTATAGGTTTTATATTGTTTGTATTTATAATAATAATAGCTTTTATAGGATATGTACTGCC  400
SM_CYB         301 CGTTAATAATATTGTTTGACACACATATATTAGTATGATTTATAGGTTTTATATTGTTTGTATTTATAATAATAATAGCTTTTATAGGATATGTACTGCC  400
PF_CYB         301 CGTTAATAATATTGTTTGACACACATATATTAGTATGATTTATAGGTTTTATATTGTTTGTATTTATAATAATAATAGCTTTTATAGGATATGTACTGCC  400
ref_CYB        301 CGTTAATAATATTGTTTGACACACATATATTAGTATGATTTATAGGTTTTATATTGTTTGTATTTATAATAATAATAGCTTTTATAGGATATGTACTGCC  400

29-13_CYB      401 TTGTACAATGATGTCATACTGAGGTTTAACGGTGTTTAGTAATATTATAGCAACAGTACCAATTTTAGGTATATGATTATGTTATTGAATTTGGGGAAGT  500
AnTat1.1_CYB   401 TTGTACAATGATGTCATACTGAGGTTTAACGGTGTTTAGTAATATTATAGCAACAGTACCAATTTTAGGTATATGATTATGTTATTGAATTTGGGGAAGT  500
BF_CYB         401 TTGTACAATGATGTCATACTGAGGTTTAACGGTGTTTAGTAATATTATAGCAACAGTACCAATTTTAGGTATATGATTATGTTATTGAATTTGGGGAAGT  500
SM_CYB         401 TTGTACAATGATGTCATACTGAGGTTTAACGGTGTTTAGTAATATTATAGCAACAGTACCAATTTTAGGTATATGATTATGTTATTGAATTTGGGGAAGT  500
PF_CYB         401 TTGTACAATGATGTCATACTGAGGTTTAACGGTGTTTAGTAATATTATAGCAACAGTACCAATTTTAGGTATATGATTATGTTATTGAATTTGGGGAAGT  500
ref_CYB        401 TTGTACAATGATGTCATACTGAGGTTTAACGGTGTTTAGTAATATTATAGCAACAGTACCAATTTTAGGTATATGATTATGTTATTGAATTTGGGGAAGT  500

29-13_CYB      501 GAATTTATAAACGATTTTACATTATTAAAGTTACATGTATTACATGTGTTATTACCATTTATATTACTAATAATATTAATTTTACATTTATTTTGTCTAC  600
AnTat1.1_CYB   501 GAATTTATAAACGATTTTACATTATTAAAGTTACATGTATTACATGTGTTATTACCATTTATATTACTAATAATATTAATTTTACATTTATTTTGTCTAC  600
BF_CYB         501 GAATTTATAAACGATTTTACATTATTAAAGTTACATGTATTACATGTGTTATTACCATTTATATTACTAATAATATTAATTTTACATTTATTTTGTCTAC  600
SM_CYB         501 GAATTTATAAACGATTTTACATTATTAAAGTTACATGTATTACATGTGTTATTACCATTTATATTACTAATAATATTAATTTTACATTTATTTTGTCTAC  600
PF_CYB         501 GAATTTATAAACGATTTTACATTATTAAAGTTACATGTATTACATGTGTTATTACCATTTATATTACTAATAATATTAATTTTACATTTATTTTGTCTAC  600
ref_CYB        501 GAATTTATAAACGATTTTACATTATTAAAGTTACATGTATTACATGTGTTATTACCATTTATATTACTAATAATATTAATTTTACATTTATTTTGTCTAC  600

29-13_CYB      601 ATTATTTTATGAGTTCTGATGCATTTTGTGATAGGTTTGCATTTTATTGTGAAAGATTAAGTTTTTGTATGTGGTTTTATTTGAGAGATATGTTTTTAGC  700
AnTat1.1_CYB   601 ATTATTTTATGAGTTCTGATGCATTTTGTGATAGGTTTGCATTTTATTGTGAAAGATTAAGTTTTTGTATGTGGTTTTATTTGAGAGATATGTTTTTAGC  700
BF_CYB         601 ATTATTTTATGAGTTCTGATGCATTTTGTGATAGGTTTGCATTTTATTGTGAAAGATTAAGTTTTTGTATGTGGTTTTATTTGAGAGATATGTTTTTAGC  700
SM_CYB         601 ATTATTTTATGAGTTCTGATGCATTTTGTGATAGGTTTGCATTTTATTGTGAAAGATTAAGTTTTTGTATGTGGTTTTATTTGAGAGATATGTTTTTAGC  700
PF_CYB         601 ATTATTTTATGAGTTCTGATGCATTTTGTGATAGGTTTGCATTTTATTGTGAAAGATTAAGTTTTTGTATGTGGTTTTATTTGAGAGATATGTTTTTAGC  700
ref_CYB        601 ATTATTTTATGAGTTCTGATGCATTTTGTGATAGGTTTGCATTTTATTGTGAAAGATTAAGTTTTTGTATGTGGTTTTATTTGAGAGATATGTTTTTAGC  700

29-13_CYB      701 ATTTTCAATATTATTATGTATGATGTATGTTATATTTATAAATTGGTATTTTGTATTTCATGAGGAATCTTGAGTTATAGTAGATACACTAAAAACATCA  800
AnTat1.1_CYB   701 ATTTTCAATATTATTATGTATGATGTATGTTATATTTATAAATTGGTATTTTGTATTTCATGAGGAATCTTGAGTTATAGTAGATACACTAAAAACATCA  800
BF_CYB         701 ATTTTCAATATTATTATGTATGATGTATGTTATATTTATAAATTGGTATTTTGTATTTCATGAGGAATCTTGAGTTATAGTAGATACACTAAAAACATCA  800
SM_CYB         701 ATTTTCAATATTATTATGTATGATGTATGTTATATTTATAAATTGGTATTTTGTATTTCATGAGGAATCTTGAGTTATAGTAGATACACTAAAAACATCA  800
PF_CYB         701 ATTTTCAATATTATTATGTATGATGTATGTTATATTTATAAATTGGTATTTTGTATTTCATGAGGAATCTTGAGTTATAGTAGATACACTAAAAACATCA  800
ref_CYB        701 ATTTTCAATATTATTATGTATGATGTATGTTATATTTATAAATTGGTATTTTGTATTTCATGAGGAATCTTGAGTTATAGTAGATACACTAAAAACATCA  800

29-13_CYB      801 GATAAAATATTACCAGAATGATTTTTTTTGTATTTATTCGGTTTTTTAAAGGCAATCCCAGATAAGTTTATGGGTTTGTTTTTAATGGTTATTTTATTAT  900
AnTat1.1_CYB   801 GATAAAATATTACCAGAATGATTTTTTTTGTATTTATTCGGTTTTTTAAAGGCAATCCCAGATAAGTTTATGGGTTTGTTTTTAATGGTTATTTTATTAT  900
BF_CYB         801 GATAAAATATTACCAGAATGATTTTTTTTGTATTTATTCGGTTTTTTAAAGGCAATCCCAGATAAGTTTATGGGTTTGTTTTTAATGGTTATTTTATTAT  900
SM_CYB         801 GATAAAATATTACCAGAATGATTTTTTTTGTATTTATTCGGTTTTTTAAAGGCAATCCCAGATAAGTTTATGGGTTTGTTTTTAATGGTTATTTTATTAT  900
PF_CYB         801 GATAAAATATTACCAGAATGATTTTTTTTGTATTTATTCGGTTTTTTAAAGGCAATCCCAGATAAGTTTATGGGTTTGTTTTTAATGGTTATTTTATTAT  900
ref_CYB        801 GATAAAATATTACCAGAATGATTTTTTTTGTATTTATTCGGTTTTTTAAAGGCAATCCCAGATAAGTTTATGGGTTTGTTTTTAATGGTTATTTTATTAT  900

29-13_CYB      901 TCTCATTATTTTTATTTATATTGAATTGTATATTATGATTTGTGTATTGTAGAAGTTCATTATTATGATTAACATATTCGTTAATATTATTTTATAGTAT 1000
AnTat1.1_CYB   901 TCTCATTATTTTTATTTATATTGAATTGTATATTATGATTTGTGTATTGTAGAAGTTCATTATTATGATTAACATATTCGTTAATATTATTTTATAGTAT 1000
BF_CYB         901 TCTCATTATTTTTATTTATATTGAATTGTATATTATGATTTGTGTATTGTAGAAGTTCATTATTATGATTAACATATTCGTTAATATTATTTTATAGTAT 1000
SM_CYB         901 TCTCATTATTTTTATTTATATTGAATTGTATATTATGATTTGTGTATTGTAGAAGTTCATTATTATGATTAACATATTCGTTAATATTATTTTATAGTAT 1000
PF_CYB         901 TCTCATTATTTTTATTTATATTGAATTGTATATTATGATTTGTGTATTGTAGAAGTTCATTATTATGATTAACATATTCGTTAATATTATTTTATAGTAT 1000
ref_CYB        901 TCTCATTATTTTTATTTATATTGAATTGTATATTATGATTTGTGTATTGTAGAAGTTCATTATTATGATTAACATATTCGTTAATATTATTTTATAGTAT 1000

                                                                                          *
29-13_CYB     1001 ATGAATGAGTGGTTTTTTAGCATTATATGTAGTATTAGCATATCCAATATGAATGGAATTACAATACTGAGTATTATTATTATTTTTGTTGATAGTGTGT 1100
AnTat1.1_CYB  1001 ATGAATGAGTGGTTTTTTAGCATTATATGTAGTATTAGCATATCCAATATGAATGGAATTACAATACTGGGTATTATTATTATTTTTGTTGATAGTGTGT 1100
BF_CYB        1001 ATGAATGAGTGGTTTTTTAGCATTATATGTAGTATTAGCATATCCAATATGAATGGAATTACAATACTGAGTATTATTATTATTTTTGTTGATAGTGTGT 1100
SM_CYB        1001 ATGAATGAGTGGTTTTTTAGCATTATATGTAGTATTAGCATATCCAATATGAATGGAATTACAATACTGAGTATTATTATTATTTTTGTTGATAGTGTGT 1100
PF_CYB        1001 ATGAATGAGTGGTTTTTTAGCATTATATGTAGTATTAGCATATCCAATATGAATGGAATTACAATACTGAGTATTATTATTATTTTTGTTGATAGTGTGT 1100
ref_CYB       1001 ATGAATGAGTGGTTTTTTAGCATTATATGTAGTATTAGCATATCCAATATGAATGGAATTACAATACTGAGTATTATTATTATTTTTGTTGATAGTGTGT 1100

29-13_CYB     1101 AGGTTAGATTAGTTTAGA                                                                                   1118
AnTat1.1_CYB  1101 AGGTTAGATTAGTTTAGA                                                                                   1118
BF_CYB        1101 AGGTTAGATTAGTTTAGA                                                                                   1118
SM_CYB        1101 AGGTTAGATTAGTTTAGA                                                                                   1118
PF_CYB        1101 AGGTTAGATTAGTTTAGA                                                                                   1118
ref_CYB       1101 AGGTTAGATTAGTTTAGA                                                                                   1118
```

MURF1

```
                                                                                *
29-13_MURF1       1 ATAAACCCTCTGTCCCTAAACCCCCAAAAAATCCTTGTTTTTATATATCATACACATATTTATATTTCTTATTATCTACAGCTTTATTATCTTATGTGAT  100
AnTat1.1_MURF1    1 ATAAACCCTCTGTCCCTAAACCCCCAAAAAATCCTTGTTTTTATATATCATACACATATTCATATTTCTTATTATCTACAGCTTTATTATCTTATGTGAT  100
BF_MURF1          1 ATAAACCCTCTGTCCCTAAACCCCCAAAAAATCCTTGTTTTTATATATCATACACATATTTATATTTCTTATTATCTACAGCTTTATTATCTTATGTGAT  100
SM_MURF1          1 ATAAACCCTCTGTCCCTAAACCCCCAAAAAATCCTTGTTTTTATATATCATACACATATTTATATTTCTTATTATCTACAGCTTTATTATCTTATGTGAT  100
PF_MURF1          1 ATAAACCCTCTGTCCCTAAACCCCCAAAAAATCCTTGTTTTTATATATCATACACATATTTATATTTCTTATTATCTACAGCTTTATTATCTTATGTGAT  100
ref_MURF1         1 ATAAACCCTCTGTCCCTAAACCCCCAAAAAATCCTTGTTTTTATATATCATACACATATTTATATTTCTTATTATCTACAGCTTTATTATCTTATGTGAT  100

                                                                                                             *
29-13_MURF1     101 TATACTACACTAACATTACTAAGCTTTGATTTACTCTGATTATTAATTATTAACCTATTTTGAATAACATTATTAGATTCTTATATTTGTTTCATCTTTA  200
AnTat1.1_MURF1  101 TATACTACACTAACATTACTAAGCTTTGATTTACTCTGATTATTAATTATTAACCTATTTTGAATAACATTATTAGATTCTTATATTTGCTTCATCTTTA  200
BF_MURF1        101 TATACTACACTAACATTACTAAGCTTTGATTTACTCTGATTATTAATTATTAACCTATTTTGAATAACATTATTAGATTCTTATATTTGTTTCATCTTTA  200
SM_MURF1        101 TATACTACACTAACATTACTAAGCTTTGATTTACTCTGATTATTAATTATTAACCTATTTTGAATAACATTATTAGATTCTTATATTTGTTTCATCTTTA  200
PF_MURF1        101 TATACTACACTAACATTACTAAGCTTTGATTTACTCTGATTATTAATTATTAACCTATTTTGAATAACATTATTAGATTCTTATATTTGTTTCATCTTTA  200
ref_MURF1       101 TATACTACACTAACATTACTAAGCTTTGATTTACTCTGATTATTAATTATTAACCTATTTTGAATAACATTATTAGATTCTTATATTTGTTTCATCTTTA  200

29-13_MURF1     201 TACTCTTATTCTTATTTTGCTTTACATTATTTTTTTGCTTTCTATCATTCGATACCAGATTTTTATTCATTATTATTATTATTCAATACATTATTATATT  300
AnTat1.1_MURF1  201 TACTCTTATTCTTATTTTGCTTTACATTATTTTTTTGCTTTCTATCATTCGATACCAGATTTTTATTCATTATTATTATTATTCAATACATTATTATATT  300
BF_MURF1        201 TACTCTTATTCTTATTTTGCTTTACATTATTTTTTTGCTTTCTATCATTCGATACCAGATTTTTATTCATTATTATTATTATTCAATACATTATTATATT  300
SM_MURF1        201 TACTCTTATTCTTATTTTGCTTTACATTATTTTTTTGCTTTCTATCATTCGATACCAGATTTTTATTCATTATTATTATTATTCAATACATTATTATATT  300
PF_MURF1        201 TACTCTTATTCTTATTTTGCTTTACATTATTTTTTTGCTTTCTATCATTCGATACCAGATTTTTATTCATTATTATTATTATTCAATACATTATTATATT  300
ref_MURF1       201 TACTCTTATTCTTATTTTGCTTTACATTATTTTTTTGCTTTCTATCATTCGATACCAGATTTTTATTCATTATTATTATTATTCAATACATTATTATATT  300

29-13_MURF1     301 CTTATTTATATTTATTAATCATATTATTATTATATCAATCCTCTTTGAAATATTTAGCTTATTGCTCTTTTTATTATTAATGTCCAGTAGATTTGGATAT  400
AnTat1.1_MURF1  301 CTTATTTATATTTATTAATCATATTATTATTATATCAATCCTCTTTGAAATATTTAGCTTATTGCTCTTTTTATTATTAATGTCCAGTAGATTTGGATAT  400
BF_MURF1        301 CTTATTTATATTTATTAATCATATTATTATTATATCAATCCTCTTTGAAATATTTAGCTTATTGCTCTTTTTATTATTAATGTCCAGTAGATTTGGATAT  400
SM_MURF1        301 CTTATTTATATTTATTAATCATATTATTATTATATCAATCCTCTTTGAAATATTTAGCTTATTGCTCTTTTTATTATTAATGTCCAGTAGATTTGGATAT  400
PF_MURF1        301 CTTATTTATATTTATTAATCATATTATTATTATATCAATCCTCTTTGAAATATTTAGCTTATTGCTCTTTTTATTATTAATGTCCAGTAGATTTGGATAT  400
ref_MURF1       301 CTTATTTATATTTATTAATCATATTATTATTATATCAATCCTCTTTGAAATATTTAGCTTATTGCTCTTTTTATTATTAATGTCCAGTAGATTTGGATAT  400

29-13_MURF1     401 AAAATTTTAGTTCTATGATATTACTACATGTTAAACTTAATAAATTTTATATTATTATTTATATTATTATATTTTATGATATTAAACTACTGTTTTTTCC  500
AnTat1.1_MURF1  401 AAAATTTTAGTTCTATGATATTACTACATGTTAAACTTAATAAATTTTATATTATTATTTATATTATTATATTTTATGATATTAAACTACTGTTTTTTCC  500
BF_MURF1        401 AAAATTTTAGTTCTATGATATTACTACATGTTAAACTTAATAAATTTTATATTATTATTTATATTATTATATTTTATGATATTAAACTACTGTTTTTTCC  500
SM_MURF1        401 AAAATTTTAGTTCTATGATATTACTACATGTTAAACTTAATAAATTTTATATTATTATTTATATTATTATATTTTATGATATTAAACTACTGTTTTTTCC  500
PF_MURF1        401 AAAATTTTAGTTCTATGATATTACTACATGTTAAACTTAATAAATTTTATATTATTATTTATATTATTATATTTTATGATATTAAACTACTGTTTTTTCC  500
ref_MURF1       401 AAAATTTTAGTTCTATGATATTACTACATGTTAAACTTAATAAATTTTATATTATTATTTATATTATTATATTTTATGATATTAAACTACTGTTTTTTCC  500

29-13_MURF1     501 TATGTGATTTTTGTTTTTTAGTTTTTGATGAAGAATGACTTGGAATTTTATGCCTATTTTACACATTATTAATTTTATTCAAATTATACATCGCCTTTTT  600
AnTat1.1_MURF1  501 TATGTGATTTTTGTTTTTTAGTTTTTGATGAAGAATGACTTGGAATTTTATGCCTATTTTACACATTATTAATTTTATTCAAATTATACATCGCCTTTTT  600
BF_MURF1        501 TATGTGATTTTTGTTTTTTAGTTTTTGATGAAGAATGACTTGGAATTTTATGCCTATTTTACACATTATTAATTTTATTCAAATTATACATCGCCTTTTT  600
SM_MURF1        501 TATGTGATTTTTGTTTTTTAGTTTTTGATGAAGAATGACTTGGAATTTTATGCCTATTTTACACATTATTAATTTTATTCAAATTATACATCGCCTTTTT  600
PF_MURF1        501 TATGTGATTTTTGTTTTTTAGTTTTTGATGAAGAATGACTTGGAATTTTATGCCTATTTTACACATTATTAATTTTATTCAAATTATACATCGCCTTTTT  600
ref_MURF1       501 TATGTGATTTTTGTTTTTTAGTTTTTGATGAAGAATGACTTGGAATTTTATGCCTATTTTACACATTATTAATTTTATTCAAATTATACATCGCCTTTTT  600

                                                                                      *
29-13_MURF1     601 AATTTTGTTTATGGAGCAACTTTATATTAGACTTGGTGTCTTTATTTTTATTTATATGTTAACTTTTTATATTTTGTTTTGTTTTATATTAATAATTCTA  700
AnTat1.1_MURF1  601 AATTTTGTTTATGGAGCAACTTTATATTAGACTTGGTGTCTTTATTTTTATTTATATGTTAACTTTCTATATTTTGTTTTGTTTTATATTAATAATTCTA  700
BF_MURF1        601 AATTTTGTTTATGGAGCAACTTTATATTAGACTTGGTGTCTTTATTTTTATTTATATGTTAACTTTTTATATTTTGTTTTGTTTTATATTAATAATTCTA  700
SM_MURF1        601 AATTTTGTTTATGGAGCAACTTTATATTAGACTTGGTGTCTTTATTTTTATTTATATGTTAACTTTTTATATTTTGTTTTGTTTTATATTAATAATTCTA  700
PF_MURF1        601 AATTTTGTTTATGGAGCAACTTTATATTAGACTTGGTGTCTTTATTTTTATTTATATGTTAACTTTTTATATTTTGTTTTGTTTTATATTAATAATTCTA  700
ref_MURF1       601 AATTTTGTTTATGGAGCAACTTTATATTAGACTTGGTGTCTTTATTTTTATTTATATGTTAACTTTTTATATTTTGTTTTGTTTTATATTAATAATTCTA  700

29-13_MURF1     701 CTAATTAGCTTCATTTATTTTTATATTTTATTCATCAAACTTCTATTATTTCAATCTTGTACATGCGTTTTAATTGGATTAAACAGCTTTGCTATCGTTA  800
AnTat1.1_MURF1  701 CTAATTAGCTTCATTTATTTTTATATTTTATTCATCAAACTTCTATTATTTCAATCTTGTACATGCGTTTTAATTGGATTAAACAGCTTTGCTATCGTTA  800
BF_MURF1        701 CTAATTAGCTTCATTTATTTTTATATTTTATTCATCAAACTTCTATTATTTCAATCTTGTACATGCGTTTTAATTGGATTAAACAGCTTTGCTATCGTTA  800
SM_MURF1        701 CTAATTAGCTTCATTTATTTTTATATTTTATTCATCAAACTTCTATTATTTCAATCTTGTACATGCGTTTTAATTGGATTAAACAGCTTTGCTATCGTTA  800
PF_MURF1        701 CTAATTAGCTTCATTTATTTTTATATTTTATTCATCAAACTTCTATTATTTCAATCTTGTACATGCGTTTTAATTGGATTAAACAGCTTTGCTATCGTTA  800
ref_MURF1       701 CTAATTAGCTTCATTTATTTTTATATTTTATTCATCAAACTTCTATTATTTCAATCTTGTACATGCGTTTTAATTGGATTAAACAGCTTTGCTATCGTTA  800

29-13_MURF1     801 GTTTACTATTTGTGTTAAGTGTGAATAATTTTTGCTTTTTATTCCTAATATTTATTTCAACAAAAAATTATATCTTTTATTTATATTTAAATTTTCACTT  900
AnTat1.1_MURF1  801 GTTTACTATTTGTGTTAAGTGTGAATAATTTTTGCTTTTTATTCCTAATATTTATTTCAACAAAAAATTATATCTTTTATTTATATTTAAATTTTCACTT  900
BF_MURF1        801 GTTTACTATTTGTGTTAAGTGTGAATAATTTTTGCTTTTTATTCCTAATATTTATTTCAACAAAAAATTATATCTTTTATTTATATTTAAATTTTCACTT  900
SM_MURF1        801 GTTTACTATTTGTGTTAAGTGTGAATAATTTTTGCTTTTTATTCCTAATATTTATTTCAACAAAAAATTATATCTTTTATTTATATTTAAATTTTCACTT  900
PF_MURF1        801 GTTTACTATTTGTGTTAAGTGTGAATAATTTTTGCTTTTTATTCCTAATATTTATTTCAACAAAAAATTATATCTTTTATTTATATTTAAATTTTCACTT  900
ref_MURF1       801 GTTTACTATTTGTGTTAAGTGTGAATAATTTTTGCTTTTTATTCCTAATATTTATTTCAACAAAAAATTATATCTTTTATTTATATTTAAATTTTCACTT  900

29-13_MURF1     901 AATTTATAGTATAAGTTTAGTATTACTTATTATTATATACTATTTTTTTATTATATATAATATTTTTGACTTTAAATACAATGAAAACTATTTTTTAATC 1000
AnTat1.1_MURF1  901 AATTTATAGTATAAGTTTAGTATTACTTATTATTATATACTATTTTTTTATTATATATAATATTTTTGACTTTAAATACAATGAAAACTATTTTTTAATC 1000
BF_MURF1        901 AATTTATAGTATAAGTTTAGTATTACTTATTATTATATACTATTTTTTTATTATATATAATATTTTTGACTTTAAATACAATGAAAACTATTTTTTAATC 1000
SM_MURF1        901 AATTTATAGTATAAGTTTAGTATTACTTATTATTATATACTATTTTTTTATTATATATAATATTTTTGACTTTAAATACAATGAAAACTATTTTTTAATC 1000
PF_MURF1        901 AATTTATAGTATAAGTTTAGTATTACTTATTATTATATACTATTTTTTTATTATATATAATATTTTTGACTTTAAATACAATGAAAACTATTTTTTAATC 1000
ref_MURF1       901 AATTTATAGTATAAGTTTAGTATTACTTATTATTATATACTATTTTTTTATTATATATAATATTTTTGACTTTAAATACAATGAAAACTATTTTTTAATC 1000

29-13_MURF1    1001 AACTTCATATTTTTTTCTTTTTTTAATAACTTTTTAATAAGTTTACTACTTGCATGTCTCTTTCTTTGCATTGGAGCTATTCCAATTGTATTTGGATTTT 1100
AnTat1.1_MURF1 1001 AACTTCATATTTTTTTCTTTTTTTAATAACTTTTTAATAAGTTTACTACTTGCATGTCTCTTTCTTTGCATTGGAGCTATTCCAATTGTATTTGGATTTT 1100
BF_MURF1       1001 AACTTCATATTTTTTTCTTTTTTTAATAACTTTTTAATAAGTTTACTACTTGCATGTCTCTTTCTTTGCATTGGAGCTATTCCAATTGTATTTGGATTTT 1100
SM_MURF1       1001 AACTTCATATTTTTTTCTTTTTTTAATAACTTTTTAATAAGTTTACTACTTGCATGTCTCTTTCTTTGCATTGGAGCTATTCCAATTGTATTTGGATTTT 1100
PF_MURF1       1001 AACTTCATATTTTTTTCTTTTTTTAATAACTTTTTAATAAGTTTACTACTTGCATGTCTCTTTCTTTGCATTGGAGCTATTCCAATTGTATTTGGATTTT 1100
ref_MURF1      1001 AACTTCATATTTTTTTCTTTTTTTAATAACTTTTTAATAAGTTTACTACTTGCATGTCTCTTTCTTTGCATTGGAGCTATTCCAATTGTATTTGGATTTT 1100

                                                                                                      *
29-13_MURF1    1101 TTATCAAAGTTTTCTGTCTATTATTACAACTAAGTTACCTTTGTATTTGTATTGGCTTTTTCTTTATAATATGACTTATTATCATTTATATTTTTTATTT 1200
AnTat1.1_MURF1 1101 TTATCAAAGTTTTCTGTCTATTATTACAACTAAGTTACCTTTGTATTTGTATTGGCTTTTTCTTTATAATATGACTTATTATTATTTATATTTTTTATTT 1200
BF_MURF1       1101 TTATCAAAGTTTTCTGTCTATTATTACAACTAAGTTACCTTTGTATTTGTATTGGCTTTTTCTTTATAATATGACTTATTATCATTTATATTTTTTATTT 1200
SM_MURF1       1101 TTATCAAAGTTTTCTGTCTATTATTACAACTAAGTTACCTTTGTATTTGTATTGGCTTTTTCTTTATAATATGACTTATTATCATTTATATTTTTTATTT 1200
PF_MURF1       1101 TTATCAAAGTTTTCTGTCTATTATTACAACTAAGTTACCTTTGTATTTGTATTGGCTTTTTCTTTATAATATGACTTATTATCATTTATATTTTTTATTT 1200
ref_MURF1      1101 TTATCAAAGTTTTCTGTCTATTATTACAACTAAGTTACCTTTGTATTTGTATTGGCTTTTTCTTTATAATATGACTTATTATCATTTATATTTTTTATTT 1200

                                                              *
29-13_MURF1    1201 TAGATTAATTGTTAATATATTTATTTTTTCTTATCAATTTCTTGGTTTTTGAGTTGTAAAATTATCTTTTATCAATATTAAAAATTTATTATTCTTCATT 1300
AnTat1.1_MURF1 1201 TAGATTAATTGTTAATATATTTATTTTTTCTTATCAATTTCTCGGTTTTTGAGTTGTAAAATTATCTTTTATCAATATTAAAAATTTATTATTCTTCATT 1300
BF_MURF1       1201 TAGATTAATTGTTAATATATTTATTTTTTCTTATCAATTTCTTGGTTTTTGAGTTGTAAAATTATCTTTTATCAATATTAAAAATTTATTATTCTTCATT 1300
SM_MURF1       1201 TAGATTAATTGTTAATATATTTATTTTTTCTTATCAATTTCTTGGTTTTTGAGTTGTAAAATTATCTTTTATCAATATTAAAAATTTATTATTCTTCATT 1300
PF_MURF1       1201 TAGATTAATTGTTAATATATTTATTTTTTCTTATCAATTTCTTGGTTTTTGAGTTGTAAAATTATCTTTTATCAATATTAAAAATTTATTATTCTTCATT 1300
ref_MURF1      1201 TAGATTAATTGTTAATATATTTATTTTTTCTTATCAATTTCTTGGTTTTTGAGTTGTAAAATTATCTTTTATCAATATTAAAAATTTATTATTCTTCATT 1300

29-13_MURF1    1301 TGTTCTAGCGTATATATACTTTTCTTTGATATTATTAACTTATTTGATCTTATTCTATAACT                                       1362
AnTat1.1_MURF1 1301 TGTTCTAGCGTATATATACTTTTCTTTGATATTATTAACTTATTTGATCTTATTCTATAACT                                       1362
BF_MURF1       1301 TGTTCTAGCGTATATATACTTTTCTTTGATATTATTAACTTATTTGATCTTATTCTATAACT                                       1362
SM_MURF1       1301 TGTTCTAGCGTATATATACTTTTCTTTGATATTATTAACTTATTTGATCTTATTCTATAACT                                       1362
PF_MURF1       1301 TGTTCTAGCGTATATATACTTTTCTTTGATATTATTAACTTATTTGATCTTATTCTATAACT                                       1362
ref_MURF1      1301 TGTTCTAGCGTATATATACTTTTCTTTGATATTATTAACTTATTTGATCTTATTCTATAACT                                       1362
```

MURF2

```
                                                                                                                       *
29-13_MURF2      1 TTTTATATAGAAAGGTATATAATCTATAATGAAAGGGGATTTTAAGATTGGCTTTGATTGAGTCGTGTTTTTGATTTGTTATGTATTAGAACATATGATT  100
AnTat1.1_MURF2   1 TTTTATATAGAAAGGTATATAATCTATAATGAAAGGGGATTTTAAGATTGGCTTTGATTGAGTCGTGTTTTTGATTTGTTATGTATTAGAACATACGATT  100
BF_MURF2         1 TTTTATATAGAAAGGTATATAATCTATAATGAAAGGGGATTTTAAGATTGGCTTTGATTGAGTCGTGTTTTTGATTTGTTATGTATTAGAACATATGATT  100
SM_MURF2         1 TTTTATATAGAAAGGTATATAATCTATAATGAAAGGGGATTTTAAGATTGGCTTTGATTGAGTCGTGTTTTTGATTTGTTATGTATTAGAACATATGATT  100
PF_MURF2         1 TTTTATATAGAAAGGTATATAATCTATAATGAAAGGGGATTTTAAGATTGGCTTTGATTGAGTCGTGTTTTTGATTTGTTATGTATTAGAACATATGATT  100
ref_MURF2        1 TTTTATATAGAAAGGTATATAATCTATAATGAAAGGGGATTTTAAGATTGGCTTTGATTGAGTCGTGTTTTTGATTTGTTATGTATTAGAACATATGATT  100

29-13_MURF2    101 TTATATTGTGATGGTTTGATCTAGATTTTATATTATATGATTTTGTATTCGATTTTGTTGTATGTATTACATTTATATTTATATTTGTTTTAGGTTTTTT  200
AnTat1.1_MURF2 101 TTATATTGTGATGGTTTGATCTAGATTTTATATTATATGATTTTGTATTCGATTTTGTTGTATGTATTACATTTATATTTATATTTGTTTTAGGTTTTTT  200
BF_MURF2       101 TTATATTGTGATGGTTTGATCTAGATTTTATATTATATGATTTTGTATTCGATTTTGTTGTATGTATTACATTTATATTTATATTTGTTTTAGGTTTTTT  200
SM_MURF2       101 TTATATTGTGATGGTTTGATCTAGATTTTATATTATATGATTTTGTATTCGATTTTGTTGTATGTATTACATTTATATTTATATTTGTTTTAGGTTTTTT  200
PF_MURF2       101 TTATATTGTGATGGTTTGATCTAGATTTTATATTATATGATTTTGTATTCGATTTTGTTGTATGTATTACATTTATATTTATATTTGTTTTAGGTTTTTT  200
ref_MURF2      101 TTATATTGTGATGGTTTGATCTAGATTTTATATTATATGATTTTGTATTCGATTTTGTTGTATGTATTACATTTATATTTATATTTGTTTTAGGTTTTTT  200

29-13_MURF2    201 TATTAGAATTTTTTTTAGTTTTGTGTTTGTATTGTTATTTATAACATTTTTTGGAATTTGTTCATTAACAATGTTATTTACAGGGTATTATATATATTAT  300
AnTat1.1_MURF2 201 TATTAGAATTTTTTTTAGTTTTGTGTTTGTATTGTTATTTATAACATTTTTTGGAATTTGTTCATTAACAATGTTATTTACAGGGTATTATATATATTAT  300
BF_MURF2       201 TATTAGAATTTTTTTTAGTTTTGTGTTTGTATTGTTATTTATAACATTTTTTGGAATTTGTTCATTAACAATGTTATTTACAGGGTATTATATATATTAT  300
SM_MURF2       201 TATTAGAATTTTTTTTAGTTTTGTGTTTGTATTGTTATTTATAACATTTTTTGGAATTTGTTCATTAACAATGTTATTTACAGGGTATTATATATATTAT  300
PF_MURF2       201 TATTAGAATTTTTTTTAGTTTTGTGTTTGTATTGTTATTTATAACATTTTTTGGAATTTGTTCATTAACAATGTTATTTACAGGGTATTATATATATTAT  300
ref_MURF2      201 TATTAGAATTTTTTTTAGTTTTGTGTTTGTATTGTTATTTATAACATTTTTTGGAATTTGTTCATTAACAATGTTATTTACAGGGTATTATATATATTAT  300
                                                                                                                       *
29-13_MURF2    301 ATATATATATTATATAATTTTATATGTTTTTTTTTTGCATTTGGTATAAATTTTTTGATATATTATATCGAGTTTTTCATATTTATAACATTCCATATAT  400
AnTat1.1_MURF2 301 ATATATATATTATATAATTTTATATGTTTTTTTTTTGCATTTGGTATAAATTTTTTGATATATTATATCGAGTTTTTCATATTTATAACATTCCACATAT  400
BF_MURF2       301 ATATATATATTATATAATTTTATATGTTTTTTTTTTGCATTTGGTATAAATTTTTTGATATATTATATCGAGTTTTTCATATTTATAACATTCCATATAT  400
SM_MURF2       301 ATATATATATTATATAATTTTATATGTTTTTTTTTTGCATTTGGTATAAATTTTTTGATATATTATATCGAGTTTTTCATATTTATAACATTCCATATAT  400
PF_MURF2       301 ATATATATATTATATAATTTTATATGTTTTTTTTTTGCATTTGGTATAAATTTTTTGATATATTATATCGAGTTTTTCATATTTATAACATTCCATATAT  400
ref_MURF2      301 ATATATATATTATATAATTTTATATGTTTTTTTTTTGCATTTGGTATAAATTTTTTGATATATTATATCGAGTTTTTCATATTTATAACATTCCATATAT  400

29-13_MURF2    401 TTTTCGATTTTATAAGTTTTTCTAATTATATATATAATTATTTTGGAATATTGTATATGTTTAATGTAATGTTTTGTGCATATTTATTTTGTTTATTTTA  500
AnTat1.1_MURF2 401 TTTTCGATTTTATAAGTTTTTCTAATTATATATATAATTATTTTGGAATATTGTATATGTTTAATGTAATGTTTTGTGCATATTTATTTTGTTTATTTTA  500
BF_MURF2       401 TTTTCGATTTTATAAGTTTTTCTAATTATATATATAATTATTTTGGAATATTGTATATGTTTAATGTAATGTTTTGTGCATATTTATTTTGTTTATTTTA  500
SM_MURF2       401 TTTTCGATTTTATAAGTTTTTCTAATTATATATATAATTATTTTGGAATATTGTATATGTTTAATGTAATGTTTTGTGCATATTTATTTTGTTTATTTTA  500
PF_MURF2       401 TTTTCGATTTTATAAGTTTTTCTAATTATATATATAATTATTTTGGAATATTGTATATGTTTAATGTAATGTTTTGTGCATATTTATTTTGTTTATTTTA  500
ref_MURF2      401 TTTTCGATTTTATAAGTTTTTCTAATTATATATATAATTATTTTGGAATATTGTATATGTTTAATGTAATGTTTTGTGCATATTTATTTTGTTTATTTTA  500

29-13_MURF2    501 TTTTGTGATATATTTTTTATTTTGTTTTATATTTTTTGTAATACGATGTTTATTTATAGTAATAATGGATTTTTTATTTTTTTAATTTTGATATATTTGTA  600
AnTat1.1_MURF2 501 TTTTGTGATATATTTTTTATTTTGTTTTATATTTTTTGTAATACGATGTTTATTTATAGTAATAATGGATTTTTTATTTTTTTAATTTTGATATATTTGTA  600
BF_MURF2       501 TTTTGTGATATATTTTTTATTTTGTTTTATATTTTTTGTAATACGATGTTTATTTATAGTAATAATGGATTTTTTATTTTTTTAATTTTGATATATTTGTA  600
SM_MURF2       501 TTTTGTGATATATTTTTTATTTTGTTTTATATTTTTTGTAATACGATGTTTATTTATAGTAATAATGGATTTTTTATTTTTTTAATTTTGATATATTTGTA  600
PF_MURF2       501 TTTTGTGATATATTTTTTATTTTGTTTTATATTTTTTGTAATACGATGTTTATTTATAGTAATAATGGATTTTTTATTTTTTTAATTTTGATATATTTGTA  600
ref_MURF2      501 TTTTGTGATATATTTTTTATTTTGTTTTATATTTTTTGTAATACGATGTTTATTTATAGTAATAATGGATTTTTTATTTTTTTAATTTTGATATATTTGTA  600

29-13_MURF2    601 TCTATATTATTATGTGATATAGTATATTTAGATTTTATAAGTTTATTATTATTATATTTTAATTTTATATTTAATTTTATTTATGGATTTTTTAGTTTTG  700
AnTat1.1_MURF2 601 TCTATATTATTATGTGATATAGTATATTTAGATTTTATAAGTTTATTATTATTATATTTTAATTTTATATTTAATTTTATTTATGGATTTTTTAGTTTTG  700
BF_MURF2       601 TCTATATTATTATGTGATATAGTATATTTAGATTTTATAAGTTTATTATTATTATATTTTAATTTTATATTTAATTTTATTTATGGATTTTTTAGTTTTG  700
SM_MURF2       601 TCTATATTATTATGTGATATAGTATATTTAGATTTTATAAGTTTATTATTATTATATTTTAATTTTATATTTAATTTTATTTATGGATTTTTTAGTTTTG  700
PF_MURF2       601 TCTATATTATTATGTGATATAGTATATTTAGATTTTATAAGTTTATTATTATTATATTTTAATTTTATATTTAATTTTATTTATGGATTTTTTAGTTTTG  700
ref_MURF2      601 TCTATATTATTATGTGATATAGTATATTTAGATTTTATAAGTTTATTATTATTATATTTTAATTTTATATTTAATTTTATTTATGGATTTTTTAGTTTTG  700

29-13_MURF2    701 TGATAATTTTAGGTTTATTATTTTTTATTGTTATTTTTAGTAATAAATTTATTTTTTTGGATTTACATTTTTAGTATATGGTATACAAATCATATTATTATA  800
AnTat1.1_MURF2 701 TGATAATTTTAGGTTTATTATTTTTTATTGTTATTTTTAGTAATAAATTTATTTTTTTGGATTTACATTTTTAGTATATGGTATACAAATCATATTATTATA  800
BF_MURF2       701 TGATAATTTTAGGTTTATTATTTTTTATTGTTATTTTTAGTAATAAATTTATTTTTTTGGATTTACATTTTTAGTATATGGTATACAAATCATATTATTATA  800
SM_MURF2       701 TGATAATTTTAGGTTTATTATTTTTTATTGTTATTTTTAGTAATAAATTTATTTTTTTGGATTTACATTTTTAGTATATGGTATACAAATCATATTATTATA  800
PF_MURF2       701 TGATAATTTTAGGTTTATTATTTTTTATTGTTATTTTTAGTAATAAATTTATTTTTTTGGATTTACATTTTTAGTATATGGTATACAAATCATATTATTATA  800
ref_MURF2      701 TGATAATTTTAGGTTTATTATTTTTTATTGTTATTTTTAGTAATAAATTTATTTTTTTGGATTTACATTTTTAGTATATGGTATACAAATCATATTATTATA  800

29-13_MURF2    801 TTATGTATATTGATTATATATGATATATAGTAGAAGTTGTTATATATTGATGCCAGCAATATTAATATTTTTTAAGTTTATATATTTTGATGTATTCTTT  900
AnTat1.1_MURF2 801 TTATGTATATTGATTATATATGATATATAGTAGAAGTTGTTATATATTGATGCCAGCAATATTAATATTTTTTAAGTTTATATATTTTGATGTATTCTTT  900
BF_MURF2       801 TTATGTATATTGATTATATATGATATATAGTAGAAGTTGTTATATATTGATGCCAGCAATATTAATATTTTTTAAGTTTATATATTTTGATGTATTCTTT  900
SM_MURF2       801 TTATGTATATTGATTATATATGATATATAGTAGAAGTTGTTATATATTGATGCCAGCAATATTAATATTTTTTAAGTTTATATATTTTGATGTATTCTTT  900
PF_MURF2       801 TTATGTATATTGATTATATATGATATATAGTAGAAGTTGTTATATATTGATGCCAGCAATATTAATATTTTTTAAGTTTATATATTTTGATGTATTCTTT  900
ref_MURF2      801 TTATGTATATTGATTATATATGATATATAGTAGAAGTTGTTATATATTGATGCCAGCAATATTAATATTTTTTAAGTTTATATATTTTGATGTATTCTTT  900
                                                                                                                          *
29-13_MURF2    901 GTGTTTGTATTTATTTTAATTTTATTTATTATATCATTTTTTAGTTTTTTTTTAAAAGATTTTTTATTTTTTATCATTATATTTTGATATATTCGGATCAT 1000
AnTat1.1_MURF2 901 GTGTTTGTATTTATTTTAATTTTATTTATTATATCATTTTTTAGTTTTTTTTTAAAAGATTTTTTATTTTTTATCATTATATTTTGATATATTCGGATCAC 1000
BF_MURF2       901 GTGTTTGTATTTATTTTAATTTTATTTATTATATCATTTTTTAGTTTTTTTTTAAAAGATTTTTTATTTTTTATCATTATATTTTGATATATTCGGATCAT 1000
SM_MURF2       901 GTGTTTGTATTTATTTTAATTTTATTTATTATATCATTTTTTAGTTTTTTTTTAAAAGATTTTTTATTTTTTATCATTATATTTTGATATATTCGGATCAT 1000
PF_MURF2       901 GTGTTTGTATTTATTTTAATTTTATTTATTATATCATTTTTTAGTTTTTTTTTAAAAGATTTTTTATTTTTTATCATTATATTTTGATATATTCGGATCAT 1000
ref_MURF2      901 GTGTTTGTATTTATTTTAATTTTATTTATTATATCATTTTTTAGTTTTTTTTTAAAAGATTTTTTATTTTTTATCATTATATTTTGATATATTCGGATCAT 1000

29-13_MURF2   1001 TATATAATTACGATATATTATCATATAGTATATTTTATTATCAAAATAATCAGTTTTGTTTAACACAGTTATTATCAATTTATATATAAAA  1091
AnTat1.1_MURF2 1001 TATATAATTACGATATATTATCATATAGTATATTTTATTATCAAAATAATCAGTTTTGTTTAACACAGTTATTATCAATTTATATATAAAA  1091
BF_MURF2      1001 TATATAATTACGATATATTATCATATAGTATATTTTATTATCAAAATAATCAGTTTTGTTTAACACAGTTATTATCAATTTATATATAAAA  1091
SM_MURF2      1001 TATATAATTACGATATATTATCATATAGTATATTTTATTATCAAAATAATCAGTTTTGTTTAACACAGTTATTATCAATTTATATATAAAA  1091
PF_MURF2      1001 TATATAATTACGATATATTATCATATAGTATATTTTATTATCAAAATAATCAGTTTTGTTTAACACAGTTATTATCAATTTATATATAAAA  1091
ref_MURF2     1001 TATATAATTACGATATATTATCATATAGTATATTTTATTATCAAAATAATCAGTTTTGTTTAACACAGTTATTATCAATTTATATATAAAA  1091
```

uS3m

```
                               *    ***
29-13_uS3m      1 TCATGTTTTTTTGTATCATATAAATTAATATACAAAAATGTTCCTTATTCATTTTGTGCATTACAAAACCATATTACAAAAATATACATTTAAATTTAAA 100
AnTat1.1_uS3m   1 TCATGTTTTTTT-TATATCATAAATTAATATACAAAAATGTTCCTTATTCATTTTGTGCATTACAAAACCATATTACAAAAATATACATTTAAATTTAAA  99
BF_uS3m         1 TCATGTTTTTTTGTATCATATAAATTAATATACAAAAATGTTCCTTATTCATTTTGTGCATTACAAAACCATATTACAAAAATATACATTTAAATTTAAA 100
SM_uS3m         1 TCATGTTTTTTTGTATCATATAAATTAATATACAAAAATGTTCCTTATTCATTTTGTGCATTACAAAACCATATTACAAAAATATACATTTAAATTTAAA 100
PF_uS3m         1 TCATGTTTTTTTGTATCATATAAATTAATATACAAAAATGTTCCTTATTCATTTTGTGCATTACAAAACCATATTACAAAAATATACATTTAAATTTAAA 100
ref_uS3m        1 TCATGTTTTTTTGTATCATATAAATTAATATACAAAAATGTTCCTTATTCATTTTGTGCATTACAAAACCATATTACAAAAATATACATTTAAATTTAAA 100
                                                   *                                  *
29-13_uS3m    101 CATATTTTTTTGTCTATTGATAAATACAATAGCCTGTTTTTTAACATATCTGGCATTTTAATTTGACTTAACATTATACACATTAATATTATACTAATAA 200
AnTat1.1_uS3m 100 CATATTTTTTTGTCTATTGATAAATACAATAGTCTGTTTTTTAACATATCTGGCATTTTAATTTGACTGAACATTATACACATTAATATTATACTAATAA 199
BF_uS3m       101 CATATTTTTTTGTCTATTGATAAATACAATAGCCTGTTTTTTAACATATCTGGCATTTTAATTTGACTTAACATTATACACATTAATATTATACTAATAA 200
SM_uS3m       101 CATATTTTTTTGTCTATTGATAAATACAATAGCCTGTTTTTTAACATATCTGGCATTTTAATTTGACTTAACATTATACACATTAATATTATACTAATAA 200
PF_uS3m       101 CATATTTTTTTGTCTATTGATAAATACAATAGCCTGTTTTTTAACATATCTGGCATTTTAATTTGACTTAACATTATACACATTAATATTATACTAATAA 200
ref_uS3m      101 CATATTTTTTTGTCTATTGATAAATACAATAGCCTGTTTTTTAACATATCTGGCATTTTAATTTGACTTAACATTATACACATTAATATTATACTAATAA 200
                                                             *  ***********
29-13_uS3m    201 AATATTCTTTCTTTATATTAATAAATAATTTTGAATATTTAATAATATTAATATCAACTTAA                                      262
AnTat1.1_uS3m 200 AATATTCTTTCTTTATATTAATAAATAATTTTGAATATTTAATAATATCAA-----------                                      250
BF_uS3m       201 AATATTCTTTCTTTATATTAATAAATAATTTTGAATATTTAATAATATTAATATCAACTTAA                                      262
SM_uS3m       201 AATATTCTTTCTTTATATTAATAAATAATTTTGAATATTTAATAATATTAATATCAACTTAA                                      262
PF_uS3m       201 AATATTCTTTCTTTATATTAATAAATAATTTTGAATATTTAATAATATTAATATCAACTTAA                                      262
ref_uS3m      201 AATATTCTTTCTTTATATTAATAAATAATTTTGAATATTTAATAATATTAATATCAACTTAA                                      262
```

ND1

```
29-13_ND1      1 ATATCATTCAAAAAGTTAATATAAAACTCATTTGTTATTATTACATTTAGATATATGCATACTTATTGTTATATTTATACTCGTTTTATCTGTCTTATGT 100
AnTat1.1_ND1   1 ATATCATTCAAAAAGTTAATATAAAACTCATTTGTTATTATTACATTTAGATATATGCATACTTATTGTTATATTTATACTCGTTTTATCTGTCTTATGT 100
BF_ND1         1 ATATCATTCAAAAAGTTAATATAAAACTCATTTGTTATTATTACATTTAGATATATGCATACTTATTGTTATATTTATACTCGTTTTATCTGTCTTATGT 100
SM_ND1         1 ATATCATTCAAAAAGTTAATATAAAACTCATTTGTTATTATTACATTTAGATATATGCATACTTATTGTTATATTTATACTCGTTTTATCTGTCTTATGT 100
PF_ND1         1 ATATCATTCAAAAAGTTAATATAAAACTCATTTGTTATTATTACATTTAGATATATGCATACTTATTGTTATATTTATACTCGTTTTATCTGTCTTATGT 100
ref_ND1        1 ATATCATTCAAAAAGTTAATATAAAACTCATTTGTTATTATTACATTTAGATATATGCATACTTATTGTTATATTTATACTCGTTTTATCTGTCTTATGT 100

                                                                 *                           *
29-13_ND1    101 GGGTATGTTAGTTTATGTGAACGTAAAATTTTAGCTATTGTGCAATTCAGAATAGGACCTGCACTTTTTTTATTTGGTCTTCTTACTCCTATTACTGATG 200
AnTat1.1_ND1 101 GGGTATGTTAGTTTATGTGAACGTAAAATTTTAGCTATTGTGCAATTTAGAATAGGACCTGCACTTTTTTTATTCGGTCTTCTTACTCCTATTACTGATG 200
BF_ND1       101 GGGTATGTTAGTTTATGTGAACGTAAAATTTTAGCTATTGTGCAATTCAGAATAGGACCTGCACTTTTTTTATTTGGTCTTCTTACTCCTATTACTGATG 200
SM_ND1       101 GGGTATGTTAGTTTATGTGAACGTAAAATTTTAGCTATTGTGCAATTCAGAATAGGACCTGCACTTTTTTTATTTGGTCTTCTTACTCCTATTACTGATG 200
PF_ND1       101 GGGTATGTTAGTTTATGTGAACGTAAAATTTTAGCTATTGTGCAATTCAGAATAGGACCTGCACTTTTTTTATTTGGTCTTCTTACTCCTATTACTGATG 200
ref_ND1      101 GGGTATGTTAGTTTATGTGAACGTAAAATTTTAGCTATTGTGCAATTCAGAATAGGACCTGCACTTTTTTTATTTGGTCTTCTTACTCCTATTACTGATG 200

                                                                                                 * *
29-13_ND1    201 GAGTTAAATTATTTGTTAAATTTACATTATTTGTGATTGGTGTTGACTCTATATTATTTATATCTAGTTTATTCATTACAGCTTTTTGTATTTTTTTTCC 300
AnTat1.1_ND1 201 GAGTTAAATTATTTGTTAAATTTACATTATTTGTGATTGGTGTTGACTCTATATTATTTATATCTAGTTTATTCATTACGGTTTTTTGTATTTTTTTTCC 300
BF_ND1       201 GAGTTAAATTATTTGTTAAATTTACATTATTTGTGATTGGTGTTGACTCTATATTATTTATATCTAGTTTATTCATTACAGCTTTTTGTATTTTTTTTCC 300
SM_ND1       201 GAGTTAAATTATTTGTTAAATTTACATTATTTGTGATTGGTGTTGACTCTATATTATTTATATCTAGTTTATTCATTACAGCTTTTTGTATTTTTTTTCC 300
PF_ND1       201 GAGTTAAATTATTTGTTAAATTTACATTATTTGTGATTGGTGTTGACTCTATATTATTTATATCTAGTTTATTCATTACAGCTTTTTGTATTTTTTTTCC 300
ref_ND1      201 GAGTTAAATTATTTGTTAAATTTACATTATTTGTGATTGGTGTTGACTCTATATTATTTATATCTAGTTTATTCATTACAGCTTTTTGTATTTTTTTTCC 300

29-13_ND1    301 TTGGTTCTTTTTCCCACTTGGATTCATAATAATATTTGACAAAGGTTTTACACTTCTTTTTTTATTAGGATTTCATTTATTTTCTAATGTATTTTGCATT 400
AnTat1.1_ND1 301 TTGGTTCTTTTTCCCACTTGGATTCATAATAATATTTGACAAAGGTTTTACACTTCTTTTTTTATTAGGATTTCATTTATTTTCTAATGTATTTTGCATT 400
BF_ND1       301 TTGGTTCTTTTTCCCACTTGGATTCATAATAATATTTGACAAAGGTTTTACACTTCTTTTTTTATTAGGATTTCATTTATTTTCTAATGTATTTTGCATT 400
SM_ND1       301 TTGGTTCTTTTTCCCACTTGGATTCATAATAATATTTGACAAAGGTTTTACACTTCTTTTTTTATTAGGATTTCATTTATTTTCTAATGTATTTTGCATT 400
PF_ND1       301 TTGGTTCTTTTTCCCACTTGGATTCATAATAATATTTGACAAAGGTTTTACACTTCTTTTTTTATTAGGATTTCATTTATTTTCTAATGTATTTTGCATT 400
ref_ND1      301 TTGGTTCTTTTTCCCACTTGGATTCATAATAATATTTGACAAAGGTTTTACACTTCTTTTTTTATTAGGATTTCATTTATTTTCTAATGTATTTTGCATT 400

29-13_ND1    401 TTTTTCGTTGGTTGTTTTTTATTTTCAAGCTGCTTTATTTACCTAGCTGCCATGAGAACTTTATTTTTTAGTATTCTATCTGAATGTTCCATTTTAATTC 500
AnTat1.1_ND1 401 TTTTTCGTTGGTTGTTTTTTATTTTCAAGCTGCTTTATTTACCTAGCTGCCATGAGAACTTTATTTTTTAGTATTCTATCTGAATGTTCCATTTTAATTC 500
BF_ND1       401 TTTTTCGTTGGTTGTTTTTTATTTTCAAGCTGCTTTATTTACCTAGCTGCCATGAGAACTTTATTTTTTAGTATTCTATCTGAATGTTCCATTTTAATTC 500
SM_ND1       401 TTTTTCGTTGGTTGTTTTTTATTTTCAAGCTGCTTTATTTACCTAGCTGCCATGAGAACTTTATTTTTTAGTATTCTATCTGAATGTTCCATTTTAATTC 500
PF_ND1       401 TTTTTCGTTGGTTGTTTTTTATTTTCAAGCTGCTTTATTTACCTAGCTGCCATGAGAACTTTATTTTTTAGTATTCTATCTGAATGTTCCATTTTAATTC 500
ref_ND1      401 TTTTTCGTTGGTTGTTTTTTATTTTCAAGCTGCTTTATTTACCTAGCTGCCATGAGAACTTTATTTTTTAGTATTCTATCTGAATGTTCCATTTTAATTC 500

29-13_ND1    501 TACTTTATTGTATATACATACTAGACTACTTTTGTTTCTTTGGCATAAAAGATATTTGTATAAGTCAACTTTCTTTACAAAATTGCTTTATACTTGGTCT 600
AnTat1.1_ND1 501 TACTTTATTGTATATACATACTAGACTACTTTTGTTTCTTTGGCATAAAAGATATTTGTATAAGTCAACTTTCTTTACAAAATTGCTTTATACTTGGTCT 600
BF_ND1       501 TACTTTATTGTATATACATACTAGACTACTTTTGTTTCTTTGGCATAAAAGATATTTGTATAAGTCAACTTTCTTTACAAAATTGCTTTATACTTGGTCT 600
SM_ND1       501 TACTTTATTGTATATACATACTAGACTACTTTTGTTTCTTTGGCATAAAAGATATTTGTATAAGTCAACTTTCTTTACAAAATTGCTTTATACTTGGTCT 600
PF_ND1       501 TACTTTATTGTATATACATACTAGACTACTTTTGTTTCTTTGGCATAAAAGATATTTGTATAAGTCAACTTTCTTTACAAAATTGCTTTATACTTGGTCT 600
ref_ND1      501 TACTTTATTGTATATACATACTAGACTACTTTTGTTTCTTTGGCATAAAAGATATTTGTATAAGTCAACTTTCTTTACAAAATTGCTTTATACTTGGTCT 600

                                                *                                       *                       *
29-13_ND1    601 TTTATTCATTTGTCTATTTTGAATTGGACTGCTTCTTGATGGATTACGTTTACCCTTTGATTATCTAGAATGTGAAAGTGAACTTGTTGCTGGCCTTGTT 700
AnTat1.1_ND1 601 TTTATTCATTTGTCTATTTTGAATTGGACTACTTCTTGATGGATTACGTTTACCCTTTGATTATCTAGAGTGTGAAAGTGAACTTGTTGCTGGTCTTGTT 700
BF_ND1       601 TTTATTCATTTGTCTATTTTGAATTGGACTGCTTCTTGATGGATTACGTTTACCCTTTGATTATCTAGAATGTGAAAGTGAACTTGTTGCTGGCCTTGTT 700
SM_ND1       601 TTTATTCATTTGTCTATTTTGAATTGGACTGCTTCTTGATGGATTACGTTTACCCTTTGATTATCTAGAATGTGAAAGTGAACTTGTTGCTGGCCTTGTT 700
PF_ND1       601 TTTATTCATTTGTCTATTTTGAATTGGACTGCTTCTTGATGGATTACGTTTACCCTTTGATTATCTAGAATGTGAAAGTGAACTTGTTGCTGGCCTTGTT 700
ref_ND1      601 TTTATTCATTTGTCTATTTTGAATTGGACTGCTTCTTGATGGATTACGTTTACCCTTTGATTATCTAGAATGTGAAAGTGAACTTGTTGCTGGCCTTGTT 700

                      *         *                     *                         *
29-13_ND1    701 ACTGAATTATCTGGTATTTTTTTTGTCATATACTCCGTCCTTGAAATTAATCATTTATTACTAACTACTATATTATTTAGCTGCTTATGTTTTGGTGGCC 800
AnTat1.1_ND1 701 ACTGAGTTATCTGGTGTTTTTTTTGTCATATACTCCATCCTTGAAATTAATCATTTATTATTAACTACTATATTATTTAGCTGCTTATGTTTTGGTGGCC 800
BF_ND1       701 ACTGAATTATCTGGTATTTTTTTTGTCATATACTCCGTCCTTGAAATTAATCATTTATTACTAACTACTATATTATTTAGCTGCTTATGTTTTGGTGGCC 800
SM_ND1       701 ACTGAATTATCTGGTATTTTTTTTGTCATATACTCCGTCCTTGAAATTAATCATTTATTACTAACTACTATATTATTTAGCTGCTTATGTTTTGGTGGCC 800
PF_ND1       701 ACTGAATTATCTGGTATTTTTTTTGTCATATACTCCGTCCTTGAAATTAATCATTTATTACTAACTACTATATTATTTAGCTGCTTATGTTTTGGTGGCC 800
ref_ND1      701 ACTGAATTATCTGGTATTTTTTTTGTCATATACTCCGTCCTTGAAATTAATCATTTATTACTAACTACTATATTATTTAGCTGCTTATGTTTTGGTGGCC 800

29-13_ND1    801 TTTTTATATGCTTTAAATCTATTCTTATACTAATTCTAGGTTTTCTTATACCACGTGTTATATGCTGTAGATTAAAAATTACAACTGCACAAACATTTAT 900
AnTat1.1_ND1 801 TTTTTATATGCTTTAAATCTATTCTTATACTAATTCTAGGTTTTCTTATACCACGTGTTATATGCTGTAGATTAAAAATTACAACTGCACAAACATTTAT 900
BF_ND1       801 TTTTTATATGCTTTAAATCTATTCTTATACTAATTCTAGGTTTTCTTATACCACGTGTTATATGCTGTAGATTAAAAATTACAACTGCACAAACATTTAT 900
SM_ND1       801 TTTTTATATGCTTTAAATCTATTCTTATACTAATTCTAGGTTTTCTTATACCACGTGTTATATGCTGTAGATTAAAAATTACAACTGCACAAACATTTAT 900
PF_ND1       801 TTTTTATATGCTTTAAATCTATTCTTATACTAATTCTAGGTTTTCTTATACCACGTGTTATATGCTGTAGATTAAAAATTACAACTGCACAAACATTTAT 900
ref_ND1      801 TTTTTATATGCTTTAAATCTATTCTTATACTAATTCTAGGTTTTCTTATACCACGTGTTATATGCTGTAGATTAAAAATTACAACTGCACAAACATTTAT 900

29-13_ND1    901 ATTACTTTTTTTATTTACAATGGGATTCATTAATTTTTCATTTATTGCTATTACAAAAATTATTTGCATACTTTTTTAA                      979
AnTat1.1_ND1 901 ATTACTTTTTTTATTTACAATGGGATTCATTAATTTTTCATTTATTGCTATTACAAAAATTATTTGCATACTTTTTTAA                      979
BF_ND1       901 ATTACTTTTTTTATTTACAATGGGATTCATTAATTTTTCATTTATTGCTATTACAAAAATTATTTGCATACTTTTTTAA                      979
SM_ND1       901 ATTACTTTTTTTATTTACAATGGGATTCATTAATTTTTCATTTATTGCTATTACAAAAATTATTTGCATACTTTTTTAA                      979
PF_ND1       901 ATTACTTTTTTTATTTACAATGGGATTCATTAATTTTTCATTTATTGCTATTACAAAAATTATTTGCATACTTTTTTAA                      979
ref_ND1      901 ATTACTTTTTTTATTTACAATGGGATTCATTAATTTTTCATTTATTGCTATTACAAAAATTATTTGCATACTTTTTTAA                      979
```

ND3

```
                                                                                     *            *
29-13_ND3      1 TCAAAAAATCCTCGCCTTTTTACTTTAGTTTGTTATCAAAAGTGTATTAGGGATTTTAAAAGGAGAGAGCAGAGCGGGGGGCGGGGTTGAGAAGAATCAC 100
AnTat1.1_ND3   1 TCAAAAAATCCTCGCCTTTTTACTTTAGTTTGTTATCAAAAGTGTATTAGGGATTTTAAAAGGAGAGGGCAGAGCGGGGGACGGGGTTGAGAAGAATCAC 100
BF_ND3         1 TCAAAAAATCCTCGCCTTTTTACTTTAGTTTGTTATCAAAAGTGTATTAGGGATTTTAAAAGGAGAGAGCAGAGCGGGGGGCGGGGTTGAGAAGAATCAC 100
SM_ND3         1 TCAAAAAATCCTCGCCTTTTTACTTTAGTTTGTTATCAAAAGTGTATTAGGGATTTTAAAAGGAGAGAGCAGAGCGGGGGGCGGGGTTGAGAAGAATCAC 100
PF_ND3         1 TCAAAAAATCCTCGCCTTTTTACTTTAGTTTGTTATCAAAAGTGTATTAGGGATTTTAAAAGGAGAGAGCAGAGCGGGGGGCGGGGTTGAGAAGAATCAC 100
ref_ND3        1 TCAAAAAATCCTCGCCTTTTTACTTTAGTTTGTTATCAAAAGTGTATTAGGGATTTTAAAAGGAGAGAGCAGAGCGGGGGGCGGGGTTGAGAAGAATCAC 100

                                                           *
29-13_ND3    101 GGTGTGAACAGGAATGGAGTGTTGGAGAAACCATAGGTTTGGGGTGAATGGGAGATGGGTTTTGGGGGGAGAAGTTTTGATGGGGAGGGATCACTGAAGG 200
AnTat1.1_ND3 101 GGTGTGAACAGGAATGGAGTGTTGGAGAAACCATAGGTTTGAGGTGAATGGGAGATGGGTTTTGGGGGGAGAAGTTTTGATGGGGAGGGATCACTGAAGG 200
BF_ND3       101 GGTGTGAACAGGAATGGAGTGTTGGAGAAACCATAGGTTTGGGGTGAATGGGAGATGGGTTTTGGGGGGAGAAGTTTTGATGGGGAGGGATCACTGAAGG 200
SM_ND3       101 GGTGTGAACAGGAATGGAGTGTTGGAGAAACCATAGGTTTGGGGTGAATGGGAGATGGGTTTTGGGGGGAGAAGTTTTGATGGGGAGGGATCACTGAAGG 200
PF_ND3       101 GGTGTGAACAGGAATGGAGTGTTGGAGAAACCATAGGTTTGGGGTGAATGGGAGATGGGTTTTGGGGGGAGAAGTTTTGATGGGGAGGGATCACTGAAGG 200
ref_ND3      101 GGTGTGAACAGGAATGGAGTGTTGGAGAAACCATAGGTTTGGGGTGAATGGGAGATGGGTTTTGGGGGGAGAAGTTTTGATGGGGAGGGATCACTGAAGG 200

                                                   *
29-13_ND3    201 GGGTAAAGAGATTTGTTTGTTGTATTATACATA-TTATATTAATAAATATATAAAATAATAAAATACTA                                268
AnTat1.1_ND3 201 GGGTAAAGAGATTTGTTTGTTGTATTATACATATTTATATTAATAAATATATAAAATAATAAAATACTA                                269
BF_ND3       201 GGGTAAAGAGATTTGTTTGTTGTATTATACATA-TTATATTAATAAATATATAAAATAATAAAATACTA                                268
SM_ND3       201 GGGTAAAGAGATTTGTTTGTTGTATTATACATA-TTATATTAATAAATATATAAAATAATAAAATACTA                                268
PF_ND3       201 GGGTAAAGAGATTTGTTTGTTGTATTATACATA-TTATATTAATAAATATATAAAATAATAAAATACTA                                268
ref_ND3      201 GGGTAAAGAGATTTGTTTGTTGTATTATACATA-TTATATTAATAAATATATAAAATAATAAAATACTA                                268
```

ND4

```
                        *                                           *
29-13_ND4         1 AAGGAAATTTATAGAAAGCACAAAAATAAAATTAAATTAGAGTAATTGAATGTTAAAATTAAATTTAATATGTATAAATTTTATATTGTTAATTGTTACA  100
AnTat1.1_ND4      1 AAGGTAATTTATAGAAAGCACAAAAATAAAATTAAATTAGAGTAATTGGATGTTAAAATTAAATTTAATATGTATAAATTTTATATTGTTAATTGTTACA  100
BF_ND4            1 AAGGAAATTTATAGAAAGCACAAAAATAAAATTAAATTAGAGTAATTGAATGTTAAAATTAAATTTAATATGTATAAATTTTATATTGTTAATTGTTACA  100
SM_ND4            1 AAGGAAATTTATAGAAAGCACAAAAATAAAATTAAATTAGAGTAATTGAATGTTAAAATTAAATTTAATATGTATAAATTTTATATTGTTAATTGTTACA  100
PF_ND4            1 AAGGAAATTTATAGAAAGCACAAAAATAAAATTAAATTAGAGTAATTGAATGTTAAAATTAAATTTAATATGTATAAATTTTATATTGTTAATTGTTACA  100
ref_ND4           1 AAGGAAATTTATAGAAAGCACAAAAATAAAATTAAATTAGAGTAATTGAATGTTAAAATTAAATTTAATATGTATAAATTTTATATTGTTAATTGTTACA  100

                                                                                                           *
29-13_ND4       101 ATAATATATATATATATAAACTATAGTTTTTGTATTGGAATAGAAATCAATTATGTATATGTAAATATATATTTAAATTACATCAGTCTATGATTTGTAT  200
AnTat1.1_ND4    101 ATAATATATATATATATAAACTATAGTTTTTGTATTGGAATAGAAATCAATTATGTATATGTAAATATATATTTAAATTACATCAGTTTATGATTTGTAT  200
BF_ND4          101 ATAATATATATATATATAAACTATAGTTTTTGTATTGGAATAGAAATCAATTATGTATATGTAAATATATATTTAAATTACATCAGTCTATGATTTGTAT  200
SM_ND4          101 ATAATATATATATATATAAACTATAGTTTTTGTATTGGAATAGAAATCAATTATGTATATGTAAATATATATTTAAATTACATCAGTCTATGATTTGTAT  200
PF_ND4          101 ATAATATATATATATATAAACTATAGTTTTTGTATTGGAATAGAAATCAATTATGTATATGTAAATATATATTTAAATTACATCAGTCTATGATTTGTAT  200
ref_ND4         101 ATAATATATATATATATAAACTATAGTTTTTGTATTGGAATAGAAATCAATTATGTATATGTAAATATATATTTAAATTACATCAGTCTATGATTTGTAT  200

29-13_ND4       201 TTTTTATGGGAATTATTATGTACATATTAATATTTTTATTATCAAAGAAATGTGTATCATATAATAAATATTTTTACATAGTAATGATATATATGTATAT  300
AnTat1.1_ND4    201 TTTTTATGGGAATTATTATGTACATATTAATATTTTTATTATCAAAGAAATGTGTATCATATAATAAATATTTTTACATAGTAATGATATATATGTATAT  300
BF_ND4          201 TTTTTATGGGAATTATTATGTACATATTAATATTTTTATTATCAAAGAAATGTGTATCATATAATAAATATTTTTACATAGTAATGATATATATGTATAT  300
SM_ND4          201 TTTTTATGGGAATTATTATGTACATATTAATATTTTTATTATCAAAGAAATGTGTATCATATAATAAATATTTTTACATAGTAATGATATATATGTATAT  300
PF_ND4          201 TTTTTATGGGAATTATTATGTACATATTAATATTTTTATTATCAAAGAAATGTGTATCATATAATAAATATTTTTACATAGTAATGATATATATGTATAT  300
ref_ND4         201 TTTTTATGGGAATTATTATGTACATATTAATATTTTTATTATCAAAGAAATGTGTATCATATAATAAATATTTTTACATAGTAATGATATATATGTATAT  300

29-13_ND4       301 ATATATTAATGTAGTGTTAATAATAATATTAGATGATTTTATGTGTTTTATGATAGCCTTCGAAAGTCTATTTTTCCCTATATGTCTAGTAAGTTTATTT  400
AnTat1.1_ND4    301 ATATATTAATGTAGTGTTAATAATAATATTAGATGATTTTATGTGTTTTATGATAGCCTTCGAAAGTCTATTTTTCCCTATATGTCTAGTAAGTTTATTT  400
BF_ND4          301 ATATATTAATGTAGTGTTAATAATAATATTAGATGATTTTATGTGTTTTATGATAGCCTTCGAAAGTCTATTTTTCCCTATATGTCTAGTAAGTTTATTT  400
SM_ND4          301 ATATATTAATGTAGTGTTAATAATAATATTAGATGATTTTATGTGTTTTATGATAGCCTTCGAAAGTCTATTTTTCCCTATATGTCTAGTAAGTTTATTT  400
PF_ND4          301 ATATATTAATGTAGTGTTAATAATAATATTAGATGATTTTATGTGTTTTATGATAGCCTTCGAAAGTCTATTTTTCCCTATATGTCTAGTAAGTTTATTT  400
ref_ND4         301 ATATATTAATGTAGTGTTAATAATAATATTAGATGATTTTATGTGTTTTATGATAGCCTTCGAAAGTCTATTTTTCCCTATATGTCTAGTAAGTTTATTT  400

29-13_ND4       401 TTTAATTTTAATAATAGATTTATTTTTGCTATATTCTATCTTATAATATTTAGTTCAGTTAGTTCAGTGGTATGTATAATTATATGTATAATAGTAATAT  500
AnTat1.1_ND4    401 TTTAATTTTAATAATAGATTTATTTTTGCTATATTCTATCTTATAATATTTAGTTCAGTTAGTTCAGTGGTATGTATAATTATATGTATAATAGTAATAT  500
BF_ND4          401 TTTAATTTTAATAATAGATTTATTTTTGCTATATTCTATCTTATAATATTTAGTTCAGTTAGTTCAGTGGTATGTATAATTATATGTATAATAGTAATAT  500
SM_ND4          401 TTTAATTTTAATAATAGATTTATTTTTGCTATATTCTATCTTATAATATTTAGTTCAGTTAGTTCAGTGGTATGTATAATTATATGTATAATAGTAATAT  500
PF_ND4          401 TTTAATTTTAATAATAGATTTATTTTTGCTATATTCTATCTTATAATATTTAGTTCAGTTAGTTCAGTGGTATGTATAATTATATGTATAATAGTAATAT  500
ref_ND4         401 TTTAATTTTAATAATAGATTTATTTTTGCTATATTCTATCTTATAATATTTAGTTCAGTTAGTTCAGTGGTATGTATAATTATATGTATAATAGTAATAT  500

29-13_ND4       501 CTCATTTCAACATTATAAATTTACAGGCTTTTATTGATGTATGTTATTTTGATAGTTTGTATTCGGCAATTTTTATATGAATATTATTATTTATAATGTT  600
AnTat1.1_ND4    501 CTCATTTCAACATTATAAATTTACAGGCTTTTATTGATGTATGTTATTTTGATAGTTTGTATTCGGCAATTTTTATATGAATATTATTATTTATAATGTT  600
BF_ND4          501 CTCATTTCAACATTATAAATTTACAGGCTTTTATTGATGTATGTTATTTTGATAGTTTGTATTCGGCAATTTTTATATGAATATTATTATTTATAATGTT  600
SM_ND4          501 CTCATTTCAACATTATAAATTTACAGGCTTTTATTGATGTATGTTATTTTGATAGTTTGTATTCGGCAATTTTTATATGAATATTATTATTTATAATGTT  600
PF_ND4          501 CTCATTTCAACATTATAAATTTACAGGCTTTTATTGATGTATGTTATTTTGATAGTTTGTATTCGGCAATTTTTATATGAATATTATTATTTATAATGTT  600
ref_ND4         501 CTCATTTCAACATTATAAATTTACAGGCTTTTATTGATGTATGTTATTTTGATAGTTTGTATTCGGCAATTTTTATATGAATATTATTATTTATAATGTT  600

29-13_ND4       601 CGCTATAAAATACCCAATCTGACCATTCCATGTGTGACTACCAGAGATGCATGTAGAGGTAAATACAGAAATGAGTGTTTTATTAGCAAGTATTGTGCTG  700
AnTat1.1_ND4    601 CGCTATAAAATACCCAATCTGACCATTCCATGTGTGACTACCAGAGATGCATGTAGAGGTAAATACAGAAATGAGTGTTTTATTAGCAAGTATTGTGCTG  700
BF_ND4          601 CGCTATAAAATACCCAATCTGACCATTCCATGTGTGACTACCAGAGATGCATGTAGAGGTAAATACAGAAATGAGTGTTTTATTAGCAAGTATTGTGCTG  700
SM_ND4          601 CGCTATAAAATACCCAATCTGACCATTCCATGTGTGACTACCAGAGATGCATGTAGAGGTAAATACAGAAATGAGTGTTTTATTAGCAAGTATTGTGCTG  700
PF_ND4          601 CGCTATAAAATACCCAATCTGACCATTCCATGTGTGACTACCAGAGATGCATGTAGAGGTAAATACAGAAATGAGTGTTTTATTAGCAAGTATTGTGCTG  700
ref_ND4         601 CGCTATAAAATACCCAATCTGACCATTCCATGTGTGACTACCAGAGATGCATGTAGAGGTAAATACAGAAATGAGTGTTTTATTAGCAAGTATTGTGCTG  700

                                                                                                                 *
29-13_ND4       701 AAAATAGGTTTTTTTGGTGTATACAAATTTTTATTTATCGCATTTAATACGATATCAATATGATTTTTAGGTTTTATAGATAGTGTAATTGTGTTGGGTT  800
AnTat1.1_ND4    701 AAAATAGGTTTTTTTGGTGTATACAAATTTTTATTTATCGCATTTAATACGATATCAATATGATTTTTAGGTTTTATAGATAGTGTAATTGTGCTGGGTT  800
BF_ND4          701 AAAATAGGTTTTTTTGGTGTATACAAATTTTTATTTATCGCATTTAATACGATATCAATATGATTTTTAGGTTTTATAGATAGTGTAATTGTGTTGGGTT  800
SM_ND4          701 AAAATAGGTTTTTTTGGTGTATACAAATTTTTATTTATCGCATTTAATACGATATCAATATGATTTTTAGGTTTTATAGATAGTGTAATTGTGTTGGGTT  800
PF_ND4          701 AAAATAGGTTTTTTTGGTGTATACAAATTTTTATTTATCGCATTTAATACGATATCAATATGATTTTTAGGTTTTATAGATAGTGTAATTGTGTTGGGTT  800
ref_ND4         701 AAAATAGGTTTTTTTGGTGTATACAAATTTTTATTTATCGCATTTAATACGATATCAATATGATTTTTAGGTTTTATAGATAGTGTAATTGTGTTGGGTT  800

29-13_ND4       801 TAGTATTTATAGCAATGTCACTAATATTTTTATCAGACTACAAGAAAATAATAGCGAATTGATCAATAATACACACGGGTATAGGATTAATATTATTATG  900
AnTat1.1_ND4    801 TAGTATTTATAGCAATGTCACTAATATTTTTATCAGACTACAAGAAAATAATAGCGAATTGATCAATAATACACACGGGTATAGGATTAATATTATTATG  900
BF_ND4          801 TAGTATTTATAGCAATGTCACTAATATTTTTATCAGACTACAAGAAAATAATAGCGAATTGATCAATAATACACACGGGTATAGGATTAATATTATTATG  900
SM_ND4          801 TAGTATTTATAGCAATGTCACTAATATTTTTATCAGACTACAAGAAAATAATAGCGAATTGATCAATAATACACACGGGTATAGGATTAATATTATTATG  900
PF_ND4          801 TAGTATTTATAGCAATGTCACTAATATTTTTATCAGACTACAAGAAAATAATAGCGAATTGATCAATAATACACACGGGTATAGGATTAATATTATTATG  900
ref_ND4         801 TAGTATTTATAGCAATGTCACTAATATTTTTATCAGACTACAAGAAAATAATAGCGAATTGATCAATAATACACACGGGTATAGGATTAATATTATTATG  900

                          *
29-13_ND4       901 ACATAATGACATTTTGTTTGTAGGTTTACTAATATTATGTAATCTAGCACATATACTAAGTTCATCCTTTATGTTTATTGTAATAGGATATATGTACGAC 1000
AnTat1.1_ND4    901 ACATAACGACATTTTGTTTGTAGGTTTACTAATATTATGTAATCTAGCACATATACTAAGTTCATCCTTTATGTTTATTGTAATAGGATATATGTACGAC 1000
BF_ND4          901 ACATAATGACATTTTGTTTGTAGGTTTACTAATATTATGTAATCTAGCACATATACTAAGTTCATCCTTTATGTTTATTGTAATAGGATATATGTACGAC 1000
SM_ND4          901 ACATAATGACATTTTGTTTGTAGGTTTACTAATATTATGTAATCTAGCACATATACTAAGTTCATCCTTTATGTTTATTGTAATAGGATATATGTACGAC 1000
PF_ND4          901 ACATAATGACATTTTGTTTGTAGGTTTACTAATATTATGTAATCTAGCACATATACTAAGTTCATCCTTTATGTTTATTGTAATAGGATATATGTACGAC 1000
ref_ND4         901 ACATAATGACATTTTGTTTGTAGGTTTACTAATATTATGTAATCTAGCACATATACTAAGTTCATCCTTTATGTTTATTGTAATAGGATATATGTACGAC 1000

                                                                                     *
29-13_ND4      1001 AATTATGGTGTAAGAATTTTTTTATTGTTAATTTCATTTTTTGGTATTAGTATATGAAGTTCATTGTTTTTATGTTTATTTTTATTTAATATAGATTTCC 1100
AnTat1.1_ND4   1001 AATTATGGTGTAAGAATTTTTTTATTGTTAATTTCATTTTTTGGTATTAGTATATGAAGTTCATTATTTTTATGTTTATTTTTATTTAATATAGATTTCC 1100
BF_ND4         1001 AATTATGGTGTAAGAATTTTTTTATTGTTAATTTCATTTTTTGGTATTAGTATATGAAGTTCATTGTTTTTATGTTTATTTTTATTTAATATAGATTTCC 1100
SM_ND4         1001 AATTATGGTGTAAGAATTTTTTTATTGTTAATTTCATTTTTTGGTATTAGTATATGAAGTTCATTGTTTTTATGTTTATTTTTATTTAATATAGATTTCC 1100
PF_ND4         1001 AATTATGGTGTAAGAATTTTTTTATTGTTAATTTCATTTTTTGGTATTAGTATATGAAGTTCATTGTTTTTATGTTTATTTTTATTTAATATAGATTTCC 1100
ref_ND4        1001 AATTATGGTGTAAGAATTTTTTTATTGTTAATTTCATTTTTTGGTATTAGTATATGAAGTTCATTGTTTTTATGTTTATTTTTATTTAATATAGATTTCC 1100

29-13_ND4      1101 CGTTTATGTTATTATTTTATGTAGATATATTTATTTTGTATGGTTTGATATCTATATCATTTATATATATAATAAGTTTTTATATAATAACTTTAACGAT 1200
AnTat1.1_ND4   1101 CGTTTATGTTATTATTTTATGTAGATATATTTATTTTGTATGGTTTGATATCTATATCATTTATATATATAATAAGTTTTTATATAATAACTTTAACGAT 1200
BF_ND4         1101 CGTTTATGTTATTATTTTATGTAGATATATTTATTTTGTATGGTTTGATATCTATATCATTTATATATATAATAAGTTTTTATATAATAACTTTAACGAT 1200
SM_ND4         1101 CGTTTATGTTATTATTTTATGTAGATATATTTATTTTGTATGGTTTGATATCTATATCATTTATATATATAATAAGTTTTTATATAATAACTTTAACGAT 1200
PF_ND4         1101 CGTTTATGTTATTATTTTATGTAGATATATTTATTTTGTATGGTTTGATATCTATATCATTTATATATATAATAAGTTTTTATATAATAACTTTAACGAT 1200
ref_ND4        1101 CGTTTATGTTATTATTTTATGTAGATATATTTATTTTGTATGGTTTGATATCTATATCATTTATATATATAATAAGTTTTTATATAATAACTTTAACGAT 1200

29-13_ND4      1201 ATTTTTATCATCAATATACATCTATATGTGTTTAAGTTTTTATTCATTTGTATGGTTGGATAAATATCTTAGACTTGATGTTAGTATAAATGATATATAT 1300
AnTat1.1_ND4   1201 ATTTTTATCATCAATATACATCTATATGTGTTTAAGTTTTTATTCATTTGTATGGTTGGATAAATATCTTAGACTTGATGTTAGTATAAATGATATATAT 1300
BF_ND4         1201 ATTTTTATCATCAATATACATCTATATGTGTTTAAGTTTTTATTCATTTGTATGGTTGGATAAATATCTTAGACTTGATGTTAGTATAAATGATATATAT 1300
SM_ND4         1201 ATTTTTATCATCAATATACATCTATATGTGTTTAAGTTTTTATTCATTTGTATGGTTGGATAAATATCTTAGACTTGATGTTAGTATAAATGATATATAT 1300
PF_ND4         1201 ATTTTTATCATCAATATACATCTATATGTGTTTAAGTTTTTATTCATTTGTATGGTTGGATAAATATCTTAGACTTGATGTTAGTATAAATGATATATAT 1300
ref_ND4        1201 ATTTTTATCATCAATATACATCTATATGTGTTTAAGTTTTTATTCATTTGTATGGTTGGATAAATATCTTAGACTTGATGTTAGTATAAATGATATATAT 1300

29-13_ND4      1301 GTATTTATGTCAATATCAATATCAACTATAGTATTTTATTATTTTATATATTTATTAATATAA                                      1363
AnTat1.1_ND4   1301 GTATTTATGTCAATATCAATATCAACTATAGTATTTTATTATTTTATATATTTATTAATATAA                                      1363
BF_ND4         1301 GTATTTATGTCAATATCAATATCAACTATAGTATTTTATTATTTTATATATTTATTAATATAA                                      1363
SM_ND4         1301 GTATTTATGTCAATATCAATATCAACTATAGTATTTTATTATTTTATATATTTATTAATATAA                                      1363
PF_ND4         1301 GTATTTATGTCAATATCAATATCAACTATAGTATTTTATTATTTTATATATTTATTAATATAA                                      1363
ref_ND4        1301 GTATTTATGTCAATATCAATATCAACTATAGTATTTTATTATTTTATATATTTATTAATATAA                                      1363
```

ND5

```
29-13_ND5         1 AGAAACATCGTTTAGAAGAGATTTTAGAATAAGATATGTTTTTAATATTTTTTTTATTTTTTTATAATGTTTGGGTTTATATCAGGTTCATTTATGTTTGG 100
AnTat1.1_ND5      1 AGAAACATCGTTTAGAAGAGATTTTAGAATAAGATATGTTTTTAATATTTTTTTTATTTTTTTATAATGTTTGGGTTTATATCAGGTTCATTTATGTTTGG 100
BF_ND5            1 AGAAACATCGTTTAGAAGAGATTTTAGAATAAGATATGTTTTTAATATTTTTTTTATTTTTTTATAATGTTTGGGTTTATATCAGGTTCATTTATGTTTGG 100
SM_ND5            1 AGAAACATCGTTTAGAAGAGATTTTAGAATAAGATATGTTTTTAATATTTTTTTTATTTTTTTATAATGTTTGGGTTTATATCAGGTTCATTTATGTTTGG 100
PF_ND5            1 AGAAACATCGTTTAGAAGAGATTTTAGAATAAGATATGTTTTTAATATTTTTTTTATTTTTTTATAATGTTTGGGTTTATATCAGGTTCATTTATGTTTGG 100
ref_ND5           1 AGAAACATCGTTTAGAAGAGATTTTAGAATAAGATATGTTTTTAATATTTTTTTTATTTTTTTATAATGTTTGGGTTTATATCAGGTTCATTTATGTTTGG 100

29-13_ND5       101 TAGGAATTTTCTAAGTTTTTGATTATCTTTAGTAATGATAATATTTATTGTATTGTGTATGATATTTAGTTTTTTAATGGTATCAGTATGTTTATACGGT 200
AnTat1.1_ND5    101 TAGGAATTTTCTAAGTTTTTGATTATCTTTAGTAATGATAATATTTATTGTATTGTGTATGATATTTAGTTTTTTAATGGTATCAGTATGTTTATACGGT 200
BF_ND5          101 TAGGAATTTTCTAAGTTTTTGATTATCTTTAGTAATGATAATATTTATTGTATTGTGTATGATATTTAGTTTTTTAATGGTATCAGTATGTTTATACGGT 200
SM_ND5          101 TAGGAATTTTCTAAGTTTTTGATTATCTTTAGTAATGATAATATTTATTGTATTGTGTATGATATTTAGTTTTTTAATGGTATCAGTATGTTTATACGGT 200
PF_ND5          101 TAGGAATTTTCTAAGTTTTTGATTATCTTTAGTAATGATAATATTTATTGTATTGTGTATGATATTTAGTTTTTTAATGGTATCAGTATGTTTATACGGT 200
ref_ND5         101 TAGGAATTTTCTAAGTTTTTGATTATCTTTAGTAATGATAATATTTATTGTATTGTGTATGATATTTAGTTTTTTAATGGTATCAGTATGTTTATACGGT 200

29-13_ND5       201 TATTATTATTACGATTTTTGTTTAATACTAATGTTAGATTTTTGTTTTATATGATTAACATACGTATGTTCAGGTTTTTATATGTTTATAATGTTATTGA 300
AnTat1.1_ND5    201 TATTATTATTACGATTTTTGTTTAATACTAATGTTAGATTTTTGTTTTATATGATTAACATACGTATGTTCAGGTTTTTATATGTTTATAATGTTATTGA 300
BF_ND5          201 TATTATTATTACGATTTTTGTTTAATACTAATGTTAGATTTTTGTTTTATATGATTAACATACGTATGTTCAGGTTTTTATATGTTTATAATGTTATTGA 300
SM_ND5          201 TATTATTATTACGATTTTTGTTTAATACTAATGTTAGATTTTTGTTTTATATGATTAACATACGTATGTTCAGGTTTTTATATGTTTATAATGTTATTGA 300
PF_ND5          201 TATTATTATTACGATTTTTGTTTAATACTAATGTTAGATTTTTGTTTTATATGATTAACATACGTATGTTCAGGTTTTTATATGTTTATAATGTTATTGA 300
ref_ND5         201 TATTATTATTACGATTTTTGTTTAATACTAATGTTAGATTTTTGTTTTATATGATTAACATACGTATGTTCAGGTTTTTATATGTTTATAATGTTATTGA 300

                                                   *
29-13_ND5       301 TAAATATGGTATTTTGTTTTATAGTATTTTACGCATTTTATTATATGTATTTTGATATGTTGTTAGGGCGTTTTTTGATTATATTTTGAATATTTGTTGT 400
AnTat1.1_ND5    301 TAAATATGGTATTTTGTTTTATAGTATTTTATGCATTTTATTATATGTATTTTGATATGTTGTTAGGGCGTTTTTTGATTATATTTTGAATATTTGTTGT 400
BF_ND5          301 TAAATATGGTATTTTGTTTTATAGTATTTTACGCATTTTATTATATGTATTTTGATATGTTGTTAGGGCGTTTTTTGATTATATTTTGAATATTTGTTGT 400
SM_ND5          301 TAAATATGGTATTTTGTTTTATAGTATTTTACGCATTTTATTATATGTATTTTGATATGTTGTTAGGGCGTTTTTTGATTATATTTTGAATATTTGTTGT 400
PF_ND5          301 TAAATATGGTATTTTGTTTTATAGTATTTTACGCATTTTATTATATGTATTTTGATATGTTGTTAGGGCGTTTTTTGATTATATTTTGAATATTTGTTGT 400
ref_ND5         301 TAAATATGGTATTTTGTTTTATAGTATTTTACGCATTTTATTATATGTATTTTGATATGTTGTTAGGGCGTTTTTTGATTATATTTTGAATATTTGTTGT 400

                                               *                 *                     *
29-13_ND5       401 GTGCATGAATTTATTCATCCTATCATATGATTTTTTAACAGCTTACTGCGGATGAGAATTATTAGGGTTATTCTCATTTTTTTTAATTTCATATTTCTGA 500
AnTat1.1_ND5    401 GTGCATGAATTTATTCATCCTATCATACGATTTTTTAACAGCTTATTGCGGATGAGAATTATTAGGGCTATTCTCATTTTTTTTAATTTCATATTTCTGA 500
BF_ND5          401 GTGCATGAATTTATTCATCCTATCATATGATTTTTTAACAGCTTACTGCGGATGAGAATTATTAGGGTTATTCTCATTTTTTTTAATTTCATATTTCTGA 500
SM_ND5          401 GTGCATGAATTTATTCATCCTATCATATGATTTTTTAACAGCTTACTGCGGATGAGAATTATTAGGGTTATTCTCATTTTTTTTAATTTCATATTTCTGA 500
PF_ND5          401 GTGCATGAATTTATTCATCCTATCATATGATTTTTTAACAGCTTACTGCGGATGAGAATTATTAGGGTTATTCTCATTTTTTTTAATTTCATATTTCTGA 500
ref_ND5         401 GTGCATGAATTTATTCATCCTATCATATGATTTTTTAACAGCTTACTGCGGATGAGAATTATTAGGGTTATTCTCATTTTTTTTAATTTCATATTTCTGA 500

29-13_ND5       501 TACCGTTTTTTTGCATTAAAATTTGGTTTTAAAGCTTTTTTTATAGGTAAAATAGGAGATGTGTTATTAATATTCGCTTTTTCTATAATATTTTTTATCAA 600
AnTat1.1_ND5    501 TACCGTTTTTTTGCATTAAAATTTGGTTTTAAAGCTTTTTTTATAGGTAAAATAGGAGATGTGTTATTAATATTCGCTTTTTCTATAATATTTTTTATCAA 600
BF_ND5          501 TACCGTTTTTTTGCATTAAAATTTGGTTTTAAAGCTTTTTTTATAGGTAAAATAGGAGATGTGTTATTAATATTCGCTTTTTCTATAATATTTTTTATCAA 600
SM_ND5          501 TACCGTTTTTTTGCATTAAAATTTGGTTTTAAAGCTTTTTTTATAGGTAAAATAGGAGATGTGTTATTAATATTCGCTTTTTCTATAATATTTTTTATCAA 600
PF_ND5          501 TACCGTTTTTTTGCATTAAAATTTGGTTTTAAAGCTTTTTTTATAGGTAAAATAGGAGATGTGTTATTAATATTCGCTTTTTCTATAATATTTTTTATCAA 600
ref_ND5         501 TACCGTTTTTTTGCATTAAAATTTGGTTTTAAAGCTTTTTTTATAGGTAAAATAGGAGATGTGTTATTAATATTCGCTTTTTCTATAATATTTTTTATCAA 600

29-13_ND5       601 ATGGTTTTTGTATGACAACTTTTTATTTTTTAAATTTTTTTTGTATGGATTATTATTATATAGAATTTTCTATATGTTTGTTAGTAGGATGTGCGTTCAC 700
AnTat1.1_ND5    601 ATGGTTTTTGTATGACAACTTTTTATTTTTTAAATTTTTTTTGTATGGATTATTATTATATAGAATTTTCTATATGTTTGTTAGTAGGATGTGCGTTCAC 700
BF_ND5          601 ATGGTTTTTGTATGACAACTTTTTATTTTTTAAATTTTTTTTGTATGGATTATTATTATATAGAATTTTCTATATGTTTGTTAGTAGGATGTGCGTTCAC 700
SM_ND5          601 ATGGTTTTTGTATGACAACTTTTTATTTTTTAAATTTTTTTTGTATGGATTATTATTATATAGAATTTTCTATATGTTTGTTAGTAGGATGTGCGTTCAC 700
PF_ND5          601 ATGGTTTTTGTATGACAACTTTTTATTTTTTAAATTTTTTTTGTATGGATTATTATTATATAGAATTTTCTATATGTTTGTTAGTAGGATGTGCGTTCAC 700
ref_ND5         601 ATGGTTTTTGTATGACAACTTTTTATTTTTTAAATTTTTTTTGTATGGATTATTATTATATAGAATTTTCTATATGTTTGTTAGTAGGATGTGCGTTCAC 700

                                   *                                                      *       *
29-13_ND5       701 AAAAAGTACACAATTCGGCTTACATATATGATTACCAGATGCTATGGAAGGACCTATCCCAGTATCAGCATTAATACACGCAGCTACATTAGTTGTTTGT 800
AnTat1.1_ND5    701 AAAAAGTACACAATTTGGCTTACATATATGATTACCAGATGCTATGGAAGGACCTATCCCAGTATCAGCACTAATACATGCAGCTACATTAGTTGTTTGT 800
BF_ND5          701 AAAAAGTACACAATTCGGCTTACATATATGATTACCAGATGCTATGGAAGGACCTATCCCAGTATCAGCATTAATACACGCAGCTACATTAGTTGTTTGT 800
SM_ND5          701 AAAAAGTACACAATTCGGCTTACATATATGATTACCAGATGCTATGGAAGGACCTATCCCAGTATCAGCATTAATACACGCAGCTACATTAGTTGTTTGT 800
PF_ND5          701 AAAAAGTACACAATTCGGCTTACATATATGATTACCAGATGCTATGGAAGGACCTATCCCAGTATCAGCATTAATACACGCAGCTACATTAGTTGTTTGT 800
ref_ND5         701 AAAAAGTACACAATTCGGCTTACATATATGATTACCAGATGCTATGGAAGGACCTATCCCAGTATCAGCATTAATACACGCAGCTACATTAGTTGTTTGT 800

29-13_ND5       801 GGAATAATATTATTAAGTTTTGTTTATTGATGTTTTGATTTTTGATTTAGTTATTTTTTATAATTTGATAGGATGGTCTACATTAATTTTAATATTAATGA 900
AnTat1.1_ND5    801 GGAATAATATTATTAAGTTTTGTTTATTGATGTTTTGATTTTTGATTTAGTTATTTTTTATAATTTGATAGGATGGTCTACATTAATTTTAATATTAATGA 900
BF_ND5          801 GGAATAATATTATTAAGTTTTGTTTATTGATGTTTTGATTTTTGATTTAGTTATTTTTTATAATTTGATAGGATGGTCTACATTAATTTTAATATTAATGA 900
SM_ND5          801 GGAATAATATTATTAAGTTTTGTTTATTGATGTTTTGATTTTTGATTTAGTTATTTTTTATAATTTGATAGGATGGTCTACATTAATTTTAATATTAATGA 900
PF_ND5          801 GGAATAATATTATTAAGTTTTGTTTATTGATGTTTTGATTTTTGATTTAGTTATTTTTTATAATTTGATAGGATGGTCTACATTAATTTTAATATTAATGA 900
ref_ND5         801 GGAATAATATTATTAAGTTTTGTTTATTGATGTTTTGATTTTTGATTTAGTTATTTTTTATAATTTGATAGGATGGTCTACATTAATTTTAATATTAATGA 900

29-13_ND5       901 CATTGTGTGTGTTTTATAATTTTGACGTAAAACGATACGTAGCGTTCAGTACAATATGTCAAATTAGTTTTTCTATGTTTTGTTGTCTGTGTATAGATAT 1000
AnTat1.1_ND5    901 CATTGTGTGTGTTTTATAATTTTGACGTAAAACGATACGTAGCGTTCAGTACAATATGTCAAATTAGTTTTTCTATGTTTTGTTGTCTGTGTATAGATAT 1000
BF_ND5          901 CATTGTGTGTGTTTTATAATTTTGACGTAAAACGATACGTAGCGTTCAGTACAATATGTCAAATTAGTTTTTCTATGTTTTGTTGTCTGTGTATAGATAT 1000
SM_ND5          901 CATTGTGTGTGTTTTATAATTTTGACGTAAAACGATACGTAGCGTTCAGTACAATATGTCAAATTAGTTTTTCTATGTTTTGTTGTCTGTGTATAGATAT 1000
PF_ND5          901 CATTGTGTGTGTTTTATAATTTTGACGTAAAACGATACGTAGCGTTCAGTACAATATGTCAAATTAGTTTTTCTATGTTTTGTTGTCTGTGTATAGATAT 1000
ref_ND5         901 CATTGTGTGTGTTTTATAATTTTGACGTAAAACGATACGTAGCGTTCAGTACAATATGTCAAATTAGTTTTTCTATGTTTTGTTGTCTGTGTATAGATAT 1000

29-13_ND5      1001 ATATATAGGTAGTTTATTTTTTTTGTTACCATATGTTCTACAAAGCAACATTATTTATAGTATTAGGTATATGAATACATATATTTTTTGGGTTACAGGAT 1100
AnTat1.1_ND5   1001 ATATATAGGTAGTTTATTTTTTTTGTTACCATATGTTCTACAAAGCAACATTATTTATAGTATTAGGTATATGAATACATATATTTTTTGGGTTACAGGAT 1100
BF_ND5         1001 ATATATAGGTAGTTTATTTTTTTTGTTACCATATGTTCTACAAAGCAACATTATTTATAGTATTAGGTATATGAATACATATATTTTTTGGGTTACAGGAT 1100
SM_ND5         1001 ATATATAGGTAGTTTATTTTTTTTGTTACCATATGTTCTACAAAGCAACATTATTTATAGTATTAGGTATATGAATACATATATTTTTTGGGTTACAGGAT 1100
PF_ND5         1001 ATATATAGGTAGTTTATTTTTTTTGTTACCATATGTTCTACAAAGCAACATTATTTATAGTATTAGGTATATGAATACATATATTTTTTGGGTTACAGGAT 1100
ref_ND5        1001 ATATATAGGTAGTTTATTTTTTTTGTTACCATATGTTCTACAAAGCAACATTATTTATAGTATTAGGTATATGAATACATATATTTTTTGGGTTACAGGAT 1100

                                                                                          *
29-13_ND5      1101 TTAAGATGTTATTTTTTTTATGTATTTTTGTGGTTGTGTGTTAGCGCGTTTGTTATTAATATTCGCAATATTAAACTCATGTTCAATTTGATTTTTATGTG 1200
AnTat1.1_ND5   1101 TTAAGATGTTATTTTTTTTATGTATTTTTGTGGTTGTGTGTTAGCGCGTTTGTTATTAATATTCGCAATACTAAACTCATGTTCAATTTGATTTTTATGTG 1200
BF_ND5         1101 TTAAGATGTTATTTTTTTTATGTATTTTTGTGGTTGTGTGTTAGCGCGTTTGTTATTAATATTCGCAATATTAAACTCATGTTCAATTTGATTTTTATGTG 1200
SM_ND5         1101 TTAAGATGTTATTTTTTTTATGTATTTTTGTGGTTGTGTGTTAGCGCGTTTGTTATTAATATTCGCAATATTAAACTCATGTTCAATTTGATTTTTATGTG 1200
PF_ND5         1101 TTAAGATGTTATTTTTTTTATGTATTTTTGTGGTTGTGTGTTAGCGCGTTTGTTATTAATATTCGCAATATTAAACTCATGTTCAATTTGATTTTTATGTG 1200
ref_ND5        1101 TTAAGATGTTATTTTTTTTATGTATTTTTGTGGTTGTGTGTTAGCGCGTTTGTTATTAATATTCGCAATATTAAACTCATGTTCAATTTGATTTTTATGTG 1200

29-13_ND5      1201 GTTTTTATTGTAAGGATATGTTATTAGCTTTATTGATGTTATTATCATTTTATAATATAATAGAATTTTTGTTTATAAGTATAATTTTTATATTTTTTAC 1300
AnTat1.1_ND5   1201 GTTTTTATTGTAAGGATATGTTATTAGCTTTATTGATGTTATTATCATTTTATAATATAATAGAATTTTTGTTTATAAGTATAATTTTTATATTTTTTAC 1300
BF_ND5         1201 GTTTTTATTGTAAGGATATGTTATTAGCTTTATTGATGTTATTATCATTTTATAATATAATAGAATTTTTGTTTATAAGTATAATTTTTATATTTTTTAC 1300
SM_ND5         1201 GTTTTTATTGTAAGGATATGTTATTAGCTTTATTGATGTTATTATCATTTTATAATATAATAGAATTTTTGTTTATAAGTATAATTTTTATATTTTTTAC 1300
PF_ND5         1201 GTTTTTATTGTAAGGATATGTTATTAGCTTTATTGATGTTATTATCATTTTATAATATAATAGAATTTTTGTTTATAAGTATAATTTTTATATTTTTTAC 1300
ref_ND5        1201 GTTTTTATTGTAAGGATATGTTATTAGCTTTATTGATGTTATTATCATTTTATAATATAATAGAATTTTTGTTTATAAGTATAATTTTTATATTTTTTAC 1300

29-13_ND5      1301 AATGATTTATAATTATTTTTTGTTATTTTTTTTTGATGTTTGTGTTCAAATGTTTTTGTTTGGTTGATTGTTTATTTTTTATTATTTGATTATGAATGTTGT 1400
AnTat1.1_ND5   1301 AATGATTTATAATTATTTTTTGTTATTTTTTTTTGATGTTTGTGTTCAAATGTTTTTGTTTGGTTGATTGTTTATTTTTTATTATTTGATTATGAATGTTGT 1400
BF_ND5         1301 AATGATTTATAATTATTTTTTGTTATTTTTTTTTGATGTTTGTGTTCAAATGTTTTTGTTTGGTTGATTGTTTATTTTTTATTATTTGATTATGAATGTTGT 1400
SM_ND5         1301 AATGATTTATAATTATTTTTTGTTATTTTTTTTTGATGTTTGTGTTCAAATGTTTTTGTTTGGTTGATTGTTTATTTTTTATTATTTGATTATGAATGTTGT 1400
PF_ND5         1301 AATGATTTATAATTATTTTTTGTTATTTTTTTTTGATGTTTGTGTTCAAATGTTTTTGTTTGGTTGATTGTTTATTTTTTATTATTTGATTATGAATGTTGT 1400
ref_ND5        1301 AATGATTTATAATTATTTTTTGTTATTTTTTTTTGATGTTTGTGTTCAAATGTTTTTGTTTGGTTGATTGTTTATTTTTTATTATTTGATTATGAATGTTGT 1400

                         *
29-13_ND5      1401 TTAGTATATTGTTTGATAAGTTTGTATATGTGTATTTTAAGTATATTTTTTTATAATAGATTTTGTATGTATATTTGTATTTTCAAGTTATTGTGTATTTT 1500
AnTat1.1_ND5   1401 TTAGTGTATTGTTTGATAAGTTTGTATATGTGTATTTTAAGTATATTTTTTTATAATAGATTTTGTATGTATATTTGTATTTTCAAGTTATTGTGTATTTT 1500
BF_ND5         1401 TTAGTATATTGTTTGATAAGTTTGTATATGTGTATTTTAAGTATATTTTTTTATAATAGATTTTGTATGTATATTTGTATTTTCAAGTTATTGTGTATTTT 1500
SM_ND5         1401 TTAGTATATTGTTTGATAAGTTTGTATATGTGTATTTTAAGTATATTTTTTTATAATAGATTTTGTATGTATATTTGTATTTTCAAGTTATTGTGTATTTT 1500
PF_ND5         1401 TTAGTATATTGTTTGATAAGTTTGTATATGTGTATTTTAAGTATATTTTTTTATAATAGATTTTGTATGTATATTTGTATTTTCAAGTTATTGTGTATTTT 1500
ref_ND5        1401 TTAGTATATTGTTTGATAAGTTTGTATATGTGTATTTTAAGTATATTTTTTTATAATAGATTTTGTATGTATATTTGTATTTTCAAGTTATTGTGTATTTT 1500

29-13_ND5      1501 GATCATTTTTTTTAAATTTTTATAATTTTTTTGATATAGCAATTTTTGTGGTTTTTTTAATATTATCAGTAGGATTTTTATATTATGGTTGTTTATTTTTT 1600
AnTat1.1_ND5   1501 GATCATTTTTTTTAAATTTTTATAATTTTTTTGATATAGCAATTTTTGTGGTTTTTTTAATATTATCAGTAGGATTTTTATATTATGGTTGTTTATTTTTT 1600
BF_ND5         1501 GATCATTTTTTTTAAATTTTTATAATTTTTTTGATATAGCAATTTTTGTGGTTTTTTTAATATTATCAGTAGGATTTTTATATTATGGTTGTTTATTTTTT 1600
SM_ND5         1501 GATCATTTTTTTTAAATTTTTATAATTTTTTTGATATAGCAATTTTTGTGGTTTTTTTAATATTATCAGTAGGATTTTTATATTATGGTTGTTTATTTTTT 1600
PF_ND5         1501 GATCATTTTTTTTAAATTTTTATAATTTTTTTGATATAGCAATTTTTGTGGTTTTTTTAATATTATCAGTAGGATTTTTATATTATGGTTGTTTATTTTTT 1600
ref_ND5        1501 GATCATTTTTTTTAAATTTTTATAATTTTTTTGATATAGCAATTTTTGTGGTTTTTTTAATATTATCAGTAGGATTTTTATATTATGGTTGTTTATTTTTT 1600

                                         *
29-13_ND5      1601 TTATTTTTTCAATATAGATTGCATAATGTTGTTTTGGAGAATTTTTTTTGTAATAATAATTTTAGTAGTATTTATGATATTTTGTTGTTGATATTTTGTT 1700
AnTat1.1_ND5   1601 TTATTTTTTCAATATAGATTGTATAATGTTGTTTTGGAGAATTTTTTTTGTAATAATAATTTTAGTAGTATTTATGATATTTTGTTGTTGATATTTTGTT 1700
BF_ND5         1601 TTATTTTTTCAATATAGATTGCATAATGTTGTTTTGGAGAATTTTTTTTGTAATAATAATTTTAGTAGTATTTATGATATTTTGTTGTTGATATTTTGTT 1700
SM_ND5         1601 TTATTTTTTCAATATAGATTGCATAATGTTGTTTTGGAGAATTTTTTTTGTAATAATAATTTTAGTAGTATTTATGATATTTTGTTGTTGATATTTTGTT 1700
PF_ND5         1601 TTATTTTTTCAATATAGATTGCATAATGTTGTTTTGGAGAATTTTTTTTGTAATAATAATTTTAGTAGTATTTATGATATTTTGTTGTTGATATTTTGTT 1700
ref_ND5        1601 TTATTTTTTCAATATAGATTGCATAATGTTGTTTTGGAGAATTTTTTTTGTAATAATAATTTTAGTAGTATTTATGATATTTTGTTGTTGATATTTTGTT 1700

                                                                                                                *
29-13_ND5      1701 TGTATGATCATATTTATGTTATTATTTGTATGAAATTTTGTTATATATTTTAGATATAATTTGAAATATTGTTTATTTTTTTTGTATTTTGTGAATATTGT 1800
AnTat1.1_ND5   1701 TGTATGATCATATTTATGTTATTATTTGTATGAAATTTTGTTATATATTTTAGATATAATTTGAAATATTGTTTATTTTTTTTGTATTTTGTGGATATTGT 1800
BF_ND5         1701 TGTATGATCATATTTATGTTATTATTTGTATGAAATTTTGTTATATATTTTAGATATAATTTGAAATATTGTTTATTTTTTTTGTATTTTGTGAATATTGT 1800
SM_ND5         1701 TGTATGATCATATTTATGTTATTATTTGTATGAAATTTTGTTATATATTTTAGATATAATTTGAAATATTGTTTATTTTTTTTGTATTTTGTGAATATTGT 1800
PF_ND5         1701 TGTATGATCATATTTATGTTATTATTTGTATGAAATTTTGTTATATATTTTAGATATAATTTGAAATATTGTTTATTTTTTTTGTATTTTGTGAATATTGT 1800
ref_ND5        1701 TGTATGATCATATTTATGTTATTATTTGTATGAAATTTTGTTATATATTTTAGATATAATTTGAAATATTGTTTATTTTTTTTGTATTTTGTGAATATTGT 1800

29-13_ND5      1801 ATGTATAA 1808
AnTat1.1_ND5   1801 ATGTATAA 1808
BF_ND5         1801 ATGTATAA 1808
SM_ND5         1801 ATGTATAA 1808
PF_ND5         1801 ATGTATAA 1808
ref_ND5        1801 ATGTATAA 1808
```

ND7

```
                     * *
29-13_ND7      1 TGATAC-AAAAAAACATGACTACATGATAAGTACAAGAGGAGACAGACGACAGTGTCCACAGCACCCGTTTCAGCACAGTTGGAGGAGAGGGGATAAGAT 99
AnTat1.1_ND7   1 TGATATAAAAAAAACATGACTACATGATAAGTACAAGAGGAGACAGACGACAGTGTCCACAGCACCCGTTTCAGCACAGTTGGAGGAGAGGGGATAAGAT 100
BF_ND7         1 TGATAC-AAAAAAACATGACTACATGATAAGTACAAGAGGAGACAGACGACAGTGTCCACAGCACCCGTTTCAGCACAGTTGGAGGAGAGGGGATAAGAT 99
SM_ND7         1 TGATAC-AAAAAAACATGACTACATGATAAGTACAAGAGGAGACAGACGACAGTGTCCACAGCACCCGTTTCAGCACAGTTGGAGGAGAGGGGATAAGAT 99
PF_ND7         1 TGATAC-AAAAAAACATGACTACATGATAAGTACAAGAGGAGACAGACGACAGTGTCCACAGCACCCGTTTCAGCACAGTTGGAGGAGAGGGGATAAGAT 99
ref_ND7        1 TGATAC-AAAAAAACATGACTACATGATAAGTACAAGAGGAGACAGACGACAGTGTCCACAGCACCCGTTTCAGCACAGTTGGAGGAGAGGGGATAAGAT 99

29-13_ND7    100 TTATTGATGAAATTTGTGATTTGCATCGTGGTACAGAAAAGTTATGTGAATATAAAAGTGTAGAACAATGTCTTCCGATTTCGACAGGTTAGAAGATGGG 199
AnTat1.1_ND7 101 TTATTGATGAAATTTGTGATTTGCATCGTGGTACAGAAAAGTTATGTGAATATAAAAGTGTAGAACAATGTCTTCCGATTTCGACAGGTTAGAAGATGGG 200
BF_ND7       100 TTATTGATGAAATTTGTGATTTGCATCGTGGTACAGAAAAGTTATGTGAATATAAAAGTGTAGAACAATGTCTTCCGATTTCGACAGGTTAGAAGATGGG 199
SM_ND7       100 TTATTGATGAAATTTGTGATTTGCATCGTGGTACAGAAAAGTTATGTGAATATAAAAGTGTAGAACAATGTCTTCCGATTTCGACAGGTTAGAAGATGGG 199
PF_ND7       100 TTATTGATGAAATTTGTGATTTGCATCGTGGTACAGAAAAGTTATGTGAATATAAAAGTGTAGAACAATGTCTTCCGATTTCGACAGGTTAGAAGATGGG 199
ref_ND7      100 TTATTGATGAAATTTGTGATTTGCATCGTGGTACAGAAAAGTTATGTGAATATAAAAGTGTAGAACAATGTCTTCCGATTTCGACAGGTTAGAAGATGGG 199

                                                                 *                     *
29-13_ND7    200 GAAGAGCAGGCATTTTGGAGAAGGCGAGGGCGACGGGCAAGCGAAAGATTTTGAAACTTTCCGAGAAGGGGGAACAGAGGGGTAAGGGGCTCCGGTTTAG 299
AnTat1.1_ND7 201 GAAGAGCAGGCATTTTGGAGAAGGCGAGGGCGACGGGCAAGCGAAAGA-TTTGAAACTTTCCGAGAAGGAGGAACAGAGGGGTAAGGGGCTCCGGTTTAG 299
BF_ND7       200 GAAGAGCAGGCATTTTGGAGAAGGCGAGGGCGACGGGCAAGCGAAAGATTTTGAAACTTTCCGAGAAGGGGGAACAGAGGGGTAAGGGGCTCCGGTTTAG 299
SM_ND7       200 GAAGAGCAGGCATTTTGGAGAAGGCGAGGGCGACGGGCAAGCGAAAGATTTTGAAACTTTCCGAGAAGGGGGAACAGAGGGGTAAGGGGCTCCGGTTTAG 299
PF_ND7       200 GAAGAGCAGGCATTTTGGAGAAGGCGAGGGCGACGGGCAAGCGAAAGATTTTGAAACTTTCCGAGAAGGGGGAACAGAGGGGTAAGGGGCTCCGGTTTAG 299
ref_ND7      200 GAAGAGCAGGCATTTTGGAGAAGGCGAGGGCGACGGGCAAGCGAAAGATTTTGAAACTTTCCGAGAAGGGGGAACAGAGGGGTAAGGGGCTCCGGTTTAG 299

29-13_ND7    300 ACAGAGGAATTTCGTTGACAAAGAGACAGAAGTTTTGGGGCGAGCAGGCTTTCAGGAATGGATTCTTGATGAGGGGGAGGGGATTTTAAACAGGGAGGAG 399
AnTat1.1_ND7 300 ACAGAGGAATTTCGTTGACAAAGAGACAGAAGTTTTGGGGCGAGCAGGCTTTCAGGAATGGATTCTTGATGAGGGGGAGGGGATTTTAAACAGGGAGGAG 399
BF_ND7       300 ACAGAGGAATTTCGTTGACAAAGAGACAGAAGTTTTGGGGCGAGCAGGCTTTCAGGAATGGATTCTTGATGAGGGGGAGGGGATTTTAAACAGGGAGGAG 399
SM_ND7       300 ACAGAGGAATTTCGTTGACAAAGAGACAGAAGTTTTGGGGCGAGCAGGCTTTCAGGAATGGATTCTTGATGAGGGGGAGGGGATTTTAAACAGGGAGGAG 399
PF_ND7       300 ACAGAGGAATTTCGTTGACAAAGAGACAGAAGTTTTGGGGCGAGCAGGCTTTCAGGAATGGATTCTTGATGAGGGGGAGGGGATTTTAAACAGGGAGGAG 399
ref_ND7      300 ACAGAGGAATTTCGTTGACAAAGAGACAGAAGTTTTGGGGCGAGCAGGCTTTCAGGAATGGATTCTTGATGAGGGGGAGGGGATTTTAAACAGGGAGGAG 399

                                                                                                  *
29-13_ND7    400 AGAGAGGGGAATCGATAGCGGCTTTGGGGCAGAAAGAATTGATTATTTAGAAGGGGGCCGCGAGGAGGGGAGAGTCGAAGGATTTTTGATTTTTGTGAAG 499
AnTat1.1_ND7 400 AGAGAGGGGAATCGATAGCGGCTTTGGGGCAGAAAGAATTGATTATTTAGAAGGGGGCCGCGAGGAGGGGAGAGTCGAAGGATTTTTGA-TTTTGTGAAG 498
BF_ND7       400 AGAGAGGGGAATCGATAGCGGCTTTGGGGCAGAAAGAATTGATTATTTAGAAGGGGGCCGCGAGGAGGGGAGAGTCGAAGGATTTTTGATTTTTGTGAAG 499
SM_ND7       400 AGAGAGGGGAATCGATAGCGGCTTTGGGGCAGAAAGAATTGATTATTTAGAAGGGGGCCGCGAGGAGGGGAGAGTCGAAGGATTTTTGATTTTTGTGAAG 499
PF_ND7       400 AGAGAGGGGAATCGATAGCGGCTTTGGGGCAGAAAGAATTGATTATTTAGAAGGGGGCCGCGAGGAGGGGAGAGTCGAAGGATTTTTGATTTTTGTGAAG 499
ref_ND7      400 AGAGAGGGGAATCGATAGCGGCTTTGGGGCAGAAAGAATTGATTATTTAGAAGGGGGCCGCGAGGAGGGGAGAGTCGAAGGATTTTTGATTTTTGTGAAG 499

                                              *                                                                  *
29-13_ND7    500 GAGAAGGAAGGGAGCAGATTCGAACGGGA-TAGCGAGAGGGAGAAGCAAGGGGGGTTTTTGGGGGTTAAAAGGAAACCAGTTTTAGACCAAAGAAAG-GG 597
AnTat1.1_ND7 499 GAGAAGGAAGGGAGCAGATTCGAACGGGATTAGCGAGAGGGAGAAGCAAGGGGGGTTTTTGGGGGTTAAAAGGAAACCAGTTTTAGACCAAAGAAAGTGG 598
BF_ND7       500 GAGAAGGAAGGGAGCAGATTCGAACGGGA-TAGCGAGAGGGAGAAGCAAGGGGGGTTTTTGGGGGTTAAAAGGAAACCAGTTTTAGACCAAAGAAAG-GG 597
SM_ND7       500 GAGAAGGAAGGGAGCAGATTCGAACGGGA-TAGCGAGAGGGAGAAGCAAGGGGGGTTTTTGGGGGTTAAAAGGAAACCAGTTTTAGACCAAAGAAAG-GG 597
PF_ND7       500 GAGAAGGAAGGGAGCAGATTCGAACGGGA-TAGCGAGAGGGAGAAGCAAGGGGGGTTTTTGGGGGTTAAAAGGAAACCAGTTTTAGACCAAAGAAAG-GG 597
ref_ND7      500 GAGAAGGAAGGGAGCAGATTCGAACGGGA-TAGCGAGAGGGAGAAGCAAGGGGGGTTTTTGGGGGTTAAAAGGAAACCAGTTTTAGACCAAAGAAAG-GG 597

29-13_ND7    598 GGGGGCCGGGAATTCAGCTTTGTGGAACACCCCAAAGGGATTTGAGGAATTTTTGGGGGAGCTCGACGGCGGGCGGAGCATTATTTGAGGAGGGCGGGAG 697
AnTat1.1_ND7 599 GGGGGCCGGGAATTCAGCTTTGTGGAACACCCCAAAGGGATTTGAGGAATTTTTGGGGGAGCTCGACGGCGGGCGGAGCATTATTTGAGGAGGGCGGGAG 698
BF_ND7       598 GGGGGCCGGGAATTCAGCTTTGTGGAACACCCCAAAGGGATTTGAGGAATTTTTGGGGGAGCTCGACGGCGGGCGGAGCATTATTTGAGGAGGGCGGGAG 697
SM_ND7       598 GGGGGCCGGGAATTCAGCTTTGTGGAACACCCCAAAGGGATTTGAGGAATTTTTGGGGGAGCTCGACGGCGGGCGGAGCATTATTTGAGGAGGGCGGGAG 697
PF_ND7       598 GGGGGCCGGGAATTCAGCTTTGTGGAACACCCCAAAGGGATTTGAGGAATTTTTGGGGGAGCTCGACGGCGGGCGGAGCATTATTTGAGGAGGGCGGGAG 697
ref_ND7      598 GGGGGCCGGGAATTCAGCTTTGTGGAACACCCCAAAGGGATTTGAGGAATTTTTGGGGGAGCTCGACGGCGGGCGGAGCATTATTTGAGGAGGGCGGGAG 697

                                                             *
29-13_ND7    698 CAGAAGGCTTTCTGAGGAAAGAGGGGACCGAGATCGATGAAGGTTATTTTTTGGTTATTGAGGATTGTTTAAAATTGAATAAAAA 782
AnTat1.1_ND7 699 CAGAAGGCTTTCTGAGGAAAGAGGGGACCGAGATCGATGAAGGTTA-TTTTTGGTTATTGAGGATTGTTTAAAATTGAATAAAAA 782
BF_ND7       698 CAGAAGGCTTTCTGAGGAAAGAGGGGACCGAGATCGATGAAGGTTATTTTTTGGTTATTGAGGATTGTTTAAAATTGAATAAAAA 782
SM_ND7       698 CAGAAGGCTTTCTGAGGAAAGAGGGGACCGAGATCGATGAAGGTTATTTTTTGGTTATTGAGGATTGTTTAAAATTGAATAAAAA 782
PF_ND7       698 CAGAAGGCTTTCTGAGGAAAGAGGGGACCGAGATCGATGAAGGTTATTTTTTGGTTATTGAGGATTGTTTAAAATTGAATAAAAA 782
ref_ND7      698 CAGAAGGCTTTCTGAGGAAAGAGGGGACCGAGATCGATGAAGGTTATTTTTTGGTTATTGAGGATTGTTTAAAATTGAATAAAAA 782
```

ND8

```
29-13_ND8      1 TTAGAATCAATTTAATAATTTTAAGTTTTGGTTGATTAAAAGAGGAGTTTTTGGAAGGTGGGGATTTTCATTTTGATTTCCCAGAGAACCAGAGAGGCGG 100
AnTat1.1_ND8   1 TTAGAATCAATTTAATAATTTTAAGTTTTGGTTGATTAAAAGAGGAGTTTTTGGAAGGTGGGGATTTTCATTTTGATTTCCCAGAGAACCAGAGAGGCGG 100
BF_ND8         1 TTAGAATCAATTTAATAATTTTAAGTTTTGGTTGATTAAAAGAGGAGTTTTTGGAAGGTGGGGATTTTCATTTTGATTTCCCAGAGAACCAGAGAGGCGG 100
SM_ND8         1 TTAGAATCAATTTAATAATTTTAAGTTTTGGTTGATTAAAAGAGGAGTTTTTGGAAGGTGGGGATTTTCATTTTGATTTCCCAGAGAACCAGAGAGGCGG 100
PF_ND8         1 TTAGAATCAATTTAATAATTTTAAGTTTTGGTTGATTAAAAGAGGAGTTTTTGGAAGGTGGGGATTTTCATTTTGATTTCCCAGAGAACCAGAGAGGCGG 100
ref_ND8        1 TTAGAATCAATTTAATAATTTTAAGTTTTGGTTGATTAAAAGAGGAGTTTTTGGAAGGTGGGGATTTTCATTTTGATTTCCCAGAGAACCAGAGAGGCGG 100

29-13_ND8    101 GAACCAGCGTTTTATTTTTGGGGGAGAGCGGAGCGCGAGGAAAGCCCATTTTGAGCAGGAGTTTTTCGGGGGGGAGGGGGCATTTCTGGCGGAGAACAGA 200
AnTat1.1_ND8 101 GAACCAGCGTTTTATTTTTGGGGGAGAGCGGAGCGCGAGGAAAGCCCATTTTGAGCAGGAGTTTTTCGGGGGGGAGGGGGCATTTCTGGCGGAGAACAGA 200
BF_ND8       101 GAACCAGCGTTTTATTTTTGGGGGAGAGCGGAGCGCGAGGAAAGCCCATTTTGAGCAGGAGTTTTTCGGGGGGGAGGGGGCATTTCTGGCGGAGAACAGA 200
SM_ND8       101 GAACCAGCGTTTTATTTTTGGGGGAGAGCGGAGCGCGAGGAAAGCCCATTTTGAGCAGGAGTTTTTCGGGGGGGAGGGGGCATTTCTGGCGGAGAACAGA 200
PF_ND8       101 GAACCAGCGTTTTATTTTTGGGGGAGAGCGGAGCGCGAGGAAAGCCCATTTTGAGCAGGAGTTTTTCGGGGGGGAGGGGGCATTTCTGGCGGAGAACAGA 200
ref_ND8      101 GAACCAGCGTTTTATTTTTGGGGGAGAGCGGAGCGCGAGGAAAGCCCATTTTGAGCAGGAGTTTTTCGGGGGGGAGGGGGCATTTCTGGCGGAGAACAGA 200

                                                                                            *
29-13_ND8    201 GATTCTTGTTTCGGAAGGGGAGCAGGCCCGACAGATTTTTGCCAACGCATTCAGGAGGGGAGCCTTATTTGAAGTGCGCTTTCTTTCAAGAGGGGGAGAG 300
AnTat1.1_ND8 201 GATTCTTGTTTCGGAAGGGGAGCAGGCCCGACAGATTTTTGCCAACGCATTCAGGAGGGGAGCCTTATTTGAAGTGCGCTTTCTTTCAAGAGGGGGAGAG 300
BF_ND8       201 GATTCTTGTTTCGGAAGGGGAGCAGGCCCGACAGATTTTTGCCAACGCATTCAGGAGGGGAGCCTTATTTGAAGTGCGCTTTCTTTCAAGAGGGGGAGAG 300
SM_ND8       201 GATTCTTGTTTCGGAAGGGGAGCAGGCCCGACAGATTTTTGCCAACGCATTCAGGAGGGGAGCCTTATTTGAAGTGCGCTTTCTTTCAAGAGGGGGAGAG 300
PF_ND8       201 GATTCTTGTTTCGGAAGGGGAGCAGGCCCGACAGATTTTTGCCAACGCATTCAGGAGGGGAGCCTTATTTGAAGAGCGCTTTCTTTCAAGAGGGGGAGAG 300
ref_ND8      201 GATTCTTGTTTCGGAAGGGGAGCAGGCCCGACAGATTTTTGCCAACGCATTCAGGAGGGGAGCCTTATTTGAAGTGCGCTTTCTTTCAAGAGGGGGAGAG 300

                                                      *
29-13_ND8    301 AAGGGGAGAAGGGGAAGTGAGAAATTTAGAATTACACGGTGAAATTAAATTTTGACTAAAT 361
AnTat1.1_ND8 301 AAGGGGAGAAGGGGAAGTGAGAAATTTAGAATTACATGGTGAAATTAAATTTTGACTAAAT 361
BF_ND8       301 AAGGGGAGAAGGGGAAGTGAGAAATTTAGAATTACACGGTGAAATTAAATTTTGACTAAAT 361
SM_ND8       301 AAGGGGAGAAGGGGAAGTGAGAAATTTAGAATTACACGGTGAAATTAAATTTTGACTAAAT 361
PF_ND8       301 AAGGGGAGAAGGGGAAGTGAGAAATTTAGAATTACACGGTGAAATTAAATTTTGACTAAAT 361
ref_ND8      301 AAGGGGAGAAGGGGAAGTGAGAAATTTAGAATTACACGGTGAAATTAAATTTTGACTAAAT 361
```

ND9

```
                   *              *****               *                                                     *
29-13_ND9        1 TTAATATCAACTTAA-----TTTTTTTTATAAACAAAATGGAATTTTAGACGAGGAATTAATTTTGGGGAGAGGGTTGGGGAGAGGAGGAGGATTGAGAAA 96
AnTat1.1_ND9     1 -TAATATCAACTTAATTTTTTTTTTTTTATAAACAGAATGGAATTTTAGACGAGGAATTAATTTTGGGGAGAGGGTTGGGGAGAGGAGGGGGATTGAGAAA 100
BF_ND9           1 TTAATATCAACTTAA-----TTTTTTTTATAAACAAAATGGAATTTTAGACGAGGAATTAATTTTGGGGAGAGGGTTGGGGAGAGGAGGAGGATTGAGAAA 96
SM_ND9           1 TTAATATCAACTTAA-----TTTTTTTTATAAACAAAATGGAATTTTAGACGAGGAATTAATTTTGGGGAGAGGGTTGGGGAGAGGAGGAGGATTGAGAAA 96
PF_ND9           1 TTAATATCAACTTAA-----TTTTTTTTATAAACAAAATGGAATTTTAGACGAGGAATTAATTTTGGGGAGAGGGTTGGGGAGAGGAGGAGGATTGAGAAA 96
ref_ND9          1 TTAATATCAACTTAA-----TTTTTTTTATAAACAAAATGGAATTTTAGACGAGGAATTAATTTTGGGGAGAGGGTTGGGGAGAGGAGGAGGATTGAGAAA 96

29-13_ND9       97 GGAGAAGTTGGATTGAGAGAGGGGGCGAGGGTAAAGGGGGCGGGGGGTTTGGTAGGAGGATGATTGGGTAGCGGGGGAACGCTGAAGTGATTTGGGTAGGG 197
AnTat1.1_ND9   101 GGAGAAGTTGGATTGAGAGAGGGGGCGAGGGTAAAGGGGGCGGGGGGTTTGGTAGGAGGATGATTGGGTAGCGGGGGAACGCTGAAGTGATTTGGGTAGGG 201
BF_ND9          97 GGAGAAGTTGGATTGAGAGAGGGGGCGAGGGTAAAGGGGGCGGGGGGTTTGGTAGGAGGATGATTGGGTAGCGGGGGAACGCTGAAGTGATTTGGGTAGGG 197
SM_ND9          97 GGAGAAGTTGGATTGAGAGAGGGGGCGAGGGTAAAGGGGGCGGGGGGTTTGGTAGGAGGATGATTGGGTAGCGGGGGAACGCTGAAGTGATTTGGGTAGGG 197
PF_ND9          97 GGAGAAGTTGGATTGAGAGAGGGGGCGAGGGTAAAGGGGGCGGGGGGTTTGGTAGGAGGATGATTGGGTAGCGGGGGAACGCTGAAGTGATTTGGGTAGGG 197
ref_ND9         97 GGAGAAGTTGGATTGAGAGAGGGGGCGAGGGTAAAGGGGGCGGGGGGTTTGGTAGGAGGATGATTGGGTAGCGGGGGAACGCTGAAGTGATTTGGGTAGGG 197

                                                                                         *
29-13_ND9      198 AAGAAACATCGAGGAGTTTTGGGGGGAAGGATCCAAGGGGAGAGGGGTTGGGAGGAGATAGAGACGACAAGAGGGCAACCTTAATTTAGTCAAAATTTAAT 298
AnTat1.1_ND9   202 AAGAAACATCGAGGAGTTTTGGGGGGAAGGATCCAAGGGGAGAGGGGTTGGGAGGAGATAGAGACGACGAGAGGGCAACCTTAATTTAGTCAAAATTTAAT 302
BF_ND9         198 AAGAAACATCGAGGAGTTTTGGGGGGAAGGATCCAAGGGGAGAGGGGTTGGGAGGAGATAGAGACGACAAGAGGGCAACCTTAATTTAGTCAAAATTTAAT 298
SM_ND9         198 AAGAAACATCGAGGAGTTTTGGGGGGAAGGATCCAAGGGGAGAGGGGTTGGGAGGAGATAGAGACGACAAGAGGGCAACCTTAATTTAGTCAAAATTTAAT 298
PF_ND9         198 AAGAAACATCGAGGAGTTTTGGGGGGAAGGATCCAAGGGGAGAGGGGTTGGGAGGAGATAGAGACGACAAGAGGGCAACCTTAATTTAGTCAAAATTTAAT 298
ref_ND9        198 AAGAAACATCGAGGAGTTTTGGGGGGAAGGATCCAAGGGGAGAGGGGTTGGGAGGAGATAGAGACGACAAGAGGGCAACCTTAATTTAGTCAAAATTTAAT 298

                         *
29-13_ND9      299 TTCACCGTGTAATTCTAAATTTC 321
AnTat1.1_ND9   303 TTCACCATGTAATTCTAAATTTC 325
BF_ND9         299 TTCACCGTGTAATTCTAAATTTC 321
SM_ND9         299 TTCACCGTGTAATTCTAAATTTC 321
PF_ND9         299 TTCACCGTGTAATTCTAAATTTC 321
ref_ND9        299 TTCACCGTGTAATTCTAAATTTC 321
```

uS12m

```
29-13_uS12m      1 CTAATACACTTTTGATAACAAACTAAAGTAAAAAGGCGAGGATTTTTTGAGTGGGACTGGAGAGAAAGAGCCGTTCGAGCCCAGCCGGAACCGACGGAGA 100
AnTat1.1_uS12m   1 CTAATACACTTTTGATAACAAACTAAAGTAAAAAGGCGAGGATTTTTTGAGTGGGACTGGAGAGAAAGAGCCGTTCGAGCCCAGCCGGAACCGACGGAGA 100
BF_uS12m         1 CTAATACACTTTTGATAACAAACTAAAGTAAAAAGGCGAGGATTTTTTGAGTGGGACTGGAGAGAAAGAGCCGTTCGAGCCCAGCCGGAACCGACGGAGA 100
SM_uS12m         1 CTAATACACTTTTGATAACAAACTAAAGTAAAAAGGCGAGGATTTTTTGAGTGGGACTGGAGAGAAAGAGCCGTTCGAGCCCAGCCGGAACCGACGGAGA 100
PF_uS12m         1 CTAATACACTTTTGATAACAAACTAAAGTAAAAAGGCGAGGATTTTTTGAGTGGGACTGGAGAGAAAGAGCCGTTCGAGCCCAGCCGGAACCGACGGAGA 100
ref_uS12m        1 CTAATACACTTTTGATAACAAACTAAAGTAAAAAGGCGAGGATTTTTTGAGTGGGACTGGAGAGAAAGAGCCGTTCGAGCCCAGCCGGAACCGACGGAGA 100

29-13_uS12m    101 GCTTCTTTTGAATAAAAGGGAGGCGGGGAGGAGAGTTTCAAAAAGATTTGGGTGGGGGGAACCCTTTGTTTTGGTTAAAGAAACATCGTTTAGAAGAGAT 200
AnTat1.1_uS12m 101 GCTTCTTTTGAATAAAAGGGAGGCGGGGAGGAGAGTTTCAAAAAGATTTGGGTGGGGGGAACCCTTTGTTTTGGTTAAAGAAACATCGTTTAGAAGAGAT 200
BF_uS12m       101 GCTTCTTTTGAATAAAAGGGAGGCGGGGAGGAGAGTTTCAAAAAGATTTGGGTGGGGGGAACCCTTTGTTTTGGTTAAAGAAACATCGTTTAGAAGAGAT 200
SM_uS12m       101 GCTTCTTTTGAATAAAAGGGAGGCGGGGAGGAGAGTTTCAAAAAGATTTGGGTGGGGGGAACCCTTTGTTTTGGTTAAAGAAACATCGTTTAGAAGAGAT 200
PF_uS12m       101 GCTTCTTTTGAATAAAAGGGAGGCGGGGAGGAGAGTTTCAAAAAGATTTGGGTGGGGGGAACCCTTTGTTTTGGTTAAAGAAACATCGTTTAGAAGAGAT 200
ref_uS12m      101 GCTTCTTTTGAATAAAAGGGAGGCGGGGAGGAGAGTTTCAAAAAGATTTGGGTGGGGGGAACCCTTTGTTTTGGTTAAAGAAACATCGTTTAGAAGAGAT 200

29-13_uS12m    201 TTTAGAATAAGATATGTTTTT 221
AnTat1.1_uS12m 201 TTTAGAATAAGATATGTTTTT 221
BF_uS12m       201 TTTAGAATAAGATATGTTTTT 221
SM_uS12m       201 TTTAGAATAAGATATGTTTTT 221
PF_uS12m       201 TTTAGAATAAGATATGTTTTT 221
ref_uS12m      201 TTTAGAATAAGATATGTTTTT 221
```

Figure S2. Multiple sequence alignment of 18 maxicircle-encoded mRNAs and two rRNAs for five cell lines in this study, and annotated transcripts from Lister 427 (1). Related to Figure 1.

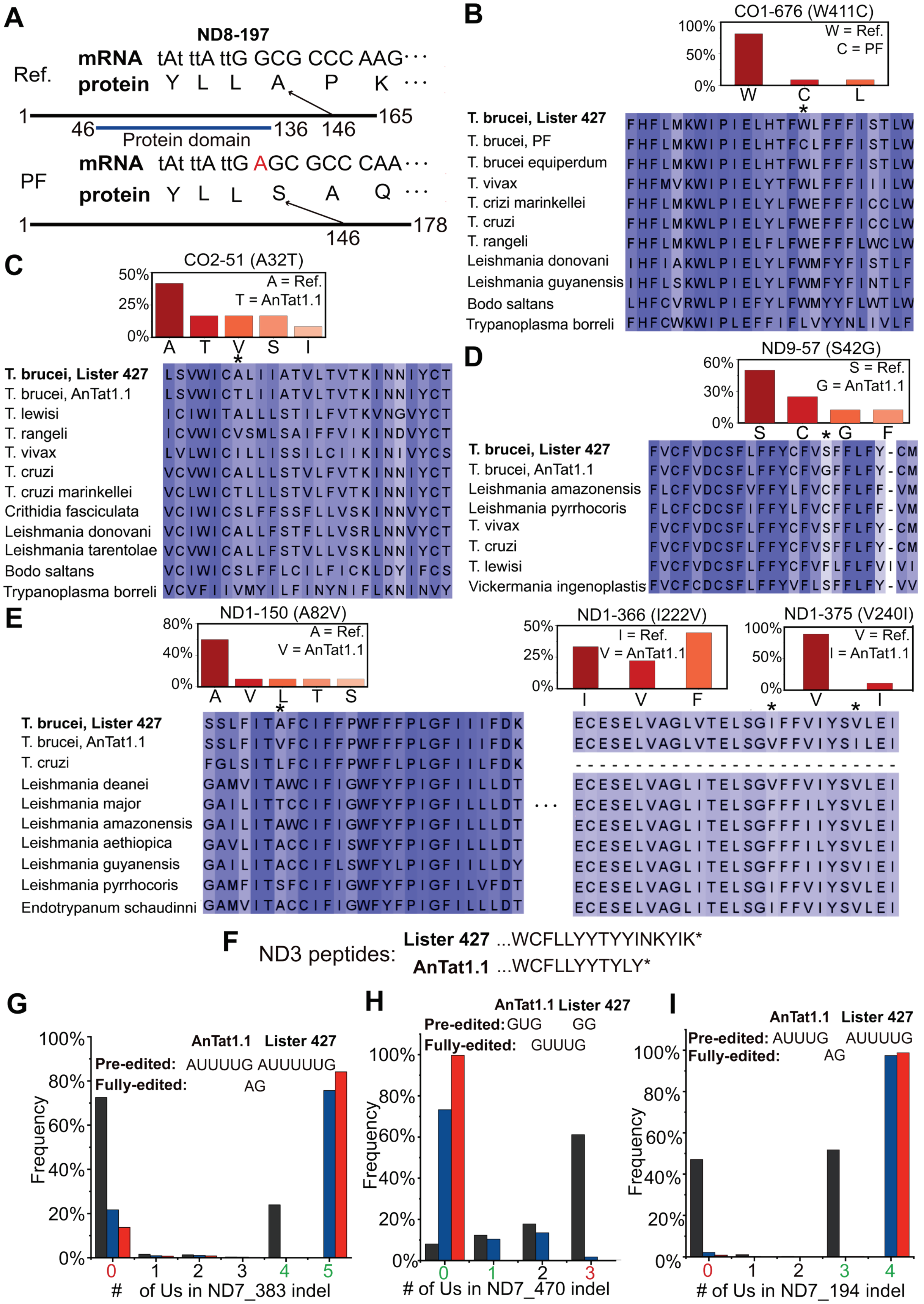
A
ND8-197
Ref.
mRNA tAt ttA ttG GCG CCC AAG···
protein Y L L A P K ···
1
165
46
136 146
Protein domain
PF
mRNA tAt ttA ttG AGC GCC CAA···
protein Y L L S A Q ···
1
178
146
B
CO1-676 (W411C)
100%
50%
0%
W = Ref.
C = PF
W C L
T. brucei, Lister 427 FHFLMKWIPIELHTFWLFFFISTLW
T. brucei, PF FHFLMKWIPIELHTFCLFFFISTLW
T. brucei equiperdum FHFLMKWIPIELHTFWLFFFISTLW
T. vivax FHFMVKWIPIELYTFWLFFFIIILW
T. crizi marinkellei FHFLMKWLPIELYLFWEFFFICCLW
T. cruzi FHFLMKWLPIELYLFWEFFFICCLW
T. rangeli FHFLMKWLPIELFLFWEFFFLWCLW
Leishmania donovani IHFIAKWLPIELYLFWMFYFISTLF
Leishmania guyanensis IHFLSKWLPIELYLFWMFYFINTLF
Bodo saltans LHFCVRWLPIEFYLFWMYYFLWTLW
Trypanoplasma borreli FHFCWKWIPLEFFIFLVYYNLIVLF
C
CO2-51 (A32T)
50%
25%
0%
A = Ref.
T = AnTat1.1
A T V S I
T. brucei, Lister 427 LSVWICALIIATVLTVTKINNIYCT
T. brucei, AnTat1.1 LSVWICTLIIATVLTVTKINNIYCT
T. lewisi ICIWITALLLSTILFVTKVNGVYCT
T. rangeli ICVWICVSMLSAIFFVIKINDVYCT
T. vivax LVLWICILLISSILCIIKINIVYCS
T. cruzi VCVWICALLLSTVLFVTKINNIYCT
T. cruzi marinkellei VCLWICTLLLSTVLFVTKINNIYCT
Crithidia fasciculata VCIWICSLFFSSFLLVSKINNVYCT
Leishmania donovani VCIWICALLFSTFLLVSRLNNVYCT
Leishmania tarentolae VCVWICALLFSTVLLVSKLNNIYCT
Bodo saltans VCIWICSLFFLCILFICKLDYIFCS
Trypanoplasma borreli VCVFIIVMYILFINYNIFLKNINVY
D
ND9-57 (S42G)
60%
30%
0%
S = Ref.
G = AnTat1.1
S C G F
T. brucei, Lister 427 FVCFVDCSFLFFYCFVSFFLFY-CM
T. brucei, AnTat1.1 FVCFVDCSFLFFYCFVGFFLFY-CM
Leishmania amazonensis FLCFVDCSFVFFYLFVCFFLFF-VM
Leishmania pyrrhocoris FLCFCDCSFIFFYLFVCFFLFF-VM
T. vivax FVCFVDCSFLFFYCFVSFFLFY-CM
T. cruzi FVCFVDCSFLFFYCFVSFFLFY-CM
T. lewisi FVCFVDCSFLFFYCFVFLFLFVIVI
Vickermania ingenoplastis FVCFCDCSFLFFYVFLSFFLFY-VV
E
ND1-150 (A82V)
80%
40%
0%
A = Ref.
V = AnTat1.1
A V L T S
ND1-366 (I222V)
50%
25%
0%
I = Ref.
V = AnTat1.1
I V F
ND1-375 (V240I)
100%
50%
0%
V = Ref.
I = AnTat1.1
V I
T. brucei, Lister 427 SSLFITAFCIFFPWFFFPLGFIIIFDK ECESELVAGLVTELSGIFFVIYSVLEI
T. brucei, AnTat1.1 SSLFITVFCIFFPWFFFPLGFIIIFDK ECESELVAGLVTELSGVFFVIYSILEI
T. cruzi FGLSITLFCIFFPWFFLPLGFIILFDK
Leishmania deanei GAMVITAWCIFIGWFYFPIGFILLLDT ECESELVAGLITELSGVFFVIYSVLEI
Leishmania major GAILITTCCIFIGWFYFPIGFILLLDT ··· ECESELVAGLITELSGFFFILYSVLEI
Leishmania amazonensis GAILITAWCIFIGWFYFPIGFILLLDT ECESELVAGLITELSGFFFIIYSVLEI
Leishmania aethiopica GAVLITACCIFISWFYFPIGFILLLDT ECESELVAGLITELSGFFFILYSVLEI
Leishmania guyanensis GAILITACCIFLSWFYLPIGFILLLDY ECESELVAGLITELSGFFFIIYSVLEI
Leishmania pyrrhocoris GAMFITSFCIFIGWFYFPIGFILVFDT ECESELVAGLITELSGIFFVIYSVIEI
Endotrypanum schaudinni GAMVITACCIFIGWFYFPIGFILLLDT ECESELVAGLITELSGIFFVIYSVLEI
F
ND3 peptides:
Lister 427 ...WCFLLYYTYYINKYIK*
AnTat1.1 ...WCFLLYYTYLY*
G
AnTat1.1 Lister 427
Pre-edited: AUUUUG AUUUUUG
Fully-edited: AG
Frequency
100%
80%
60%
40%
20%
0%
0 1 2 3 4 5
# of Us in ND7_383 indel
H
AnTat1.1 Lister 427
Pre-edited: GUG GG
Fully-edited: GUUUG
Frequency
100%
80%
60%
40%
20%
0%
0 1 2 3
# of Us in ND7_470 indel
I
AnTat1.1 Lister 427
Pre-edited: AUUUG AUUUUG
Fully-edited: AG
Frequency
100%
80%
60%
40%
20%
0%
0 1 2 3 4
# of Us in ND7_194 indel

Figure S3. Consequences of SNPs and indels in maxicircle protein-coding sequences and RNA editing patterns. Related to Figure 1.
(A) A PF cell line-specific T-to-A mutation in the ND8 gene leads to a potential frameshift.
(B) Multiple sequence alignment of protein sequences from PF and related species in the CO1 region with PF-specific nonsynonymous mutation. The frequency distribution of mutation-related amino acids is plotted above a multiple sequence alignment.
(C) Multiple sequence alignment of protein sequences from AnTat1.1 and related species in CO2 regions with AnTat1.1-specific nonsynonymous mutation. The frequency distribution of amino acid substitutions is plotted above the multiple sequence alignment.
(D) Multiple sequence alignment of protein sequences from AnTat1.1 and related species in the ND9 region with AnTat1.1-specific nonsynonymous mutation. The frequency distribution of amino acid substitutions is plotted above the multiple sequence alignment.
(E) Multiple sequence alignment of protein sequences from AnTat1.1 and related species in the ND3 region with AnTat1.1-specific nonsynonymous mutation. The frequency distribution of amino acid substitutions is plotted above the multiple sequence alignment.
(F) The protein-coding changes resulting from the indel in ND3. An asterisk indicates the termination of translation.
(G) RNA editing events at the T-less position 383 of the ND7 mRNA. In the x-axis, the number colored in red refers to the number of Us in the fully-edited sequence; numbers colored in green refer to Us in the pre-edited sequence.
(H) RNA editing events at the T-less position 470 of the ND7 mRNA.
(I) RNA editing events at the T-less position 194 of the ND7 mRNA.

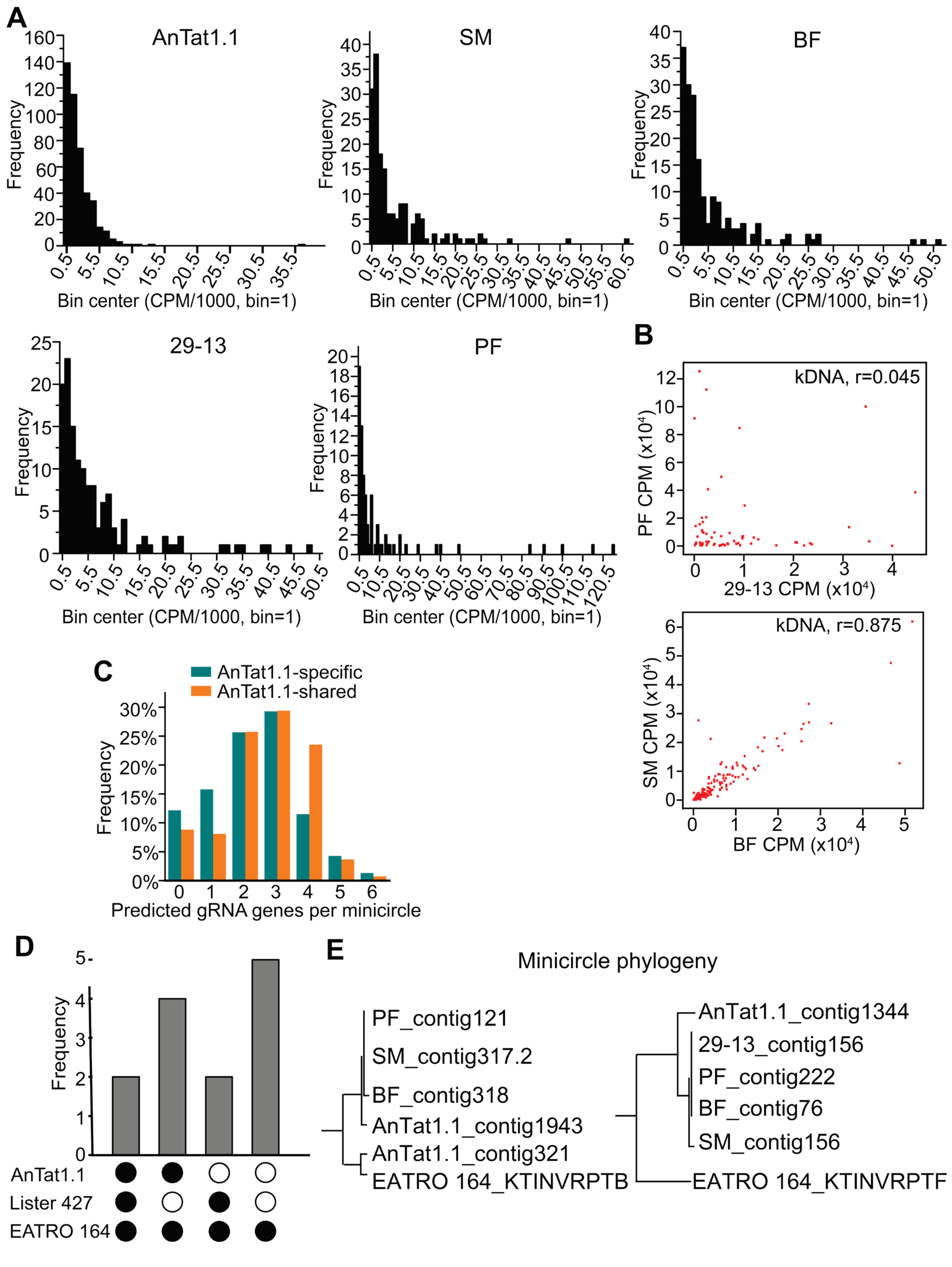

A
AnTat1.1
SM
BF
29-13
PF
Frequency
Bin center (CPM/1000, bin=1)
B
kDNA, r=0.045
PF CPM (x10⁴)
29-13 CPM (x10⁴)
kDNA, r=0.875
SM CPM (x10⁴)
BF CPM (x10⁴)
C
AnTat1.1-specific
AnTat1.1-shared
Predicted gRNA genes per minicircle
D
AnTat1.1
Lister 427
EATRO 164
E
Minicircle phylogeny
PF_contig121
SM_contig317.2
BF_contig318
AnTat1.1_contig1943
AnTat1.1_contig321
EATRO 164_KTINVRPTB
AnTat1.1_contig1344
29-13_contig156
PF_contig222
BF_contig76
SM_contig156
EATRO 164_KTINVRPTF

Figure S4. Characteristics of minicircle genomes across five *T. brucei* cell lines. Related to Figure 2.
(A) Frequency distribution of minicircle copy numbers normalized by counts per million (CPM) reads. The numbers on the x-axis indicate the center of each bin; for example, a CPM/1000 value of 140 minicircles falls between 0–1.
(B) Scatter plots of CPM-based minicircle abundance between two PCF (29-13 vs. PF) and BSF (SM vs. BF) cell lines.
(C) Frequency distribution of gRNA genes in minicircles shared between AnTat1.1 and Lister 427 strains, and those unique to AnTat1.1.
(D) Shared and unique minicircles among AnTat1.1 and Lister 427 from our studies, and EATRO 164 from Hong and Simpson (3).
(E) Minicircle phylogenetic trees for two minicircle classes shared by all three strains.

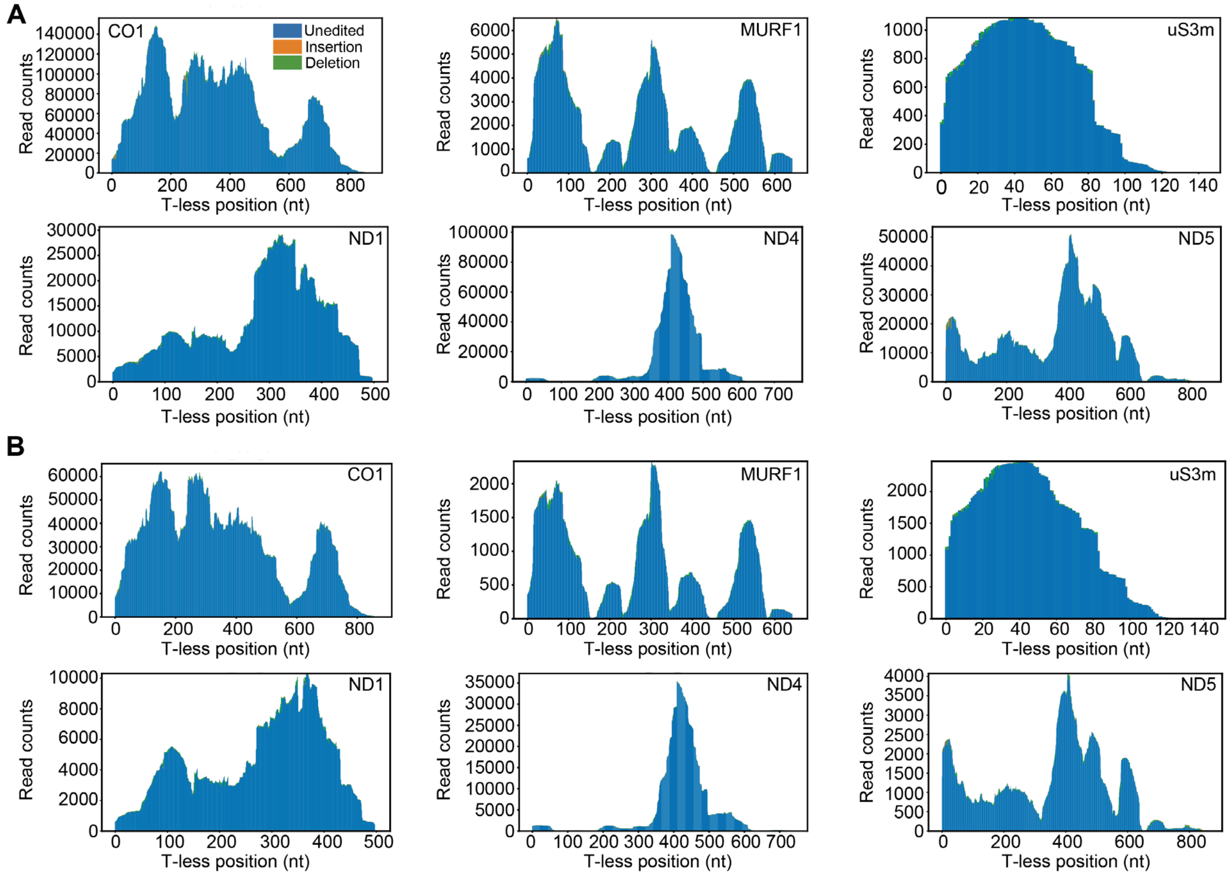

Figure S5. Analysis of potential RNA editing events in six "never-edited" transcripts in the BF (A) and PF (B) cell lines. Related to Figure 3.

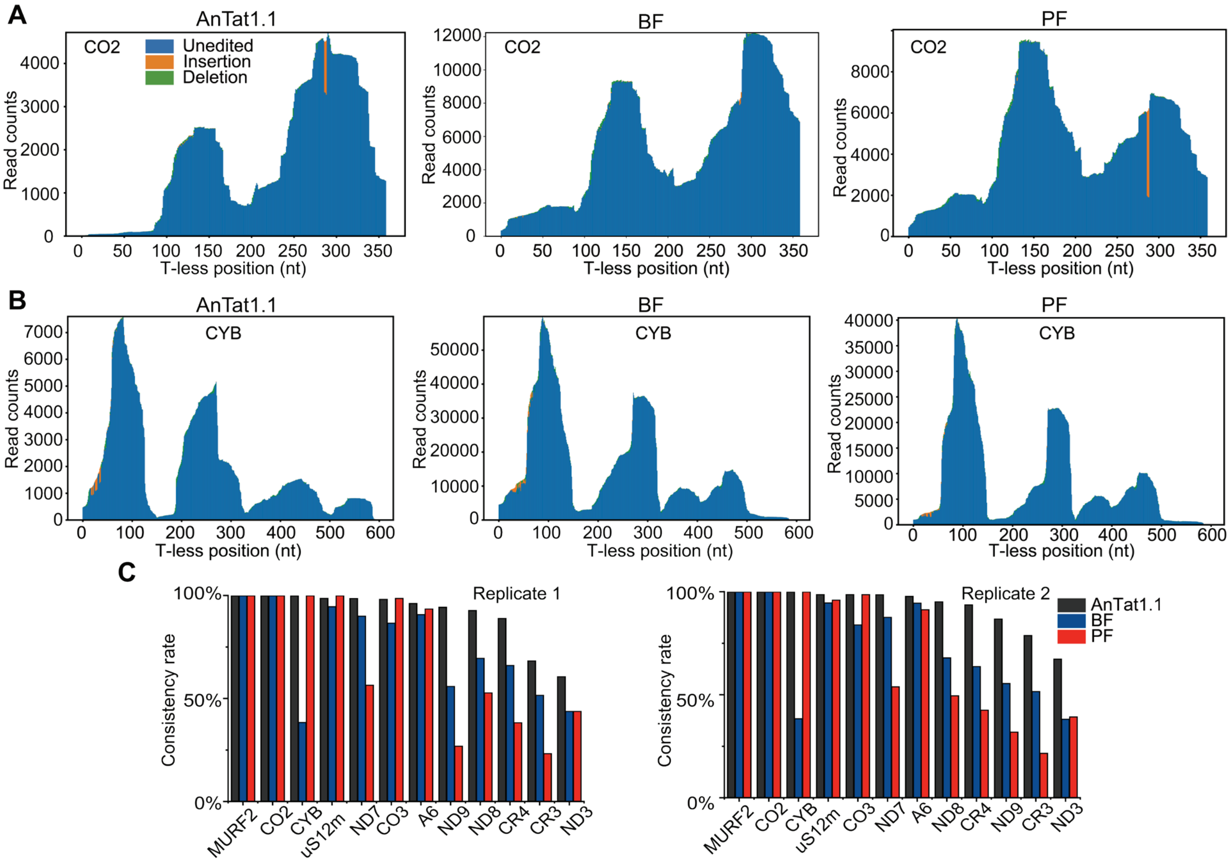

Figure S6. Per-site mRNA editing levels and editing consistency. Related to Figure 4.
Positional mapping of editing events in CO2 (A) and CYB (B) mRNA for BF and PF cell lines. The x-axis represents the 5′ to 3′ mRNA coordinates after removing encoded Us and Us inserted by editing. The y-axis shows the total read depth for pre-edited (blue), U-insertion (orange) and U-deletion (green) states of each nucleotide.
(C) Editing consistency rates for edited mRNAs in AnTat1.1, BF, and PF cell lines.

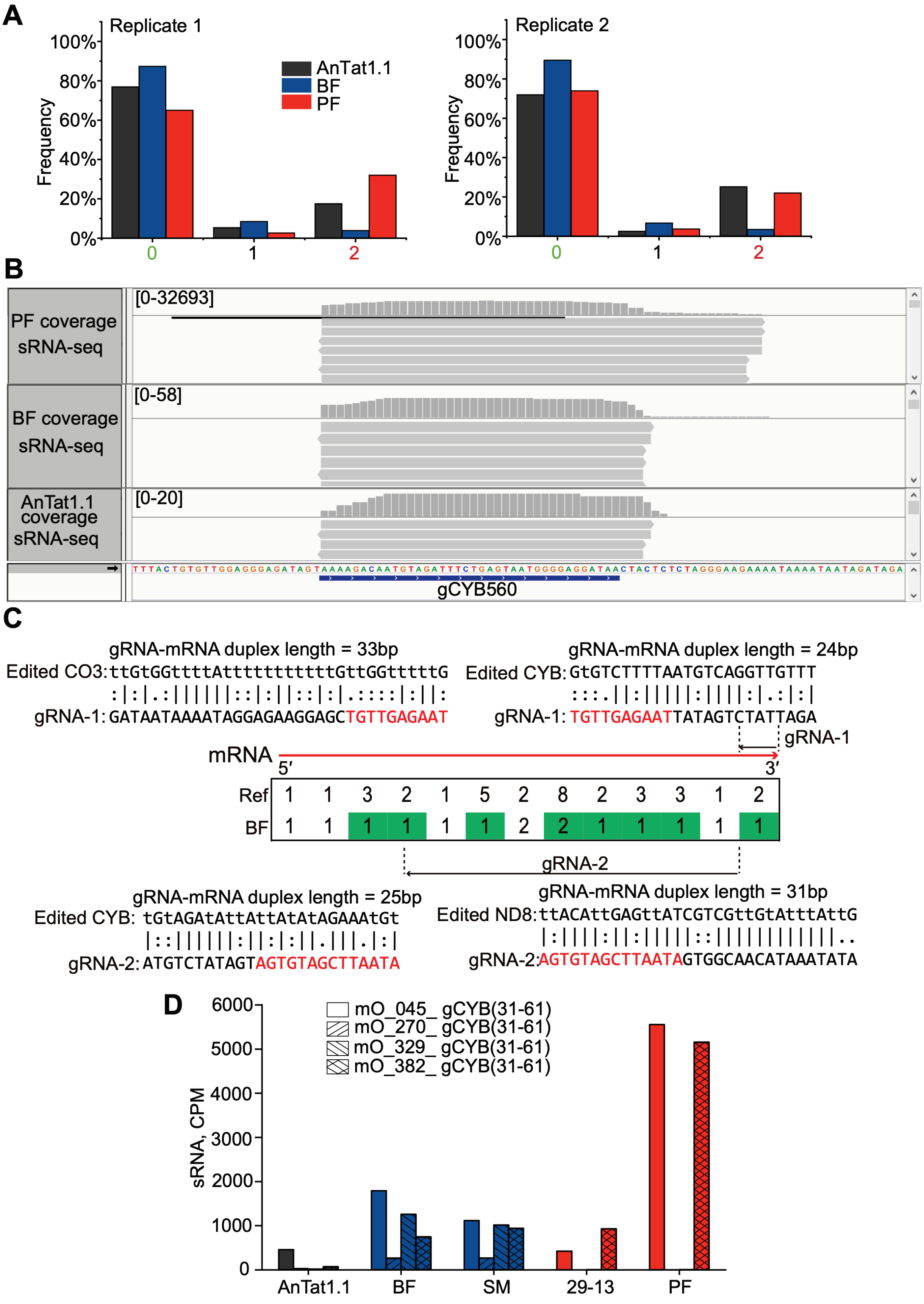

A
Replicate 1
100%
80%
60%
40%
20%
0%
Frequency
AnTat1.1
BF
PF
0
1
2
Replicate 2
B
PF coverage sRNA-seq
[0-32693]
BF coverage sRNA-seq
[0-58]
AnTat1.1 coverage sRNA-seq
[0-20]
TTTACTGTGTTGGAGGGAGATAGTAAAAGACAATGTAGATTTCTGAGTAATGGGGAGGATAACTACTCTCTAGGGAAGAAAATAAAATAATAGATAGA
gCYB560
C
gRNA-mRNA duplex length = 33bp
Edited CO3: ttGtGGttttAtttttttttttGttGGtttttG
gRNA-1: GATAATAAAATAGGAGAAGGAGCTGTTGAGAAT
gRNA-mRNA duplex length = 24bp
Edited CYB: GtGTCTTTTAATGTCAGGTTGTTT
gRNA-1: TGTTGAGAATTATAGTCTATTAGA
gRNA-1
mRNA
5′
3′
Ref 1 1 3 2 1 5 2 8 2 3 3 1 2
BF 1 1 1 1 1 1 2 2 1 1 1 1 1
gRNA-2
gRNA-mRNA duplex length = 25bp
Edited CYB: tGtAGAtAttAttAtAtAGAAAtGt
gRNA-2: ATGTCTATAGTAGTGTAGCTTAATA
gRNA-mRNA duplex length = 31bp
Edited ND8: ttACAttGAGttATCGTCGttGtAtttAttG
gRNA-2: AGTGTAGCTTAATAGTGGCAACATAAATATA
D
mO_045_ gCYB(31-61)
mO_270_ gCYB(31-61)
mO_329_ gCYB(31-61)
mO_382_ gCYB(31-61)
sRNA, CPM
6000
5000
4000
3000
2000
1000
0
AnTat1.1
BF
SM
29-13
PF

Figure S7. Expression level of initiation gRNA and 5′ gRNA in five cell lines. Related to Figure 5.

(A) Frequency distribution of U stretch for CYB 3′ most canonical editing site in two replicates of AnTat1.1, BF and PF cell lines.

(B) IGV rendering of small RNA-Seq reads mapping to minicircle encoding CYB560 gRNAs in PF, BF and AnTat1.1 cell lines.

(C) The most frequent length of U-insertions per site across 13 edited sites in CYB mRNA for reference and BF was plotted similar as Figure 5A. Sites where the most abundant U-stretch length differs from the reference in BF are highlighted in green. Examples of noncognate two gRNAs that may cause misediting at 3′ of CYB mRNA in BF cell line. Corresponding misediting regions were marked for two gRNAs. For both of these gRNAs, in addition to the alignment to CYB, another longer gRNA-mRNA complementary alignment exists with CO3 and ND8 mRNAs respectively.

(D) Expression levels of four CYB 5′ gRNAs in five cell lines.

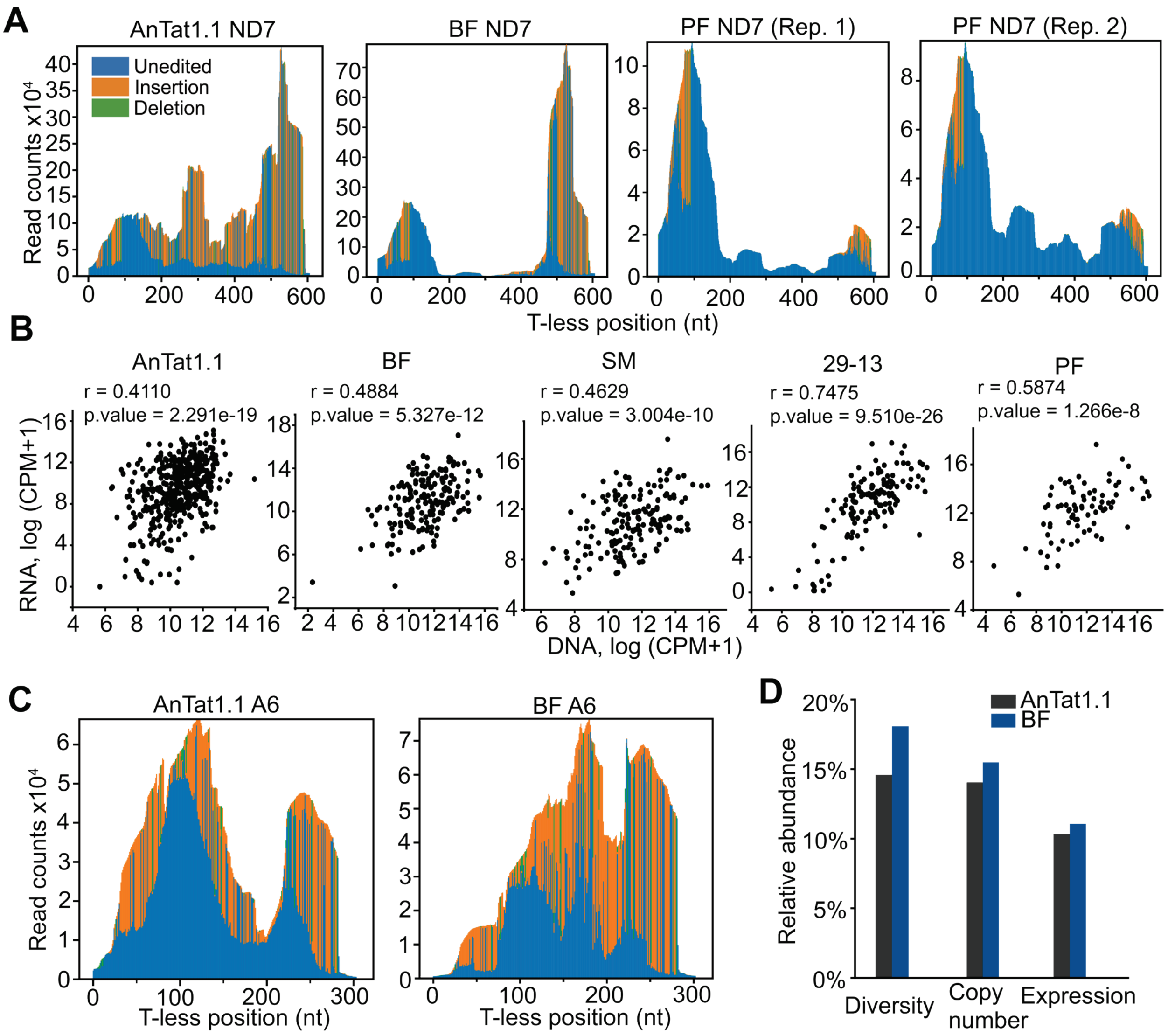

Figure S8. Guide RNA relative abundances association with editing levels. Related to Figure 6.
(A) Positional mapping of editing events in ND7 mRNA for BF and AnTat1.1 (Replicate 2) and PF cell lines (Replicates 1 and 2). The x-axis represents the 5′ to 3′ mRNA coordinates after removing encoded Us and Us inserted by editing. The y-axis shows the read depth for pre-edited (blue), U-insertion (orange) and U-deletion (green) states of each nucleotide.
(B) The correlation plot between copy number and gRNA expression level in five cell lines. The Pearson correlation coefficient and 2-tailed test of significance were used.
(C) Positional mapping of editing events in A6 mRNA for BF and AnTat1.1. The x-axis represents the 5′ to 3′ mRNA coordinates after removing encoded Us and Us inserted by editing. The y-axis shows the read depth for pre-edited (blue), U-insertion (orange) and U-deletion (green) states of each nucleotide.
(D) A6 gRNA classes (diversity), gene dosage (copy number) and RNA-seq-based abundance (expression) for AnTat1.1 and BF cell lines.

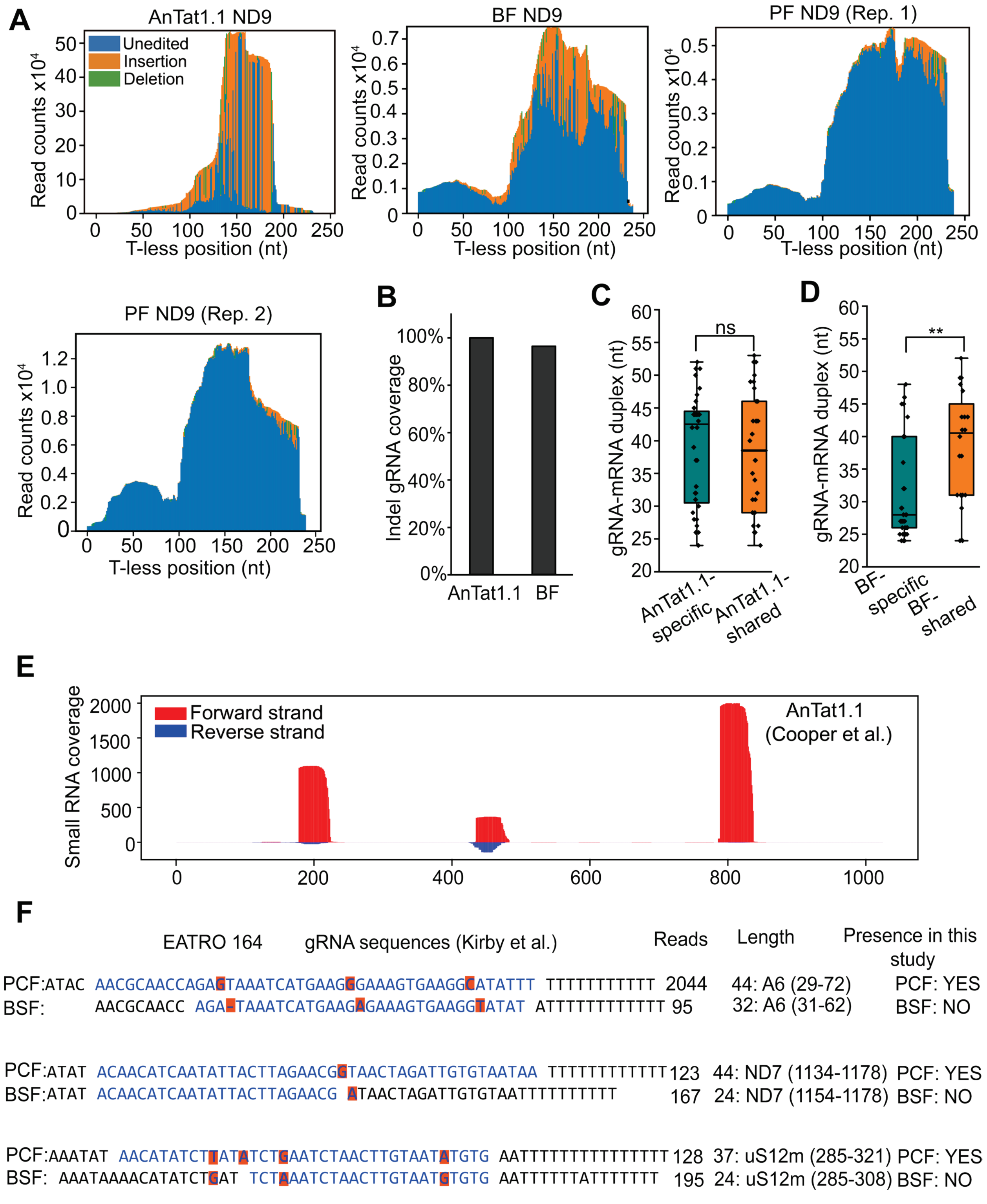

Figure S9. Longer gRNA-mRNA duplexes are associated with higher editing levels in ND9. Related to Figure 7.

(A) Positional mapping of editing events in ND9 mRNA for BF and AnTat1.1, two replicates each. The x-axis represents the 5′ to 3′ mRNA coordinates after removing encoded Us and Us inserted

by editing. The y-axis shows the read depth for pre-edited (blue), U-insertion (orange) and U-deletion (green) states of each nucleotide.
(B) Bar plots for gRNA coverage completeness for canonical ND9 mRNA editing sites in AnTat1.1 and BF cell lines.
(C) Boxplot of gRNA-mRNA duplex length for the gRNAs encoded in AnTat 1.1 strain-specific and shared minicircles.
(D) Boxplot of gRNA-mRNA duplex length for the gRNAs encoded in Lister 427 BF-specific and shared minicircles.
(E) Example of strain-specific differences in ND9 gRNA expression. RNA-Seq reads from AnTat1.1 from Cooper et al. (2) were mapped to the same minicircle as Figure 7F.
(F) The two forms (BF and PF) of EATRO 164 strain from Kirby et al. (4) showed different gRNA-mRNA complementary length; EATRO 164 PF gRNAs with longer lengths were also detected in our small RNA-Seq datasets.
Statistical significance was evaluated by the Mann-Whitney-Wilcoxon test in (C) and (D).

References

1. Ochsenreiter, T., Cipriano, M. and Hajduk, S.L. (2007) KISS: the kinetoplastid RNA editing sequence search tool. *RNA*, **13**, 1-4.
2. Cooper, S., Wadsworth, E.S., Ochsenreiter, T., Ivens, A., Savill, N.J. and Schnaufer, A. (2019) Assembly and annotation of the mitochondrial minicircle genome of a differentiation-competent strain of Trypanosoma brucei. *Nucleic Acids Res*, **47**, 11304-11325.
3. Hong, M. and Simpson, L. (2003) Genomic organization of Trypanosoma brucei kinetoplast DNA minicircles. *Protist*, **154**, 265-279.
4. Kirby, L.E., Sun, Y., Judah, D., Nowak, S. and Koslowsky, D. (2016) Analysis of the Trypanosoma brucei EATRO 164 Bloodstream Guide RNA Transcriptome. *PLoS Negl Trop Dis*, **10**, e0004793.

Supplementary tables legends

Supplementary Table S1. Summary of kDNA-Seq read mapping statistics to assembled maxicircle and minicircle for each cell line.

Supplementary Table S2. Detailed information of SNPs/indels in maxicircle transcripts.

Supplementary Table S3. Characteristics of minicircles in five cells lines.

Supplementary Table S4. Characteristics of predicted gRNA genes in five cell lines.

Supplementary Table S5. Editing ratio for each T-less position in maxicircle transcripts based on mRNA-seq.

Supplementary Table S6. Consistency of U-indels at canonical sites in maxicircle transcripts for AnTat1.1 and Lister 427 strains based on mRNA-seq.

Supplementary Table S7. Coverage statistics for predicted gRNA genes for AnTat1.1 and BF cell lines.
